# Contrasting Gene Decay in Subterranean Vertebrates: Insights from Cavefishes and Fossorial Mammals

**DOI:** 10.1093/molbev/msaa249

**Published:** 2020-09-28

**Authors:** Maxime Policarpo, Julien Fumey, Philippe Lafargeas, Delphine Naquin, Claude Thermes, Magali Naville, Corentin Dechaud, Jean-Nicolas Volff, Cedric Cabau, Christophe Klopp, Peter Rask Møller, Louis Bernatchez, Erik García-Machado, Sylvie Rétaux, Didier Casane

**Affiliations:** 1 CNRS, IRD, UMR Évolution, Génomes, Comportement et Écologie, Université Paris-Saclay, Gif-sur-Yvette, France; 2 Université Paris-Saclay, CEA, CNRS, Institute for Integrative Biology of the Cell (I2BC), 91198, Gif-sur-Yvette, France; 3 Institut de Génomique Fonctionnelle de Lyon, Université de Lyon, CNRS UMR 5242, Ecole Normale Supérieure de Lyon, Université Claude Bernard Lyon 1, Lyon, France; 4 SIGENAE, GenPhySE, INRAE, ENVT, Université de Toulouse, Castanet Tolosan, France; 5 INRAE, SIGENAE, Genotoul Bioinfo, MIAT UR875, Castanet Tolosan, France; 6 Natural History Museum of Denmark, University of Copenhagen, Copenhagen Ø, Denmark; 7 Department of Biology, Institut de Biologie Intégrative et des Systèmes, Université Laval, Québec City, QC, Canada; 8 Centro de Investigaciones Marinas, Universidad de La Habana, La Habana, Cuba; 9 CNRS, Institut des Neurosciences Paris-Saclay, Université Paris-Saclay, Gif-sur-Yvette, France; 10 UFR Sciences du Vivant, Université de Paris, Paris, France

**Keywords:** cavefishes, eye-specific genes, pseudogenization, machine learning, relaxed selection, molecular dating

## Abstract

Evolution sometimes proceeds by loss, especially when structures and genes become dispensable after an environmental shift relaxes functional constraints. Subterranean vertebrates are outstanding models to analyze this process, and gene decay can serve as a readout. We sought to understand some general principles on the extent and tempo of the decay of genes involved in vision, circadian clock, and pigmentation in cavefishes. The analysis of the genomes of two Cuban species belonging to the genus *Lucifuga* provided evidence for the largest loss of eye-specific genes and nonvisual opsin genes reported so far in cavefishes. Comparisons with a recently evolved cave population of *Astyanax mexicanus* and three species belonging to the Chinese tetraploid genus *Sinocyclocheilus* revealed the combined effects of the level of eye regression, time, and genome ploidy on eye-specific gene pseudogenization. The limited extent of gene decay in all these cavefishes and the very small number of loss-of-function mutations per pseudogene suggest that their eye degeneration may not be very ancient, ranging from early to late Pleistocene. This is in sharp contrast with the identification of several vision genes carrying many loss-of-function mutations in ancient fossorial mammals, further suggesting that blind fishes cannot thrive more than a few million years in cave ecosystems.

## Introduction

The evolution of organisms confronted with drastic environmental shifts results sometimes in profound phenotypic changes. Constructive evolution involved in adaptation to new environments, relying on novelties at the phenotypic and genetic levels, has attracted much interest (Rose and Lauder 1996). Nevertheless, it has become evident that regressive evolution, which is often nonadaptive and which occurs by loss of structures and functions and the corresponding genes, accounts for a nonnegligible component of the evolutionary process (Lahti et al. 2009; Albalat and Cañestro 2016). Selection may be involved in regressive evolution, as suggested by a QTL analysis of eye degeneration in a cavefish (Protas et al. 2007). More generally, the evolution of animals in a dark environment is a particularly useful model to analyze regressive evolution because it has occurred many times in many taxa and some challenges, such as the absence of light, are always present, which allows us to analyze convergent evolution. A dark environment is expected to release purifying selection on light-related genes such as those involved in vision, the circadian clock, and pigmentation. A careful examination of gene decay on a genome-wide scale has been performed in obligate fossorial mammals. It has been shown that several independent lineages with degenerate eyes have lost many genes involved in visual perception (Kim et al. 2011; Emerling and Springer 2014; Fang, Nevo, et al. 2014; Fang, Seim, et al. 2014; Emerling 2018).

In order to better understand the modalities, tempo, extent, and limits of molecular decay of light-related genetic systems at the scale of subterranean vertebrates, it is informative to compare gene decay in fossorial mammals with another large group of subterranean vertebrates, the cavefishes. Cavefishes represent the largest and most diverse group of cave vertebrates (Culver and Pipan 2009), but gene decay has not been surveyed on a genome-wide scale in relevant species. On the one hand, in the reference genome of the Mexican cavefish *Astyanax mexicanus*, very few pseudogenes have been found among the light-related genes (McGaugh et al. 2014). The retention of almost all of the eye-specific genes in this blindfish is a paradox under the hypothesis that it evolved millions of years ago, but would be expected under the hypothesis that all *A. mexicanus* cave populations are very recent (Fumey et al. 2018). On the other hand, in the genomes of three fishes belonging to the Chinese genus *Sinocyclocheilus*, that is, *S. grahami*, a surface fish with large eyes, *S. anshuiensis*, a blind cavefish, and *S. rhinocerous*, a small-eyed cavefish, many loss-of-function (LoF) mutations were found (Yang et al. 2016), but their tetraploid genomes hampered the identification of those LoF mutations related to the shift from surface to cave. After a whole-genome duplication (WGD), the pairs of paralogs resulting from this process (ohnologs) are most often redundant and one ohnolog can be pseudogenized without reducing fitness. Accordingly, several eye-specific genes contain LoF mutations in the large-eyed *S. grahami*. However, no thorough analysis of differential gene losses in relation to the level of eye degeneration has been carried out (Yang et al. 2016).

As the very recent origin of *A. mexicanus* cavefish populations and the tetraploidy of *Sinocyclocheilus* species did not allow the analysis of gene decay in cavefishes, it was necessary to examine the genomes of cavefishes that are millions of years old and which have not undergone a recent WGD. Species belonging to the genus *Lucifuga* (cave brotulas from the Bahamas and Cuba) were identified as good candidates. The genus *Lucifuga* is divided into two clades, one comprising only blind species and the other only small-eyed species (García-Machado et al. 2011). As no close surface relative has been identified and large genetic distances have been found between some species, within and between these clades, this genus of cavefishes is likely relatively ancient and the last common ancestor of the extant species was probably a cave-adapted fish (García-Machado et al. 2011; Hernández et al. 2020).

In order to compare eye-specific gene decay among cavefishes and between cavefishes and fossorial mammals, here we first sequenced the genomes of two Cuban cavefishes: one specimen, belonging to *L. dentata*, was blind and depigmented, the other one, belonging to *L. gibarensis* (Hernández et al. 2020), had small eyes and was pigmented. For all cavefishes for which genomes were available, and for some closely related surface species, we looked for likely LoF mutations (i.e., premature STOP codons, losses of START and STOP codons, losses of intron splice sites, and small indels leading to frameshifts) and for signatures of relaxed purifying selection on nonsynonymous mutations. The comparison of gene decay among cavefishes was extended to nonvisual opsin genes and two large sets of genes involved in the circadian clock and pigmentation. Contrasting patterns of gene loss indicated that eye-specific genes and nonvisual opsin genes have been much less constrained than circadian clock and pigmentation genes. The level of eye-specific gene decay was related to several factors such as the time the fishes have spent in the subterranean environment, the level of eye degeneration, and the level of genome ploidy. Nevertheless, no eye-specific genes with many LoF mutations were found in any cavefish, in sharp contrast to the highly degenerated eye-specific genes found in some fossorial mammals, suggesting that eye degeneration in the cavefishes is much more recent.

## Results

### Vision, Circadian Clock, and Pigmentation Gene Sets

**Fig. 1.**
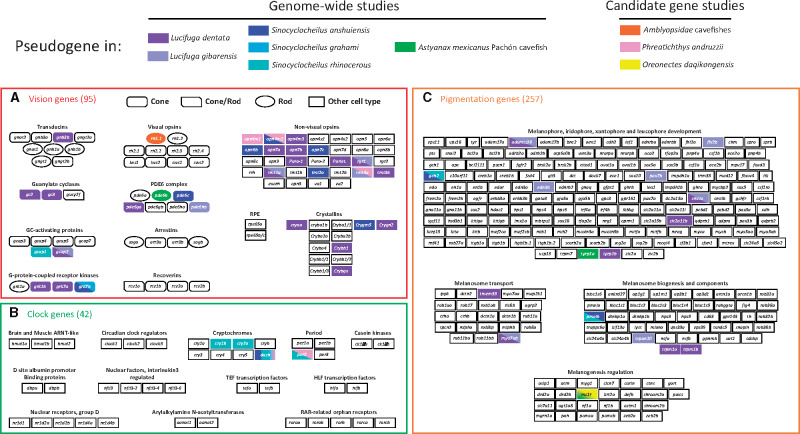
Gene sets and pseudogenes. (*A*) Vision genes. (*B*) Circadian clock genes. (*C*) Pigmentation genes. The number of genes in zebrafish for each set in shown in round brackets. Each box represents a gene, except the box rpe65b/c which represents two genes present only in zebrafish. The gene *mc1r* is duplicated in *Astyanax mexicanus* and only one copy carries a LoF mutation in the *A. mexicanus* cavefish genome. Pseudogenes are colored according the species in which they were found. In candidate gene studies, only few genes were examined, whereas most genes were studied in genome-wide analyses.

In the zebrafish, *Danio rerio*, we identified 63 eye-specific genes, that is, those expressed only in the eyes, and 32 genes coding for nonvisual opsins. As the same pattern of gene decay was observed for both gene sets, and because a large majority of these genes are involved in vision, they were pooled in a single group thereafter called vision genes (fig. 1 and supplementary fig. S1, Supplementary Material online). In addition, we defined two other gene sets, 42 genes involved in the circadian clock and 257 genes involved in pigmentation (fig. 1).

**Fig. 2.**
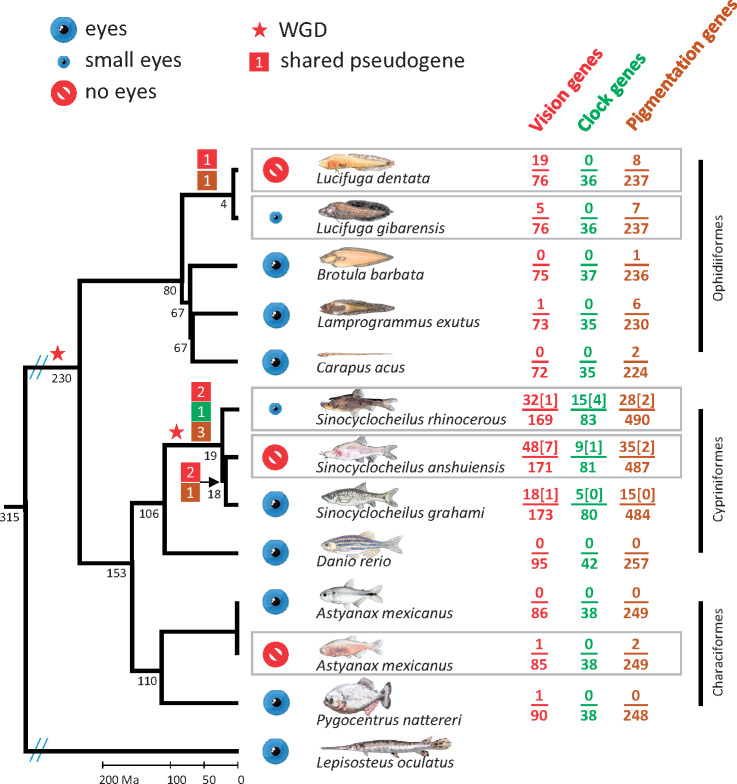
Phylogeny and pseudogene mapping. For each gene set, the number of pseudogenes found and the number of genes examined in a species are given to the right of the species name. For tetraploid species, the number of pairs of pseudogenes that are ohnologs is shown in square brackets. The number of shared pseudogenes is in boxes colored according to the gene set they belong to. Divergence times were obtained from timetree (http://www.timetree.org/), except between *Lucifuga* species (present study). Red star, whole-genome duplication.

An annotated draft genome and a transcriptome were obtained for one *L. dentata* cavefish (eyeless, supplementary fig. S2, Supplementary Material online) and the reads of one *L. gibarensis* cavefish (small-eyed, supplementary fig. S2, Supplementary Material online) were mapped on the genome of *L. dentata*. A detailed description of the results is given in supplementary data S1, Supplementary Material online. This allowed us to identify genes belonging to the gene sets defined above in the two *Lucifuga* species using the program EXONERATE and the coding sequences of the zebrafish. Using the same method, homologs were retrieved from the published genomes of three surface fishes (*Brotula barbata*, *Carapus acus*, and *Lamprogrammus exutus*) belonging to the same order, Ophidiiformes; from the published genomes of an *A. mexicanus* cavefish from Pachón cave and two surface fishes (a conspecific *A. mexicanus* surface fish and the piranha *Pygocentrus nattereri*), all belonging to the order Characiformes; and from the published genomes of two *Sinocyclocheilus* cavefishes (*S. anshuiensis* and *S. rhinocerous*) and a surface fish (*S. grahami*), all belonging to the order Cypriniformes (fig. 2).

As some genes have been duplicated in the terminal lineage leading to zebrafish (used as the reference to establish the gene lists), only one copy was expected to be found in other fishes. On the other hand, gene duplications, gene deletions as well as WGDs occurred in other lineages. Therefore, the number of genes retrieved was highly variable among genomes (fig. 2).

### Identification of LoF Mutations

Gene sequences were classified as complete if the whole-coding DNA sequence (CDS) could be retrieved or otherwise were classified as incomplete. Incomplete genes were discarded as it was most often impossible to know if they corresponded to sequencing gaps, assembly artifacts, or true large deletions. In the case of the *A. mexicanus* cavefish genome, among 45 missing exons in 19 incomplete genes, 36 exons of 16 genes could be amplified using primers designed with the exon sequences present in *A. mexicanus* surface fish genome. These data suggest that 80% of incomplete CDS in the *A. mexicanus* cavefish genome are not the result of large deletions but are assembly artifacts. Only complete sequences were further analyzed for the following LoF mutations: premature STOP codon, loss of the initiation codon, loss of the STOP codon, indel leading to a frameshift, mutations at intron splice sites. Other mutations in noncoding and coding sequences that could lead to a nonfunctional gene were not investigated as they cannot be readily identified. For example, several in-frame indel mutations were found in *A. mexicanus* but their functional consequences were not clear (Berning et al. 2019). The numbers of pseudogenes reported are therefore underestimates of the true numbers of nonfunctional genes.

#### Vision Pseudogenes

Among the list of 95 zebrafish vision genes, 76 genes were retrieved from the two *Lucifuga* spp. (cavefishes), 75 from *B. barbata*, 72 from *C. acus*, and 73 from *La. exutus* (surface fishes) (fig. 2 and supplementary fig. S1, Supplementary Material online). In eyed ophidiiform fishes, no vision pseudogene was found (*B. barbata* and *C. acus*) or only one (*gcap1* in *La. exutus*), but five pseudogenes were identified in the small-eyed cavefish *L. gibarensis* and 19 pseudogenes in the eyeless *L. dentata*. The nonvisual opsin *rgr1* was pseudogenized in the common ancestor of the two *Lucifuga* species, as the same mutation (at a splice site of intron 4) was found in both genomes (fig. 2 and supplementary fig. S1, Supplementary Material online). Examination of the read coverage of LoF mutations indicated that the specimen of *L. gibarensis* sequenced was heterozygous for LoF mutations found at two different sites in the *gcap2* gene (supplementary table S1, Supplementary Material online). In the transcriptome of *L. dentata*, transcripts corresponding to nine pseudogenes were found (three nonvisual opsins, three crystallins, and three genes involved in the phototransduction pathway), whereas no transcripts were found for 10 other pseudogenes (supplementary table S1, Supplementary Material online). In agreement with a recent WGD, two copies (ohnologs) of most vision genes were retrieved from the genomes of *Sinocyclocheilus* species (fig. 2 and supplementary fig. S1, Supplementary Material online). In the large-eyed *S. grahami*, ∼10% of the retrieved vision genes were pseudogenized (18/173 genes carried at least one LoF mutation), compared with 19% (32/169) in the small-eyed *S. rhinocerous* and 28% (48/171) in the eyeless cavefish *S. anshuiensis*. Only one pair of ohnologs was pseudogenized in the eyed *S. grahami* and the small-eyed *S. rhinocerous*, whereas seven pairs of ohnologs were pseudogenized in the blind *S. anshuiensis* (fig. 2 and supplementary fig. S1, Supplementary Material online). Two premature STOP codons and a frameshift in *sws1* were shared by the three *Sinocyclocheilus* species, as well as a mutation at the donor site of the third intron of *gc3*; *S. anshuiensis* and *S. grahami* shared a frameshift in *crygm5* and a frameshift plus a premature STOP codon in *grk7b* (fig. 2). Thus, most LoF mutations occurred in the terminal lineages leading to these species but a few LoF mutations occurred in different common ancestors.

In *A. mexicanus*, 86 and 85 vision genes were retrieved from the surface fish and the Pachón cavefish genome, respectively. Only one pseudogene was found in the Pachón cavefish genome, which is due to a deletion of 11 bp in *pde6b* (figs. 1 and 2).

In summary, although no or very few vision genes are pseudogenized in surface fishes and *A. mexicanus* cavefish, many vision pseudogenes were found in other cavefishes, up to 25% in *L. dentata*.

#### Circadian Clock Pseudogenes

Among the list of 42 zebrafish circadian clock genes, 36 genes were retrieved from *Lucifuga* genomes and 38 from *Astyanax* genomes. No pseudogene was found in these species. On the other hand, 5, 15, and 9 pseudogenes were identified among 80, 83, and 81 genes retrieved from the genomes of *S. grahami* (eyed), *S. rhinocerous* (small-eyed), and *S. anshuiensis* (blind), respectively. Both ohnologs of *cry-dash* were independently pseudogenized in *S. rhinocerous* and *S. anshuiensis*, a gene also pseudogenized in the Somalian cavefish *Phreatichthys andruzzii* (Zhao et al. 2018). Three other pairs of ohnologs (*cry1b*, *cry2a*, and *per2*) carried LoF mutations in *S. rhinocerous* (in *Ph. andruzzii*, the most abundant transcript of *per2* encodes a truncated protein; Ceinos et al. 2018). These data suggest that the circadian clock has most likely been impaired in *S. rhinocerous* but less so in *S. anshuiensis* (figs. 1 and 2).

#### Pigmentation Genes

Among the list of 257 zebrafish pigmentation genes, 237 genes were retrieved from *Lucifuga* genomes, 8 being pseudogenized in *L. dentata* and 7 in *L. gibarensis.* Although *smtla* and *myo7ab* have been lost independently in the two lineages, a premature STOP codon and an insertion are shared in *adamts20*. The number of pseudogenes in these cavefishes does not greatly differ from those found in some surface relatives, as 6 pseudogenes were identified among 230 pigmentation genes in *La. exutus* (figs. 1 and 2). Among *Sinocyclocheilus* species, only 3% (15/484) of pseudogenes were found in *S. grahami*, whereas 6% (28/490) were found in *S. rhinocerous* and 7% (35/487) in *S. anshuiensis* (fig. 2). Thus, after the WGD, the retention of pigmentation genes seems to have been much higher than among the vision genes in the two cavefishes but also in the surface fish (compared with 10%, 19%, and 28% of vision pseudogenes, respectively). Such a high percentage of retention of pigmentation genes has been found also after the Salmonid-specific WGD (Lorin et al. 2018). Strikingly, although no pair of ohnologs was found pseudogenized in *S. grahami*, the same two pairs of ohnologs (*gch2* and *pmelb*) were independently pseudogenized in *S. anshuiensis* and *S. rhinocerous* (fig. 1). The very small number of pseudogenes and the independent pseudogenization of the same genes in these two species suggest that only a limited subset of genes involved in pigmentation can be lost in these cavefishes.

In *A. mexicanus* cavefish, 2 pseudogenes were found among 249 pigmentation genes: *mc1r* which has already been reported in the literature (Gross et al. 2009) and which is also pseudogenized in the Chinese cavefish *Oreonectes daqikongensis* (Liu et al. 2019), and *tyrp1a* (figs. 1 and 2). The gene *mc1r* is duplicated in *A. mexicanus*, one copy is pseudogenized and the other could be functional (Gross et al. 2018).

The reliability of the LoF mutations identified above was assessed by different approaches. First, we found no or very few LoF mutations in at least one gene set in published genomes used in the present study (figs. 1 and 2), indicating that if some LoF mutations are artifacts, they are very rare in these genomes. In *L. dentata* and *L. gibarensis* genomes, no LoF mutations were found in circadian clock genes. Second, in *L. dentata*, LoF mutations were also found in transcripts when available. Third, the sequencing depth was high at the positions where LoF mutations were found in *L. dentata* and *L. gibarensis* (supplementary table S1, Supplementary Material online). Altogether, these lines of evidence suggest that LoF mutations identified in cavefish genomes are reliable.

Some LoF mutations listed above have been found in genes for which gene knockout experiments in model species suggest that their pseudogenization could be involved in eye regression, circadian clock disruption, or depigmentation. Other LoF mutations have been found in genes for which pseudogenization is involved in evolution of these traits in other cavefishes. A summary of the putative effects of these LoF mutations can be found in supplementary data S2, Supplementary Material online.

### Estimation of the Number of Neutral Vision Genes Based on the Distribution of LoF Mutations per Pseudogene in Cave Brotulas

**Fig. 3.**
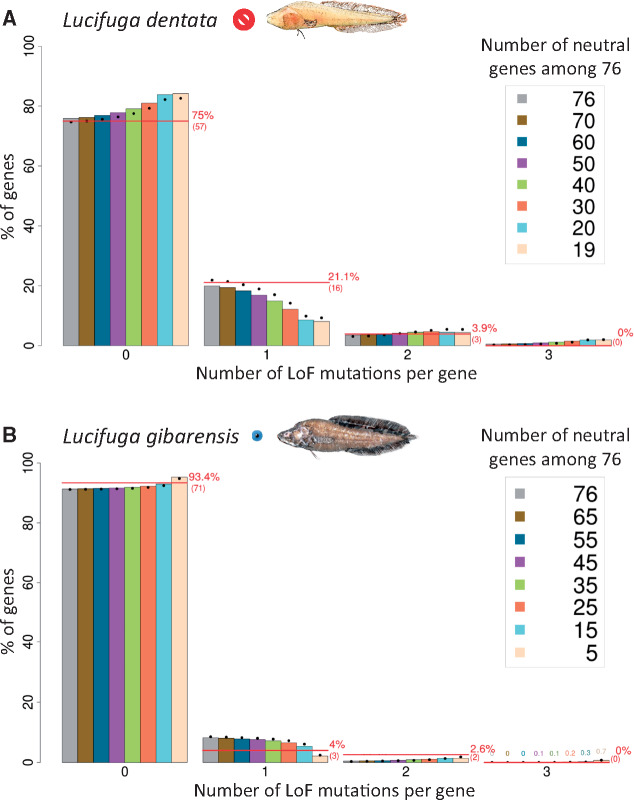
Observed and expected distributions of LoF mutations per gene. (*A*) *Lucifuga dentata*. (*B*) *L. gibarensis*. Red lines, observed distribution. Expected distributions were obtained using an analytical model (dots) and 10,000 simulations (histograms). Expected distributions were obtained for different numbers of neutral genes among 76 genes: between 19 and 76 in *L. dentata* and between 5 and 76 in *L. gibarensis*.

Among vision pseudogenes, some accumulated more than one LoF mutation, but in most of the cases only one LoF mutation was found (supplementary fig. S1, Supplementary Material online). In order to test if the whole set, or only a subset, of vision genes could accumulate LoF mutations in cavefishes, we compared the distribution of the number of LoF mutations per pseudogene with those expected under these different hypotheses. Expected distributions were obtained using either a simple analytical model assuming that all genes have the same probability to mutate, or a more complex model that takes into account that different genes do not have the same probability to mutate because they have different length and do not contain the same number of introns. In the latter case, the computation of expected distributions was based on simulations (see Materials and Methods for a detailed description of both methods). Very similar expected distributions were obtained with both approaches (fig. 3). This analysis could be performed only with *Lucifuga* species, as only one LoF mutation was found in *A. mexicanus* cavefish vision genes and a WGD allowed LoF mutations in many vision genes in *Sinocyclocheilus* species, including *S. grahami* which is a surface fish with large functional eyes.

In the eyeless *L. dentata*, 22 LoF mutations were distributed among 19 vision pseudogenes. More precisely, among the 76 genes retrieved, there were 57 genes without LoF mutation, 16 with 1 mutation, and 3 with 2 mutations. This distribution was compared with expected distributions obtained for different numbers of neutral genes ranging from 19 to 76 (fig. 3*A*). A better fit between the observed and expected distribution was found when at least 60 genes were assumed to be neutral sequences in which LoF could be found (fig. 3*A*). Using the same approach, we compared the distribution of the number of LoF mutations per pseudogene in the small-eyed *L*. *gibarensis* (71 genes without LoF mutation, 3 with 1 mutation, 2 with 2 mutations) with expected distributions assuming a number of neutral genes ranging from 5 to 76 (fig. 3*B*). In this case, the best fit was obtained when ∼15 vision genes were free to accumulate LoF mutations (fig. 3*B*). These results suggested that most genes, if not all, are dispensable in the blind *L. dentata* whereas only a small subset can be lost in the small-eyed *L. gibarensis*.

### Evidence of Relaxed Selection on Nonsynonymous Mutations in Cavefish Vision Genes

**Fig. 4.**
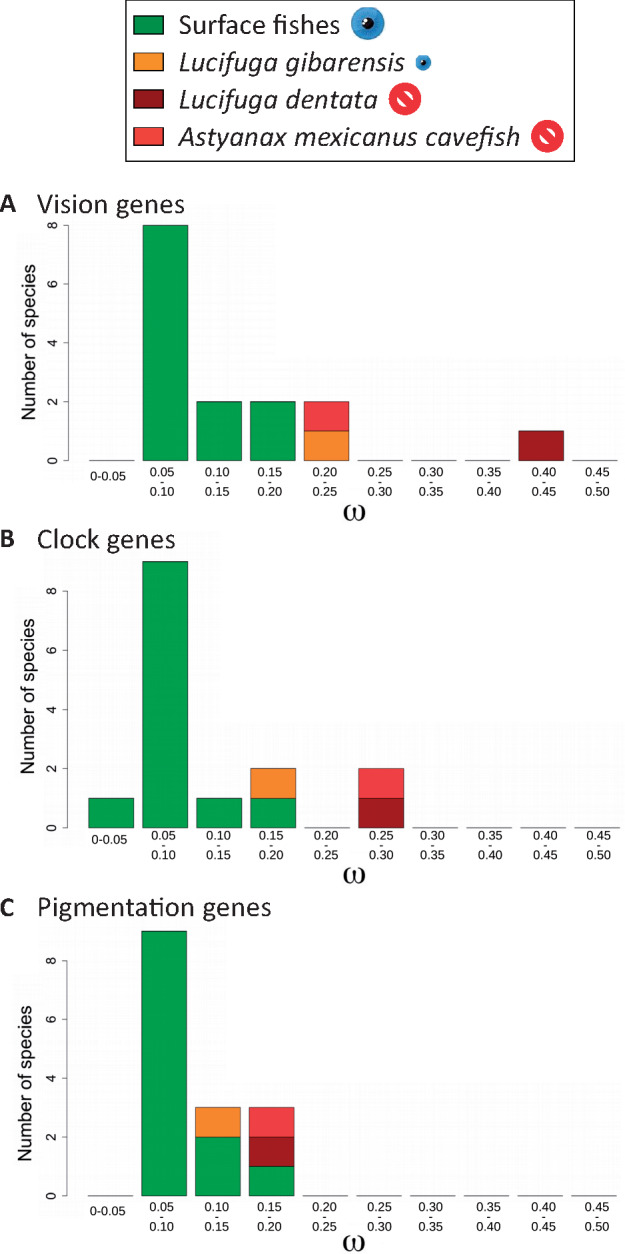
Distribution of *ω* in surface and cave fishes. (*A*) Vision genes. (*B*) Circadian clock genes. (*C*) Pigmentation genes. Surface fishes: *Astyanax mexicanus* surface morph, *Brotula barbata*, *Carapus acus*, *Danio rerio*, *Dichotomyctere nigroviridis*, *Gadus morhua*, *Gasterosteus aculeatus*, *Lamprogrammus exutus*, *Oreochromis niloticus*, *Oryzias latipes*, *Xiphophorus maculatus*, and *Pygocentrus nattereri*.

To confirm these findings on LoF mutations, we searched for other signatures of relaxed selection using methods based on changes in *ω* (the ratio of the mean number of nonsynonymous substitutions per nonsynonymous site to the mean number of synonymous substitutions per synonymous site, also known as d*n*/d*s* and Ka/Ks). This ratio is expected to be lower than one under purifying selection, equal to one under neutral evolution, and larger than one under adaptive selection. As gene divergence was <0.9% between *L. dentata* and *L. gibarensis*, and <0.2% between the two *A. mexicanus* morphs, the number of nucleotide differences per gene was very low and often no sequence change was observed (supplementary fig. S3, Supplementary Material online). Therefore, *ω* was computed for three sets of concatenated gene sequences (vision, circadian clock, and pigmentation genes), to obtain more reliable estimates and to compare the shift of selective pressure on these gene sets in different fish lineages. In order to obtain a reliable distribution of *ω* in surface fishes, gene sequences were retrieved from other fish genomes: tetraodon (*Dichotomyctere nigroviridis*), cod (*Gadus morhua*), stickleback (*Gasterosteus aculeatus*), spotted gar (*Lepisosteus oculatus*), tilapia (*Oreochromis niloticus*), medaka (*Oryzias latipes*), platyfish (*Xiphophorus maculatus*). Taking into account the known fish phylogenetic relationships (supplementary fig. S4, Supplementary Material online) and using the PAML package (Yang 2007), we compared three nested branch models assuming: 1) only one *ω*; 2) one *ω*_CF_ for blind cavefishes (*A. mexicanus* and *L. dentata*) and one *ω*_SF_ for the other fishes (two-ratio model); and 3) one *ω* for each branch (free-ratio model). For each data set, likelihood ratio tests suggested that the two-ratio model was better than the one-ratio model (*ω*_CF_ > *ω*_SF_) and the best model was the free-ratio model (supplementary table S2, Supplementary Material online). With the free-ratio model, *L. dentata* had the highest *ω* (0.409) for vision genes. For circadian clock genes, both *A. mexicanus* blind cavefish and *L. dentata* had the highest *ω* (0.29). For pigmentation genes, *ω* was similar in cave and surface fishes (fig. 4 and supplementary fig. S4, Supplementary Material online).

Independently, we analyzed the same sets of genes in *Sinocyclocheilus* species. For each species, ohnologs were concatenated into two series of gene sequences. For the two-ratio model, we assumed one *ω*_CF_ for the blind cavefish, *S. anshuiensis*, and one *ω*_SF_ for the two-eyed species, *S. rhinocerous* and *S. grahami*. For each data set, likelihood ratio tests suggested that the two-ratio model was better than the one-ratio model (*ω*_CF_ > *ω*_SF_) and the best model was the free-ratio model (supplementary table S2, Supplementary Material online). With the free-ratio model, *ω* was higher in the blind *S. anshuiensis* (0.36) than in the small-eyed *S. rhinocerous* (0.32) and the eyed *S. grahami* (0.23) with vision genes. With circadian clock genes, *ω* was higher in the blind *S. anshuiensis* (0.38) and the small-eyed *S. rhinocerous* (0.37) than in the eyed *S. grahami* (0.25). With pigmentation genes, *ω* was higher in the small-eyed *S. rhinocerous* (0.32) and the blind *S. anshuiensis* (0.29) than in the eyed *S. grahami* (0.25) (supplementary fig. S4, Supplementary Material online). Thus, *ω* was consistently higher in cavefishes than in surface fishes, the shift being larger for vision genes than for circadian clock and pigmentation genes. These results suggest a larger reduction of purifying selection on the vision gene set than on the other gene sets in cavefishes.

We then used another approach implemented in RELAX which computes the values and distribution of three *ω* using a branch-site model, the convergence of the three *ω* toward one in a lineage being a signature of relaxed purifying selection (Wertheim et al. 2015). The magnitude of convergence depends on a parameter, k, which tends to zero as selection tends to complete relaxation. RELAX detected relaxed purifying selection on *L. dentata* vision genes with an important shift toward *ω* = 1 as *k* = 0.2, and this was also true to a lesser extent in *A. mexicanus* cavefish (*k* = 0.5). For pigmentation genes, the largest shift was also observed in *L. dentata* (*k* = 0.48). No shift was observed with cavefish circadian clock genes, suggesting that most of these genes are under strong purifying selection in cavefishes (supplementary figs. S5–S7, Supplementary Material online).

**Fig. 5.**
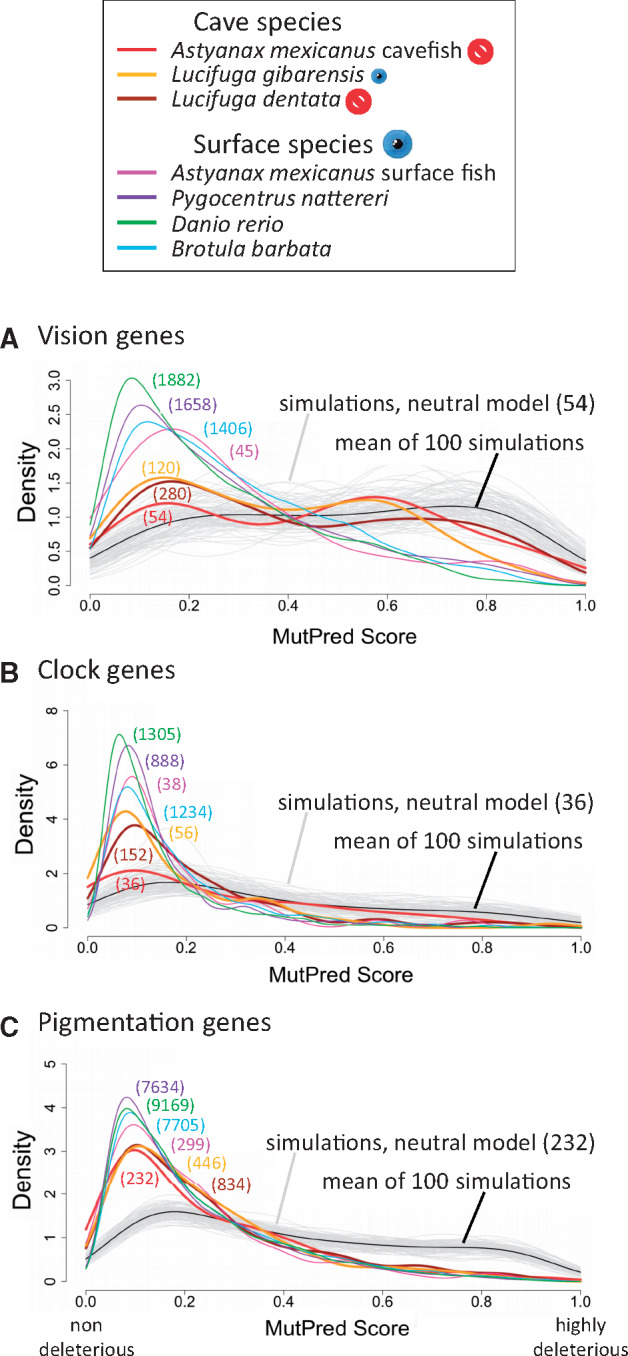
Distributions of MutPred2 scores in different fish lineages and in simulations of mutations without selection. This score ranges between 0 (nondeleterious mutation) and 1 (highly deleterious mutation). The number of mutations in each lineage is given in round brackets. One hundred simulations were performed on each gene set. In each simulation, 54 nonsynonymous mutations were generated in vision genes, 36 in circadian clock genes, and 232 in pigmentation genes, those numbers corresponding to the numbers of nonsynonymous mutations found in *Astyanax mexicanus* cavefish.

Finally, with the aim of finding additional and independent evidence of relaxed purifying selection in cavefishes, in particular on *A. mexicanus* vision genes for which the number of mutations is particularly low and thus, the estimate of *ω* was not accurate, we developed a novel approach. First, nonsynonymous mutations in different lineages were inferred using the aaml program from the PAML package. The deleterious impact of these mutations was then estimated using a machine learning method implemented in MutPred2 (Pejaver V, Urresti J, Lugo-Martinez J, Pagel KA, Lin GN, Nam H-J, Mort M, Cooper DN, Sebat J, Iakoucheva LM, Mooney SD and Radivojac P 2017. MutPred2: inferring the molecular and phenotypic impact of amino acid variants, unpublished data. Available from: https://www.biorxiv.org/content/10.1101/134981v1 ) which gives a score between 0 (not deleterious) and 1 (very deleterious). The kernel density estimation of the distributions of the scores in vision, circadian clock, and pigmentation genes were obtained for each terminal lineage leading to surface fishes and cavefishes, as well as for computer simulations of substitutions in the same gene sets under a neutral model. With all surface fishes, the kernel density estimation was similarly right-skewed (fig. 5), suggesting that most substitutions in surface fishes have a low impact on fitness. This was confirmed by the shape of the distribution of the scores in simulations of substitutions without selection (equivalent to the distribution of the scores before selection) which was very different to those of surface fishes, that is almost uniform, suggesting that the most deleterious mutations had been removed by selection in surface fishes. Before selection, the score distribution was slightly different for the different sets of genes, probably reflecting different selective constraints on the sequences belonging to these gene sets (fig. 5*A–C*, gray and black curves). The transitions/transversions (Ts/Tv) ratio used in simulations of substitutions under a neutral model had no impact on the distribution of the scores (supplementary fig. S8, Supplementary Material online). In the cavefishes on the other hand, the score distribution was very variable, depending on the cavefish species and the set of genes (fig. 5*A–C*). Pairwise comparisons of empirical cumulative distribution functions (ECDF) were performed using the nonparametric Kolmogorov–Smirnov (KS) test (supplementary fig. S9, Supplementary Material online). The same approach was attempted using Grantham’s distances (Grantham 1974) instead of MutPred2 scores but the contrast between the distributions of the distances with and without selection was much less discriminant and not analyzed further (supplementary fig. S10, Supplementary Material online).

In order to refine the analysis of the score distribution in cavefishes, mixtures of different proportions of substitutions picked from two distributions, one under neutral evolution (from the simulations) and the other under purifying selection (in the lineage leading to zebrafish) were also obtained to compare with cavefish distributions (supplementary fig. S11, Supplementary Material online).

#### Vision Genes

For the *A. mexicanus* cavefish (red curve, fig. 5*A*), the distribution was not statistically different from that expected if all substitutions were neutral in this lineage (KS test, *P* = 0.2; supplementary fig. S9, Supplementary Material online), yet the best fit was with a mixture distribution with 24% of substitutions from the distribution under purifying selection (supplementary fig. S11, Supplementary Material online). For *L. dentata* (brown curve, fig. 5*A*) and *L. gibarensis* (orange curve, fig. 5*A*), distributions departed from the neutral distribution (KS test, *P* = 1.4 × 10^−5^ and *P* = 4 × 10^−6^, respectively) (supplementary fig. S9, Supplementary Material online) and the best fit was obtained with 34% and 60% of the substitutions from the distribution under purifying selection, respectively (supplementary fig. S11, Supplementary Material online). For all *Sinocyclocheilus* species, the score distribution was different from those of surface fishes (supplementary fig. S12, Supplementary Material online). This was even true for the eyed *S. grahami*, most likely because after the WGD, purifying selection on nonsynonymous mutations was partially relaxed on one or both ohnologs. However, the ECDF of *S. rhinocerous* and *S. anshuiensis* were more shifted toward the neutral distribution than the ECDF of *S. grahami*, suggesting that the two cavefishes experienced a more neutral regime than the surface fish (supplementary fig. S12, Supplementary Material online).

#### Circadian Clock Genes

No cavefish ECDF fit with the expected distribution under neutral evolution (fig. 5*B* and supplementary fig. S9, Supplementary Material online). However, the ECDF of *A. mexicanus* cavefish was different from those of surface fishes and the best fit was obtained with a mixture of 59% of the substitutions from the distribution under purifying selection (fig. 5*B* and supplementary fig. S11, Supplementary Material online). For *L. dentata* and *L. gibarensis*, the best fit involved the mixture of 69% or 93% of the substitutions from the distribution under purifying selection (fig. 5*B* and supplementary fig. S11, Supplementary Material online). In accordance with the number of pseudogenes found in *S. rhinocerous*, the ECDF was the closest to a neutral distribution among the three *Sinocyclocheilus* species, with the best fit found with a mixture of 39% of substitutions from the distribution under purifying selection (supplementary figs. S12 and S13, Supplementary Material online).

#### Pigmentation Genes

No cavefish ECDF fitted with the expected distribution under neutral evolution (fig. 5*C*). All cavefish distributions were very similar to those of surface fishes, in accordance with the hypothesis that very few pigmentation genes can be lost, even after cave colonization and/or genome duplication (see also supplementary figs. S9 and S11–S13, Supplementary Material online).

In summary, three different approaches consistently suggested different levels of relaxed purifying selection on the set of vision genes that are related with the levels of eye degeneration in cavefishes, whereas most circadian clock and pigmentation genes remained under strong purifying selection.

### Dating Relaxation of Purifying Selection on Vision Genes in *L. dentata*

In order to make compatible the results suggesting that most vision genes are dispensable and those suggesting that selection is not totally relaxed in the *L. dentata* lineage, we postulated two successive periods of evolution, one under purifying selection followed by another under completely relaxed selection. Three independent approaches were used to estimate when purifying selection was relaxed in the *L. dentata* lineage.

**Fig. 6.**
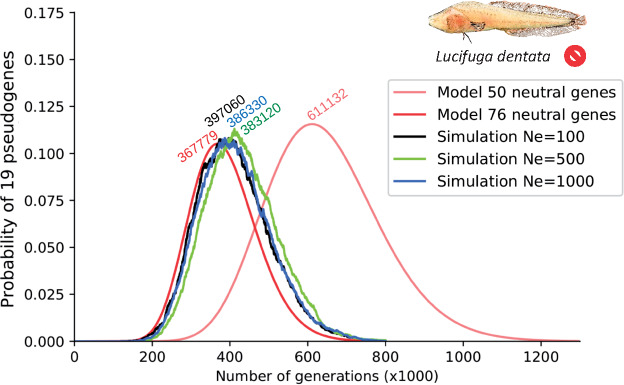
Probability of finding 19 vision pseudogenes in *Lucifuga dentata* according to the period of neutral evolution. Red and pink lines, based on an analytical model assuming 76 and 50 neutral genes, respectively; other lines, estimates based on 10,000 simulations, assuming 76 neutral genes and taking into account the length and number of introns in each vision gene and considering different effective population sizes. The number of generations for which the highest probability was found is reported above each line.

First, we used the number of vision pseudogenes and an estimate of the LoF mutation rate per gene per generation. Using the numbers of premature STOP codons, frameshifts, losses of splice site, losses of START and STOP codons in this species, and the method described in supplementary data S3, Supplementary Material online, we estimated the relative rates of these LoF mutations in *Lucifuga* species: 0.031, 0.0143, 0.0212, 0.0028, and 0.0023 µ, respectively (where *µ* is the nucleotide mutation rate per site per generation). In vertebrates, very few estimates of *µ* are available, the most accurate value being ∼10^−8^ in humans (Roach et al. 2010). Two independent values were recently obtained in cichlid fishes, one lower (3.5 × 10^−9^) and one higher (6.6 × 10^−8^) (Recknagel et al. 2013; Malinsky et al. 2018). Assuming that *µ* = 10^−8^ and taking into account that the average length of a vision gene equals 1,091 bp, the LoF mutation rate per gene per generation was 0.78 × 10^−6^. With the analytical model described in Materials and Methods and this rate of pseudogenization, the highest probability of finding 19 pseudogenes among 76 neutral genes was obtained with a complete relaxation of selection starting 367,779 generations ago (probability > 5% in a range between 273,990 and 480,980 generations) (fig. 6, red curve). Assuming that only 50 vision genes could accumulate LoF mutations, this time was pushed back to 611,132 (445,950–813,580) generations (fig. 6, pale red curve). Simulations were also performed to take into account variations of gene length and number of introns per gene, codon usage, transition/transversion ratio (*r* = 4.57), and effective population size (N_e_) in a range between 100 and 1,000. These simulations gave estimates very similar to those obtained with the analytical model, showing that the effects of N_e_ and per gene LoF mutation rate variation due to differences in gene length and number of introns were marginal (fig. 6, black, green, and blue curves; only simulations assuming 76 neutral genes are shown).

Second, two dating methods were used (Li et al. 1981; Meredith et al. 2009), both based on the hypothesis of a shift of *ω* from a value lower than 1 to 1 after purifying selection was relaxed in a lineage. We assumed a divergence time of 80 My between *Lucifuga* and *Brotula* (http://www.timetree.org/) reflecting their rather distant position in two different families, Bythitidae and Ophidiidae, within Ophidiiformes (Møller et al. 2016). Vision genes of *Lucifuga* species and *B. barbata* were individually aligned and alignments concatenated. With one method (Li et al. 1981), the divergence time between *L. dentata* and *L. gibarensis* was estimated to 4.1 Ma and the time since nonfunctionalization of vision genes in *L. dentata* was 1.5 My (table 1). With the other method (Meredith et al. 2009), *ω* was estimated to 0.27 in the lineage leading to *L. gibarensis* and 0.50 in the lineage leading to *L. dentata*. Assigning these ratios respectively to functional branches and a mixed branch, the time since nonfunctionalization was estimated to 1.3 My (table 1).

**Table 1.** Estimates of the Period of Neutral Evolution of *Lucifuga dentata* Vision Genes.

	Method	Tn/Td	Tn
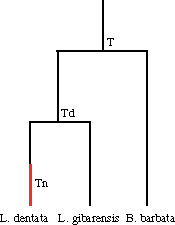	Meredith et al. (2009)	0.32	1.3 My
	Based on Pejaver et al. (unpublished data)	0.34	1.4 My
	Based on Li and Nei (1977)	n/a	367,779 g
	Li et al. (1981)	0.36	1.5 My

Note.—Tn, period of neutral evolution; Td, divergence time of *L. dentata* and *L. gibarensis*; T, divergence time of *Brotula barbata* and *Lucifuga* spp.; My, million years; n/a, not applicable; g, generations.

Third, we assumed that in the lineage leading to *L. dentata*, there is a mixture of 66% of the mutations that accumulated under completely relaxed selection and 34% under purifying selection (supplementary fig. S11, Supplementary Material online), *ω* = 0.27 under purifying selection (that is *ω* estimated in *L. gibarensis*), *ω* = 1 under completely relaxed selection and the divergence between *L. dentata* and *L. gibarensis* occurred 4.1 Ma (estimated above). Using the method described in Materials and Methods, we obtained an estimate, 1.4 My, of the age of relaxation of purifying selection (table 1). Thus, different methods for dating relaxation of purifying selection in the *L. dentata* lineage converged to ∼1.3–1.5 Ma. These estimates are compatible with the estimated time since vision genes could accumulate LoF mutations, that is ∼370,000 generations ago, if we assume a generation time of ∼4 years in *L. dentata*.

### Distribution of LoF Mutations in Vision Pseudogenes of Cavefishes versus Fossorial Mammals

**Fig. 7.**
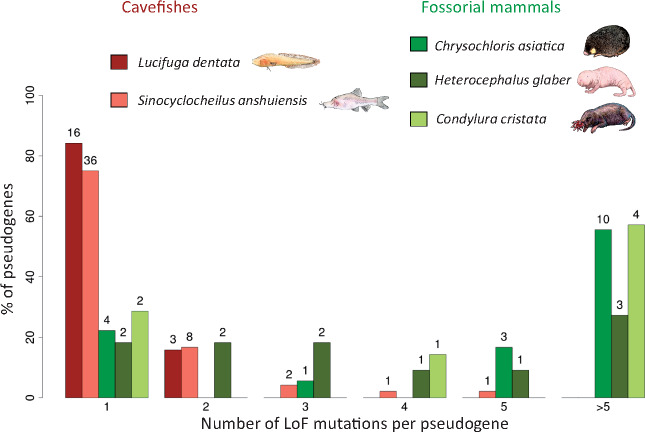
Distributions of the number of LoF mutations per vision pseudogene in blind cavefishes and fossorial mammals. The number of pseudogenes is given above the bar.

An extensive study of the regression of visual protein networks in three fossorial mammals, the Cape golden mole *Chrysochloris asiatica*, the naked mole-rats *Heterocephalus glaber*, and the star-nosed mole *Condylura cristata*, has been published (Emerling and Springer 2014). From this publication, we retrieved the number of pseudogenes, their names, and the number of LoF mutations per pseudogene in the three species. In the Cape golden mole, 18 pseudogenes were found among 65 vision genes, whereas only 11 pseudogenes were found in the naked mole-rat and seven in the star-nosed mole. Several independent LoF mutations were found in orthologous vision genes of fossorial mammals and cavefishes. The distributions of LoF mutations per pseudogene in these mammals and two blind cavefishes (*L. dentata* and *S. anshuensis*) were compared (fig. 7). *Astyanax mexicanus* cavefish, which is also blind, was not included in this comparison because there is only one LoF mutation in one gene in this species. The distributions were sharply contrasted when comparing mammals and fishes. In fossorial mammals, most pseudogenes carried several LoF mutations, up to 28 mutations in two pseudogenes of the golden mole and 54 mutations in a single pseudogene of the star-nosed mole (fig. 7). On the contrary, in fishes, very few LoF mutations were found in each pseudogene (fig. 7), the maximum being five LoF mutations in a pseudogene of *S. anshuiensis*. This comparison strongly supports the hypothesis that some vision genes of fossorial mammals have been under completely relaxed purifying selection for a much longer period of time than any cavefish vision genes.

## Discussion

When selection for maintaining a functional protein is completely relaxed, theory predicts that LoF mutations in coding and regulatory sequences can reach fixation by random genetic drift (Lynch and Conery 2000; Lahti et al. 2009). In an isolated population, among a set of dispensable genes, the longer the period of neutral evolution, the higher the expected number of pseudogenes, each carrying at least one LoF mutation. Eventually, all the genes under relaxed selection will be pseudogenized. At the level of a single gene, the longer the period of neutral evolution, the higher the expected number of LoF mutations. Thus, after a very long period of neutral evolution, all the neutrally evolving genes will carry many LoF mutations. The pace of this gene decay depends essentially on the pace of the appearance of LoF mutations (Li and Nei 1977). In the present study, we focused on a subset of LoF mutations that could be readily detected in genomes, that is, mutations that generate premature STOP codons, eliminate START or STOP codons, or disrupt intron splice sites, as well as small insertions/deletions (indels) causing translation frameshifts. Although this approach inevitably leads to an underestimation of the number of nonfunctional genes, it allowed us to carry out comparative studies and molecular dating of the relaxation of purifying selection. We analyzed molecular decay among three sets of genes in relation to the level of regression of three traits: eyes, circadian clock, and pigmentation. For most genes, assigning a gene to a category was straightforward, yet for some genes, it was more ambiguous. Vision genes corresponded primarily to a set of genes expressed only in eyes, however, fishes also express many nonvisual opsins genes that we assigned to this category on the basis of their homology to visual opsins and because most of them are likely just as dispensable in the absence of light as most eye-specific genes. Indeed, a similar pattern of gene decay was observed in these two gene sets and they were pooled for the quantitative analyses of relaxed selection and dating. Genes known for being involved in the circadian clock were assigned to a second set of genes. Some nonvisual opsins are involved in this process. Pigmentation genes comprised a large set of genes involved in several processes from pigment cell differentiation to pigment synthesis. Our a priori hypothesis was that vision genes should be more prone to degeneration in blind fishes as they are only expressed in eyes or involved in light sensing in various tissues, whereas many circadian clock and pigmentation genes may be maintained as their expression is not always restricted to regressed structures and functions. In accordance with this assumption, many pseudogenes were found among vision genes of the oldest blind cavefish, *L. dentata*, but only few pseudogenes were found among circadian clock and pigmentation genes in cavefishes.

Below, we discuss the repeated loss of a few genes involved in circadian clock and pigmentation. Then, we show how the loss of many vision genes in *L. dentata* sheds new light on gene decay in relation to eye regression in cavefishes. At a broader phylogenetic scale, we examine the contrasting vision gene decay in cavefishes and fossorial mammals.

### Molecular Evidence of Circadian Clock Disruption in Several Cavefishes

No LoF mutations were found in the set of circadian clock genes of both *Lucifuga* species. However, the nonvisual opsin *tmt3a* is pseudogenized in *L. gibarensis* and the loss of this gene is involved in the disruption of the circadian clock in the Somalian cavefish *Ph. andruzzii* (Cavallari et al. 2011). Thus, our survey of LoF mutations in Cuban cave brotulas suggests the loss of the circadian clock in *L. gibarensis*, but not in *L. dentata*. The maintenance of purifying selection on most circadian clock genes in both species is further supported by the analysis of nonsynonymous mutations showing similar accumulation of deleterious mutation in these species and in surface fishes. As expected, no LoF mutations in both ohnologs of circadian clock genes and nonvisual opsin genes were found in *S. grahami*, which is a surface fish. On the other hand, the small-eyed *S. rhinocerous* has accumulated more LoF mutations in both ohnologs (*per2*, *cry-dash*, *cry1b*, *cry2a*) than the blind *S. anshuiensis* (*cry-dash*). Interestingly, in both *Lucifuga* and *Sinocyclocheilus*, the molecular decay of the circadian clock is not correlated with the level of eye regression as the small-eyed species (*L. gibarensis* and *S. rhinocerous*) carry more pseudogenes than the blind species (*L. dentata* and *S. anshuiensis*).

LoF mutations were found repeatedly in a very small number of circadian clock genes, some of them already known to be involved in circadian clock disruption in other species. It suggests that only a small subset of genes may be involved in circadian clock loss, in particular those belonging to the cryptochromes and period families, which are light-inducible genes.

### A Small Set of Pigmentation Pseudogenes in Cavefishes

A similar trend was observed in the large set of pigmentation genes. Independent LoF mutations were found in *myo7ab* and *smtla* of *L. dentata* and *L. gibarensis* and both ohnologs of *gch2* and *pmelb* carried independent LoF mutations in *S. anshuiensis* and *S. rhinocerous*. Recurrent pseudogenization of only a couple of genes suggests that few pigmentation genes can be lost in cavefishes. They could belong to a very small subset of genes that may be involved in the depigmented phenotype without having additional and strong deleterious side effects.

### Many Pseudogenes among Cavefish Vision Genes

In sharp contrast with the very few pseudogenes found among circadian clock and pigmentation genes, many vision genes are pseudogenized in cavefishes. Before the present study, there was no evidence of decay in large numbers of both eye-specific genes and nonvisual opsin genes in a blind cavefish. Despite some cave populations belonging to *A. mexicanus* were assumed to be very ancient, that is several millions of years old, no LoF mutations were found in eye-specific genes of these fish with highly degenerate eyes (McGaugh et al. 2014). To explain this, very unlikely processes were assumed, such as “hidden functions” for these genes expressed only in eyes, and/or a high gene flow from the surface preventing the fixation of pseudogenes. But a high gene flow implies strong selection at other loci to maintain blindness in very small cavefish populations. However, these unlikely hypotheses are not necessary if we assume that *A. mexicanus* cave populations are very recent (Fumey et al. 2018) and that there has simply been not enough time for the appearance of many vision pseudogenes.

Rapid and extreme eye degeneration without eye-specific gene losses further questions the nature of the developmental mechanisms involved in eye loss, the pace of eye degeneration, and the correlation of eye degeneration with eye-specific gene decay in cavefishes. A clear refutation of the hypothesis that eye-specific genes and nonvisual opsin genes are under purifying selection in blind cavefishes was provided by the analysis of the genome of *L. dentata*, as 25% of these genes carry LoF mutations in these species. Moreover, the distribution of LoF among genes was consistent with the neutral evolution of most, if not all, vision genes in this species. The dispensability of most vision genes was further supported by the fact that other vision genes were identified with LoF mutations in other cavefishes. We predict that with more blind cavefish genomes becoming available we will find that most of these genes have been lost in at least one species. On the other hand, in *L. gibarensis*, which has small but functional eyes, most vision genes seemed under purifying selection, yet the partial degeneration of its visual system was correlated with the loss of several genes that were well conserved in eyed fishes. These data allowed us to propose a two-step scenario for the release of purifying selection on vision genes in *Lucifuga*. The last common ancestor of *L. dentata* and *L. gibarensis* was a cavefish that had accumulated a small number of pseudogenes in relation to life in darkness, which were not among the eye-specific genes studied here. In the lineage leading to *L. gibarensis*, only a few vision genes have been under relaxed selection whereas in the lineage leading to *L. dentata*, purifying selection has been relaxed on most vision genes. The small-eyed *L. gibarensis* may not be an intermediary stage to blindness as observed in *L. dentata*, but another stable state. Small-eyes and eyeless phenotypes could correspond to different ecological parameters such as different exposures to light. In accordance with this hypothesis, *L. gibarensis* belongs to an ancient monophyletic clade of small-eyed species living in Cuba and Bahamas (García-Machado et al. 2011; Hernández et al. 2020).

The lack of correlation between the degree of eye regression and the number of eye-specific pseudogenes suggests that the extent of eye regression should not be taken as a proxy of the evolutionary age of cavefish populations or species.

### Dating *L. dentata* Blindness

Although phylogenetic evidence suggested an ancient diversification in the cavefish genus *Lucifuga* and a monophyletic clade of distantly related blind species (García-Machado et al. 2011), there was no estimate of the time since this phenotype evolved. Similar estimates were obtained using several well-established methods and new approaches. With three methods relying on the shift of *ω* from a value lower than one (a signature of purifying selection) to one (a signature of neutral evolution), we found that the time since purifying selection was completely released on *L. dentata* vision genes is between 1.3 and 1.5 Ma. Using the number of pseudogenes in the set of vision genes, we estimated that *L. dentata* settled in caves ∼370,000 generations ago. The generation time of this fish is unknown, and translating the number of generations into years is difficult. However, assuming that the generation time is ∼4 years, which is realistic if we consider that these fish have a long lifespan and could reproduce for ∼10 years, the above independent estimates of relaxed selection would be compatible. They suggest that the loss of vision in the lineage leading to *L. dentata* occurred in the middle of the Pleistocene.

### Pattern of LoF Mutations in Tetraploids Cavefishes with Different Levels of Eye Regression: The Case of *Sinocyclocheilus*

The genus *Sinocyclocheilus*, which is endemic to southwestern karst areas in China, is the largest cavefish genus known to date (Xiao et al. 2005). In a genome-wide analysis, LoF mutations were found in many genes of three species, one species (*S. anshuiensis*) being blind and depigmented, another species (*S. rhinocerous*) having small eyes and being pigmented, and the last one (*S. grahami*) showing no cave-related traits (Yang et al. 2016). These species share a recent WGD with other cyprinids such as the common carp *Cyprinus carpio* (David et al. 2003; Yuan et al. 2010) which can explain why even the surface fish *S. grahami* carries many LoF mutations in vision, circadian clock, and pigmentation genes (Yang et al. 2016). However, no thorough comparisons were performed to relate differences in gene decay with the level of eye regression. We found that the number of vision pseudogenes in the blind *S. anshuiensis* is much higher than in the small-eyed *S. rhinocerous* and the eyed *S. grahami*, a result supporting the cumulative effect of tetraploidy and cave settlement on the rate of pseudogenization. As most genes are present twice, a gene function is most likely lost if, and only if, at least one LoF mutation is present in each ohnolog. With this criterion, seven vision genes were lost in *S. anshuiensis*, but only one in *S. rhinocerous* and *S. grahami*, indicating that selection maintaining functional vision genes is weaker in the blind species than in the two-eyed species. This conclusion was further supported by estimations of the strength of purifying selection on nonsynonymous mutations in vision genes, which showed that it was higher in the fish with large eyes.

### Contrasting Dynamics of Pseudogenization in Fossorial Mammals and Cavefishes

An extensive study of LoF mutations in genes coding for proteins involved in retinal networks using the genomes of three independently evolved fossorial mammals has been previously reported (Emerling and Springer 2014). All three species have functional eyes, but star-nosed moles often leave their burrows and thus have a greater exposure to light than naked mole-rats and Cape golden moles, which are entirely subterranean. The eyes of Cape golden moles are subcutaneous. More pseudogenes were identified in the Cape golden mole than in the naked mole-rat genome and the lowest number of pseudogenes was found in the star-nosed mole genome, suggesting that the decrease in retinal exposure to light allowed the decay of more vision genes. A striking difference between these fossorial mammals and the cavefishes studied here was that several pseudogenes of fossorial mammals carried a large number of LoF mutations, whereas cavefish pseudogenes accumulated at most five LoF mutations. This is in accordance with the adaptation of the fossorial mammals to the subterranean environment in the Oligocene, ∼25 Ma (Emerling and Springer 2014), whereas colonization of the dark environment by the cavefishes occurred much later, in the Pleistocene.

## Conclusion

Our analyses suggest that blind cavefishes for which genomes are available are not very ancient and that they all lost their eyes during the Pleistocene. The oldest, the Cuban *L. dentata*, in the middle of the Pleistocene and the most recent, the Mexican *A. mexicanus*, during the late Pleistocene or even later in the Holocene. Two gene-centered studies, based on molecular evolution of one and two genes, respectively, suggested that other blind cavefishes could be much older: some North American amblyopsid cavefishes may have accumulated LoF mutations over the last 10.3 My (Niemiller et al. 2013) and the Somalian cavefish *Ph. andruzzii* was estimated to be 5.3 My old (Calderoni et al. 2016). These estimations were based on very small numbers of nonsynonymous mutations and very few LoF mutations. Genome-wide analyses using several independent approaches are necessary to confirm that these cavefishes evolved much earlier than the cavefishes we analyzed here. If all cavefishes are actually relatively recent, the sequencing of a large number of blind cavefish genomes will be necessary to identify the whole set of eye-specific genes that are dispensable when eyes are highly degenerated, and the small subset of genes that has been repeatedly involved in the circadian clock disruption and depigmentation. Finding a blind cavefish genome in which most vision genes are pseudogenized and carry many LoF mutations would refute our current working hypothesis that blind cavefishes cannot thrive more than a few million years in cave ecosystems.

## Materials and Methods

### Genomic Resources for Two Cuban Cave Brotulas

A draft genome and a transcriptome were obtained for a specimen belonging to *L. dentata*. The reads of a specimen belonging to a closely related species, *L. gibarensis*, were mapped onto the *L. dentata* genome. A detailed description of the materials, methods, and results are given in supplementary data S1, Supplementary Material online. In the present study, these genomic resources were used to retrieve exon sequences and intron splice sites. The transcriptome allowed us to assess the reliability of the LoF mutations found in the genome of *L. dentata*.

### Vision, Circadian Clock, and Pigmentation Gene Sets

The set of vision genes included all opsins, visual opsins that are expressed in eye photoreceptor cells (cone and rods) but also nonvisual opsins that are expressed in various tissues. It also comprised eye-specific crystallin genes that were selected using expression patterns reported in zebrafish from the ZFINdatabase (https://zfin.org/) and in *A. mexicanus* (Hinaux et al. 2015). However, *crygm2* crystallin genes were excluded from the analyses. Indeed, many copies were found in fish genomes (>50 copies in *A. mexicanus*) and relaxed purifying selection on some copies could have occurred independently in response to any environmental shift. The set of vision genes also included genes coding for proteins involved in the phototransduction cascade and whose expression was restricted to the retina and/or the pineal complex: RPE65, Arrestins, Recoverins, Transducins, PDE6, CNGA3 and CNGB3, GCAPs, zGCs, and GRKs. Sets of circadian clock and pigmentation genes were defined on the basis of gene lists established in previous studies (Li et al. 2013; Lorin et al. 2018). The set of circadian clock genes was completed with *ck1δa* and *ck1δb* genes which are specific kinases of *cry* and *per* genes (Takahashi et al. 2008) and *aanat1* and *aanat2* genes whose expressions are regulated by the circadian clock in zebrafish (Vatine et al. 2011). The complete list of genes with their standardized identifiers is given in figure 1.

### Construction of Data Sets

The sequences of visual and nonvisual opsins of zebrafish were retrieved from (Davies et al. 2015). Other vision genes, circadian clock, and pigmentation genes of zebrafish were retrieved from GenBank.

A series of BlastN and TBlastX (Altschul et al. 1990) with zebrafish sequences were performed against *A. mexicanus* surface and Pachón cave genomes (GCF_000372685.2 and GCF_000372685.1, respectively), *S. grahami*, *S. rhinocerous*, *S. anshuiensis*, *P. nattereri*, *B. barbata*, *C. acus*, and *La. exutus* genomes (GCF_001515645.1, GCF_001515625.1, GCF_001515605.1, GCF_001682695.1, GCA_900303265.1, GCA_900312935.1, and GCA_900312555.1, respectively), and *L. dentata* and *L. gibarensis* genomes (this study). Matching regions were extracted using samtools (Li 2011) and CDS were predicted using EXONERATE with protein sequences of zebrafish (Slater and Birney 2005).

Given the low quality of the *A. mexicanus* cavefish genome assembly compared with the surface one (see in Results for an assessment of the quality of the *A. mexicanus* cavefish genome assembly), in order to analyze reliable gene sequences, cavefish reads were retrieved and mapped onto the genome of the surface fish using the NCBI remapping service with the default parameter values. This approach identified an opsin gene repertoire (36 genes) slightly larger than the one recently published (33 genes) using only the cavefish genome (Simon et al. 2019). Similarly, as a poor quality genome assembly was obtained for *L. gibarensis*, the reads were mapped on the high-quality *L. dentata* genome.

Incomplete CDS could be assembly artifacts, so they were not further analyzed. In order to estimate the percentage of missing exons in the *A. mexicanus* cavefish genome that were assembly artifacts, primers were designed to PCR amplify these exons in a cavefish using homologous sequences identified in the *A. mexicanus* surface fish genome.

For comparisons of the strength of purifying selection (*ω*) in surface fishes and cavefishes, orthologous CDS from well-annotated fish genomes—tetraodon (*D. nigroviridis*), cod (*G. morhua*), stickleback (*Gas. aculeatus*), spotted gar (*Le. oculatus*), tilapia (*Oreochromis niloticus*), medaka (*Ory. latipes*), platyfish (*X. maculatus*)—were downloaded from ENSEMBL 93, excepted visual opsin CDS which were retrieved from a study of their evolution in ray-finned fishes (Lin et al. 2017).

In order to compare the decay of vision genes in cavefishes and fossorial mammals, the number of pseudogenes, and the number of LoF mutations per pseudogene, in a set of genes coding for proteins involved in retinal networks were retrieved from a publication (Emerling and Springer 2014) for three fossorial mammals (the Cape golden mole *Ch. asiatica*, the naked mole-rat *H. glaber*, and star-nosed mole *Co. cristata*).

### Phylogenetic Analyses

Orthologous and paralogous relationships between genes were inferred through phylogenetic analyses. First, coding sequences were aligned using MUSCLE (Edgar 2004), after having taken into account indels (i.e., adding N where nucleotides were missing or removing additional nucleotides). For each alignment, DNA sequences were translated into protein sequences and a maximum likelihood phylogenetic tree was inferred using IQ-TREE (Nguyen et al. 2015) with the optimal model found by ModelFinder (Kalyaanamoorthy et al. 2017) and the robustness of the nodes was evaluated with 1,000 ultrafast bootstraps (Hoang et al. 2018). The trees were rooted and visualized using iTOL (Letunic and Bork 2007).

### Estimation of the Proportion of Neutral Genes Using the Distribution of LoF Mutations per Gene

In order to estimate the number of genes (*V*) under completely relaxed selection in a sample of (*T*) vision genes, we compared the observed distribution of LoF mutations per gene with the expected distribution taking into account that *V* genes can accumulate LoF mutations and *T*–*V* genes cannot carry LoF mutations. Assuming that an LoF mutation has a probability 1/*V* to be in a given gene among *V* genes, the probability that a gene contains *X* LoF mutations can be computed as follows:

$$\begin{array}{c} p\left(X=0\right)=\frac{V}{T}{\left(1-\frac{1}{V}\right)}^{m}+\frac{T-V}{T}\;\;\;\;\;\;\;\;\;\;\;\;\;\;\;\;\;\text{if}\;i=0\\ p\left(X=i\right)=\frac{V}{T}\frac{m!}{i!\left(m-i\right)!}{\left(\frac{1}{V}\right)}^{i}{\left(1-\frac{1}{V}\right)}^{m-i}\text{if}\;i\neq 0 \end{array},$$

where *m* is the total number of LoF mutations.

However, LoF mutations do not have the same probability of occurring in different genes if the size of the gene and the number of introns are variable. An LoF mutation is more likely in a gene with several large exons than in a gene with only one short exon. Simulations of the LoF mutation distribution were performed, taking into account the length of the coding sequence and the number of introns of each gene to estimate its relative mutation rate. We ran 10,000 simulations of the distribution of *m* mutations in a random sample of *V* genes taken at random among *T* vision genes. The distributions of the number of LoF mutations per gene in *L. dentata* and *L. gibarensis* were compared with expected distributions obtained with the two methods described above and for different values of *V*.

### Other Analyses of the Relaxation of Purifying Selection in Cavefishes

For each diploid species, gene sequences belonging to the same gene set (vision, circadian clock, or pigmentation) were concatenated. In order to analyze gene sequences of the tetraploid *Sinocyclocheilus* species, another alignment was produced. For each species and each gene set, gene sequences were concatenated after having taken at random one gene from each pair of ohnologs. This sampling process produced for each gene set two concatenated gene sequences for each species. With these six alignments of concatenated sequences, maximum likelihood estimates of *ω* were obtained using the program codeml from the PAML package Version 4.9h (Yang 2007). We considered three nested branch models: a “one-ratio” model assuming the same *ω* for all branches in the phylogeny; a “two-ratio” model assuming one *ω* for blind cavefishes (*ω*_CF_) and one *ω* for the other fishes (*ω*_SF_); a free-ratio model allowing a different *ω* for each branch. Likelihood ratio tests were performed to compare the likelihood values of the different models. Each likelihood ratio (LR) was calculated as twice the difference of log likelihood between the two models compared. The significance of the LR was determined by using the χ^2^ distribution with the number of degrees of freedom equal to the difference between the number of estimated parameters in the two models.

A further approach for detecting relaxed selection was carried out using the program RELAX (Wertheim et al. 2015), assigning the surface fishes as the reference. Each cavefish was independently assigned as the test branch. A value of the parameter *k* significantly different from one (*k* < 1 if selection is relaxed and *k* > 1 if selection is intensified) suggests a change in the selective regime in the cavefish lineage.

### Inferring the Deleterious Impact of Amino Acid Variants with MutPred2

Maximum likelihood inference of amino acids substitutions was performed using the program aaml from the PAML package Version 4.9h (Yang 2007). For each amino acid substitution, MutPred2 scores (Pejaver et al., unpublished data) and Grantham’s distances (Grantham 1974) were computed to estimate its deleterious impact.

In order to compare the distribution of scores (or distances) for a set of genes within a branch with the distribution expected under completely relaxed purifying selection, simulations of random mutations of nucleotides were generated, taking into account the length of the coding sequence of each gene and the transition/transversion ratio (https://github.com/MaximePolicarpo/Molecular-decay-of-light-processing-genes-in-cavefishes/blob/master/Neutral_evolution_for_mutpred.py).

### Dating Relaxation of Purifying Selection on Vision Genes in *L. dentata* Using the Number of Pseudogenes

In absence of purifying selection, the probability of fixation of an LoF mutation in a gene after *t* generations is:

$$p\left(t\right)=1-e^{-\mu_{\text{LoF}}t}\;\;\;\;\text{if}\;N_{e}\ll 1/\mu_{\text{LoF}},$$

where *µ*_LoF_ is the rate of LoF mutation per gene per generation and *N*_e_ is the effective population size (Li and Nei 1977).

For a set of *T* genes, assuming that each gene has the same rate of LoF mutation, the probability that *D* genes have fixed an LoF after *t* generations is:

$$p\left(X=D\right)=\frac{T!}{D!\left(T-D\right)!}{\left(1-e^{-\mu_{\mathrm{LoF}}t}\right)}^{D}{\left(e^{-\mu_{\mathrm{LoF}}t}\right)}^{T-D}.$$

The derivative of this function with respect to *t* allows to find for which value of *t* the probability $p\left(X=D\right)$ is maximal:

$$t=\frac{1}{\mu_{\mathrm{LoF}}}ln\left(\frac{T}{T-D}\right).$$

The estimation of *µ*_LoF_ taking into account the length of the CDS and the number of introns is described in supplementary data S3, Supplementary Material online.

For assessing the effect of the variation of *µ*_LoF_ among genes on dating, a program was written to simulate gene decay through accumulation of LoF mutations, taking into account the length and the number of introns to compute a *µ*_LoF_ for each gene (more details are given in supplementary data S3, Supplementary Material online).

### Other Methods for Dating Relaxation of Purifying Selection on Vision Genes in *L. dentata*

Vision genes from the two Cuban cave brotulas (*L. dentata* and *L. gibarensis*) and an outgroup (*B. barbata*) were concatenated and aligned. We assumed that vision genes have been under purifying selection along the branches of the phylogenetic tree, except in the lineage leading to *L. dentata* which is a mixed branch (with a period under purifying selection followed by a period under completely relaxed selection). The time since purifying selection was relaxed in this branch was estimated using two slightly different methods (Li et al. 1981; Meredith et al. 2009), and assuming that *B. barbata* and Cuban cave brotulas diverged 80 Ma (http://www.timetree.org/).

As an alternative approach, we used the distribution of MutPred2 scores in the lineage leading to *L. dentata*. First, we computed the proportions of two distributions, one under purifying selection as in the zebrafish lineage (*p*_s_) and one without selection as in simulated data (*p*_n_), that produce a mixture distribution that best fit the distribution of MutPred2 scores in the lineage leading to *L. dentata*. We assumed that *ω*_s_ under purifying selection shifted to *ω*_n_ when purifying selection was relaxed. We called *T*_d_ the time since the separation of *L. dentata* and *L. gibarensis*, *t*_s_ the period under purifying selection, and *t*_n_ the period under relaxed selection in the lineage leading to *L. dentata* (table 1). In this lineage, the proportion of nonsynonymous substitutions that accumulated under selection depends on *ω*_s_ and *t*_s_ and the proportion of nonsynonymous substitutions that accumulated under relaxed selection depends *ω*_n_ and *t*_n_. Thus, $\frac{p_{s}}{p_{n}}=\frac{t_{s}\omega_{s}}{t_{n}\omega_{n}}$ or $t_{n}=\frac{\omega_{s}}{\omega_{n}}\frac{p_{n}}{p_{s}}t_{s}$.

## Ethics approval

Animals were treated according to the French and European regulations for handling of animals in research.

## Supplementary Material

Supplementary data are available at *Molecular Biology and Evolution* online.

## Supplementary Material

msaa249_Supplementary_Data
