# Supplementary material for: Contrasting Gene Decay in Subterranean Vertebrates: Insights from Cavefishes and Fossorial Mammals
Source: Mol Biol Evol. 2020 Sep 28;38(2):589–605. doi: 10.1093/molbev/msaa249 (PMC7826195; doi:10.1093/molbev/msaa249)
Supplement: msaa249_Supplementary_Data [file msaa249_supplementary_data.zip › msaa249-suppl_data/Data S1 Lucifuga genomics.pdf]

Genomic resources for two Cuban cave brotulas
(*L. dentata and L. gibarensis*)

Materials and Methods

Assembly of *L. dentata* draft genome

The sequenced *L. dentata* specimen was a female, blind and depigmented (**supplementary fig. S1, Supplementary Material** online). DNA was extracted from muscle tissue using a protocol already described elsewhere (García-Machado, et al. 2011). Paired-end libraries were prepared with different insert sizes: 200 bp, 400 bp and 750 bp. A mate-pair library was also prepared with insert size in the range 3-5 kb. These libraries were sequenced on an Illumina HiSeq 2000 sequencer. After cleaning steps (adaptors trimming and quality trimming with Cutadapt V1.3 (Martin 2011)), assembly of a draft genome was performed using Minia (Chikhi and Rizk 2013) on all data, resulting in 662,154 contigs. After assembling, and as Minia doesn't use the paired-end information, scaffolding steps were performed using SSPACE (Boetzer, et al. 2011) on one library at a time in ascending order of insert size. The number of scaffolds decreased from 662,154 to 161,599 with the first library (insert size of 200 bp), to finish with 48,241 scaffolds with the mate pair library. This result was corrected by REAPR (Hunt, et al. 2013) to obtain 52,944 scaffolds. The remaining gaps were filled by GapCloser (Luo, et al. 2012).
The quality and completeness of the draft genome of *L. dentata* were assessed by remapping paired-end reads to the assembly using BWA v0.7.11 (Li and Durbin 2009) and BUSCO (Kriventseva, et al. 2015) with the Actinopterygii dataset comprising a total of 4,584 conserved genes.
The latter analysis was performed also on published draft genomes of three other Ophidiiformes in the family Ophidiidae (*Brotula barbata*, *Carapus acus* and *Lamprogrammus exutus*).

Assembly of *L. dentata* transcriptome

From the same individual, gonads, gills, heart and brain were dissected and stored in RNA-Later (Ambion). Total RNA isolation (using Trizol) led to yields of 870 ng/µl in gonads (RIN: 7.9), 750 ng/µl in gills (RIN: 8.6), 240 ng/µl in heart (RIN: 8.1) and 390 ng/µl in brain (RIN: 6). RNA from gonads, gills and heart were mixed in equal proportions to construct the first library. RNA from the brain was used to construct the second library.
For library preparation, polyA + RNA were extracted, fragmented, and directional libraries were prepared using the Small RNA Sample Prep Kit (Illumina). Both libraries were sequenced on an Illumina NextSeq500, on a Paired-end 2x150 bp run, using the High Output Kit 300 cycles sequencing kit. After cleaning steps (adaptors trimming and quality trimming with Cutadapt V1.3 (Martin 2011)), a *de novo* transcriptome assembly was obtained using Trinity and a quality assessment was realized following Trinity recommendations (https://github.com/trinityrnaseq/trinityrnaseq/wiki/Transcriptome-Assembly-Quality-Assessment).

Annotation of *L. dentata* draft genome

First, repetitive elements were identified using RepeatMasker v4.0.7 (Smit, et al. 2013), Dust (Morgulis, et al. 2006) and TRF v4.09 (Benson 1999). A species specific *de novo* repeat library

was built with RepeatModeler v1.0.11 (Smit and Hubley 2008) and repeated regions were located using RepeatMasker with the *de novo* and *Danio rerio* libraries. Bedtools v2.26.0 (Quinlan and Hall 2010) were used to merge repeated regions identified with the three tools and to soft masked the genome. Then, MAKER3 genome annotation pipeline v3.01.02-beta (Holt and Yandell 2011) combined annotations and evidence from three approaches: similarity with fish proteins, assembled transcripts and *de novo* gene predictions. Protein sequences from 11 other fish species (*Astyanax mexicanus*, *Danio rerio*, *Gadus morhua*, *Gasterosteus aculeatus*, *Lepisosteus oculatus*, *Oreochromis niloticus*, *Oryzias latipes*, *Poecilia formosa*, *Takifugu rubripes*, *Dichotomyctere nigroviridis*, *Xiphophorus maculatus*) found in Ensembl were aligned to the masked genome using Exonerate v2.4 (Slater and Birney 2005). RNA-Seq reads were mapped to the genome assembly using STAR v2.5.1b (Dobin, et al. 2013) with outWigType and outWigStrand options to output signal wiggle files. Cufflinks v2.2.1 (Trapnell, et al. 2010) was used to assemble the transcripts which were used as RNA-seq evidence. A *de novo* gene model was built using Braker v2.0.4 (Hoff, et al. 2016) with wiggle files provided by STAR as hints file for GeneMark and Augustus trainings. The best supported transcript for each gene was chosen using the quality metric called Annotation Edit Distance (AED) (Eilbeck, et al. 2009). The annotation completeness of coding genes was assessed by BUSCO v3 using the actinopterygii_odb9 gene set. Homology to uniprot database was used to infer functions of predicted genes with Blastp and an e-value cutoff of $1e^{-6}$. Interproscan 5.35 (Jones, et al. 2014) was used to detect proteins with known functional domains.

Analysis of repeated elements

The *de novo* library of repeated elements was refined with the following procedure: removal of short (<80 bp) consensus repeats; reannotation of satellite sequences as well as of putative DNA or LTR transposable elements (TEs) by aligning each consensus against itself (this procedure allows to visualize internal repeats); Blastn (Altschul, et al. 1990) of the library against itself and removal of redundant TEs; Blastx (Altschul, et al. 1990) of « Unknown » repeats against the NCBI protein database and removal of multigene families erroneously identified as putative TEs; reannotation of putative SINEs according to the SINE-scan program (Mao and Wang 2017). Finally, the library was manually curated: consensus sequences were compared to an in-house library of transposable element proteins using BlastX. Matching Unknown elements were renamed according to their hits against this library. Consensus sequences showing incongruent annotations between RepeatModeler automatic classification and our manual annotation were further submitted to Censor (Kohany, et al. 2006). This TE library was used as the repeat database for a RepeatMasker search in the genome (Smit, et al. 2013). Overlaps in RepeatMasker output were discarded by selecting highest scoring elements. Repeat fragments closer than 20 bp and having the same name were merged. The landscape was reconstructed from RepeatMasker align output using the calcDivergenceFromAlign.pl and createRepeatLandscape.pl utilities of the RepeatMasker suite.

Mapping of *L. gibarensis* sequences on *L. dentata* draft genome

The sequenced *L. gibarensis* specimen was a male, had small eyes and was pigmented (**supplementary fig. S1, Supplementary Material** online). One mate-pair library was prepared, which had inserts size between 3 kb and 10 kb. The library was sequenced on an Illumina NextSeq sequencer. Sequences from *L. gibarensis* were mapped on the draft genome of *L. dentata* using BWA v0.7.11.

Results

Assembly of *L. dentata* draft genome

Assembly resulted in 52,944 scaffolds whose size sum up to 634 Mb, N50 = 119.6 kb (for scaffold size distribution (**fig. 1**). This genome size is consistent with the size of three other genomes of Ophidiiformes available - *Brotula barbata*, *Lamprogrammus exutus* and *Carapus acus* - (Malmstrøm, et al. 2017) and estimates of the genome size of five other Ophidiiformes (Gregory 2019). To assess the quality of the assembly, raw sequences were realigned to the assembly: 95% of the reads realigned correctly resulting in a mean coverage of 134x.

Genome assembly completeness

It was assessed using BUSCO with the Actinopterygii gene database (Kriventseva, et al. 2015). Among 4,584 genes, 4,249 (92.7%) were found complete, 194 (4.2%) were incomplete and 141 (3.1%) were missing. Using BUSCO with three other Ophidiiform genomes currently available (*Brotula barbata*, *Carapus acus* and *Lamprogrammus exutus*), the genome of *Lucifuga dentata* appeared as the most complete (**fig. 2**)

Assembly of a transcriptome of *L. dentata* and genome annotation

Based on mRNA extracted from the gonads, gills, heart and brain of *L. dentata*, a *de novo* transcriptome assembly was obtained using Trinity (Grabherr, et al. 2011). Among 4,584 genes corresponding to the Actinopterygii gene database of BUSCO, 82.8% were found complete (**fig. 2**), 92.31 % of the reads were mapped back to the assembly and 84 % of the reads were properly paired, which indicate an overall good quality transcriptome. More on quality check can be found in **fig. 3)**.

A combination of *de novo* predictions, RNA-seq evidence and protein alignments was used to annotate the genome of *L. dentata* (**fig. 4**). This resulted in 30,001 gene models with an average gene length of 9,693 bp and an average protein length of 435 amino acids. Among predicted genes, 23,524 had a functional annotation with BLAST to the SwissProt/UniProt database and 21,558 genes were detected with a functional domain by Interproscan. Annotation completeness was assessed using BUSCO in protein mode; among 4,584 corresponding to the Actinopterygii gene database of BUSCO, 87.4% were found complete, 6.5% incomplete and 6% missing (**fig. 2**). A homemade pipeline was used to describe the repeat landscape of the genome of *L. dentata*: 16.3% was repetitive elements, with 2.4% of the genome being LINEs and 0.4% of SINEs (**fig. 5**).

Mapping of *L. gibarensis* sequences on *L. dentata* draft genome

As nuclear DNA sequence divergence is about 1% between the two *Lucifuga* species, short reads of *L. gibarensis* could be mapped on *L. dentata* draft genome. The mean coverage was 84x, with 86% of the reads mapping on the genome.

References

Altschul SF, Gish W, Miller W, Myers EW, Lipman DJ. 1990. Basic local alignment search tool. J Mol Biol 215:403-410.

Benson G. 1999. Tandem repeats finder: a program to analyze DNA sequences. Nucleic Acids Research 27:573-580.
Boetzer M, Henkel CV, Jansen HJ, Butler D, Pirovano W. 2011. Scaffolding pre-assembled contigs using SSPACE. Bioinformatics 27:578-579.
Chikhi R, Rizk G. 2013. Space-efficient and exact de Bruijn graph representation based on a Bloom filter. Algorithms for Molecular Biology 8:22.
Dobin A, Davis CA, Schlesinger F, Drenkow J, Zaleski C, Jha S, Batut P, Chaisson M, Gingeras TR. 2013. STAR: ultrafast universal RNA-seq aligner. Bioinformatics 29:15-21.
Eilbeck K, Moore B, Holt C, Yandell M. 2009. Quantitative measures for the management and comparison of annotated genomes. BMC Bioinformatics 10:67.
García-Machado E, Hernandez D, Garcia-Debras A, Chevalier-Monteagudo P, Metcalfe C, Bernatchez L, Casane D. 2011. Molecular phylogeny and phylogeography of the Cuban cave-fishes of the genus *Lucifuga*: evidence for cryptic allopatric diversity. Mol Phylogenet Evol 61:470-483.
Grabherr MG, Haas BJ, Yassour M, Levin JZ, Thompson DA, Amit I, Adiconis X, Fan L, Raychowdhury R, Zeng Q, et al. 2011. Full-length transcriptome assembly from RNA-Seq data without a reference genome. Nature Biotechnology 29:644.
Gregory TR. 2019. Animal Genome Size. http://www.genomesize.com.
Hoff KJ, Lange S, Lomsadze A, Borodovsky M, Stanke M. 2016. BRAKER1: Unsupervised RNA-Seq-Based Genome Annotation with GeneMark-ET and AUGUSTUS. Bioinformatics 32:767-769.
Holt C, Yandell M. 2011. MAKER2: an annotation pipeline and genome-database management tool for second-generation genome projects. BMC Bioinformatics 12:491.
Hunt M, Kikuchi T, Sanders M, Newbold C, Berriman M, Otto TD. 2013. REAPR: a universal tool for genome assembly evaluation. Genome Biology 14:R47.
Jones P, Binns D, Chang H-Y, Fraser M, Li W, McAnulla C, McWilliam H, Maslen J, Mitchell A, Nuka G, et al. 2014. InterProScan 5: genome-scale protein function classification. Bioinformatics 30:1236-1240.
Kohany O, Gentles AJ, Hankus L, Jurka J. 2006. Annotation, submission and screening of repetitive elements in Repbase: RepbaseSubmitter and Censor. BMC Bioinformatics 7:474.
Kriventseva EV, Zdobnov EM, Simão FA, Ioannidis P, Waterhouse RM. 2015. BUSCO: assessing genome assembly and annotation completeness with single-copy orthologs. Bioinformatics 31:3210-3212.
Li H, Durbin R. 2009. Fast and accurate short read alignment with Burrows-Wheeler transform. Bioinformatics 25:1754-1760.
Luo R, Liu B, Xie Y, Li Z, Huang W, Yuan J, He G, Chen Y, Pan Q, Liu Y, et al. 2012. SOAPdenovo2: an empirically improved memory-efficient short-read de novo assembler. GigaScience 1:18.
Malmstrøm M, Matschiner M, Tørresen OK, Jakobsen KS, Jentoft S. 2017. Whole genome sequencing data and de novo draft assemblies for 66 teleost species. Scientific data 4:160132.
Mao H, Wang H. 2017. SINE_scan: an efficient tool to discover short interspersed nuclear elements (SINEs) in large-scale genomic datasets. Bioinformatics 33:743-745.
Martin M. 2011. Cutadapt removes adapter sequences from high-throughput sequencing reads. EMBnet.journal 17:10-12.

Morgulis A, Gertz EM, Schäffer AA, Agarwala R. 2006. A Fast and Symmetric DUST Implementation to Mask Low-Complexity DNA Sequences. Journal of Computational Biology 13:1028-1040.
Quinlan AR, Hall IM. 2010. BEDTools: a flexible suite of utilities for comparing genomic features. Bioinformatics 26:841-842.
Slater GSC, Birney E. 2005. Automated generation of heuristics for biological sequence comparison. BMC Bioinformatics 6:31.
Smit AFA, Hubley R. 2008. RepeatModeler Open 1.0 . http://www.repeatmasker.org.
Smit AFA, Hubley R, Green P. 2013. RepeatMasker Open 4.0. http://www.repeatmasker.org.
Trapnell C, Williams BA, Pertea G, Mortazavi A, Kwan G, van Baren MJ, Salzberg SL, Wold BJ, Pachter L. 2010. Transcript assembly and quantification by RNA-Seq reveals unannotated transcripts and isoform switching during cell differentiation. Nature Biotechnology 28:511-515.

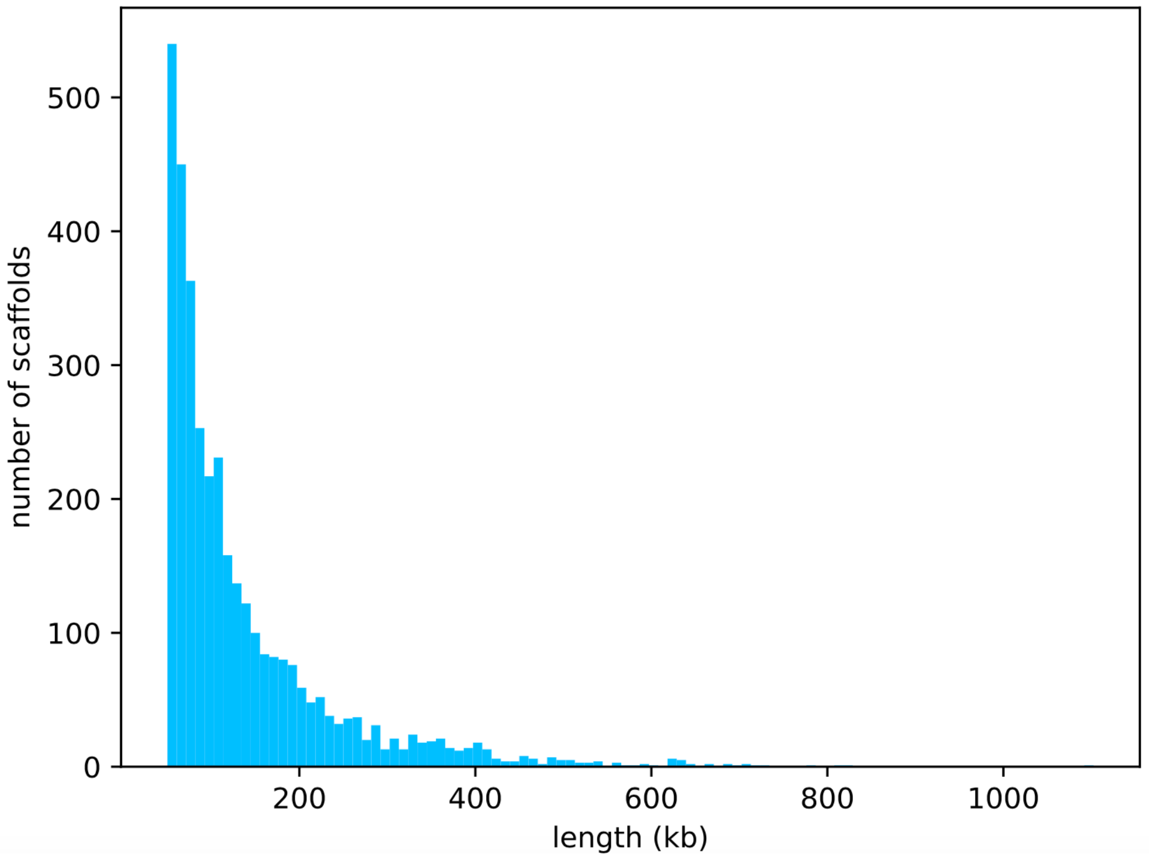

Figure 1. *L. dentata* scaffold size distribution. Based on 3,537 scaffolds longer than 50kb (49,407 scaffolds < 50 kb not used)

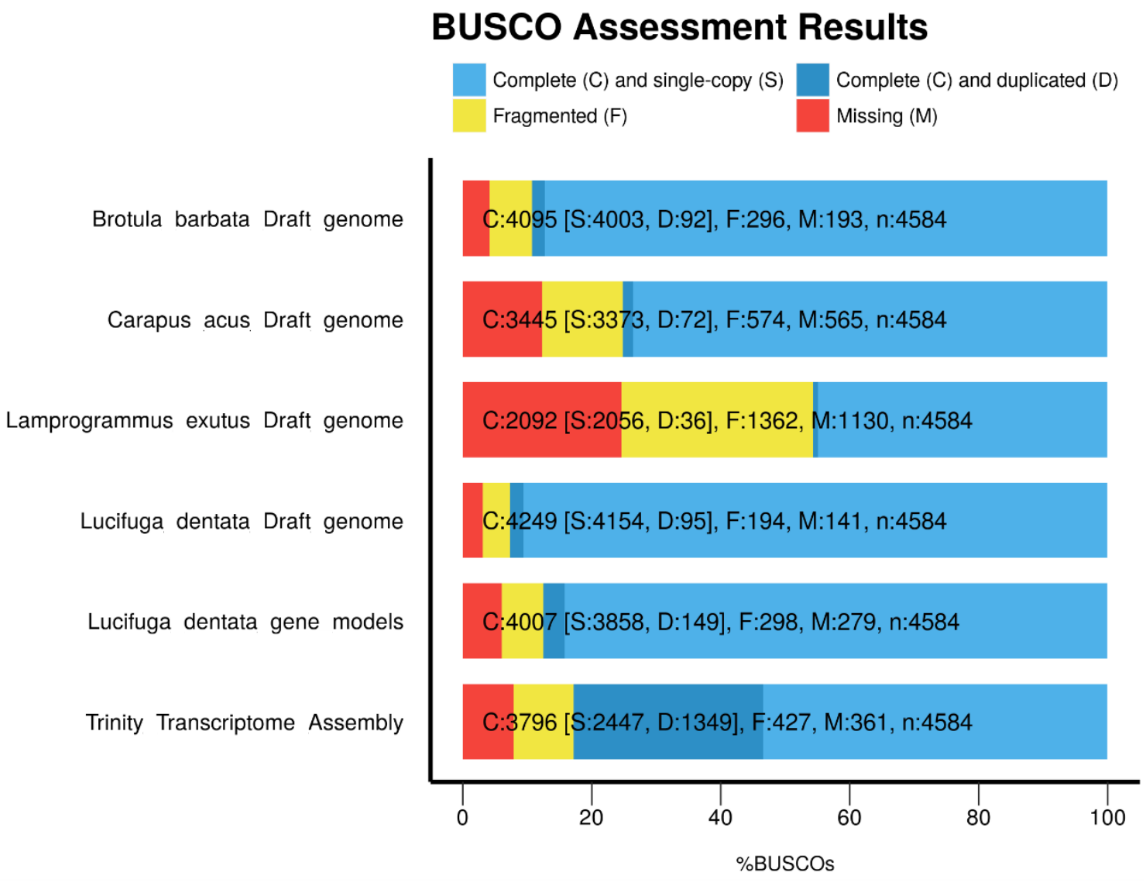

Figure 2. BUSCO analyses using the Actinopterygii gene database (v3.1.0). We assessed the completeness of three published Ophidiiformes genomes (*Brotula barbata*, *Carapus acus* and *Lamprogrammus exutus*), *Lucifuga dentata* genome, gene models resulting from the annotation pipeline and transcriptome assembly

```
######################### RNA Seq Read Representation by Trinity Assembly #########################

159178098 reads; of these:
  159178098 (100.00%) were paired; of these:
    25554370 (16.05%) aligned concordantly 0 times
    22283453 (14.00%) aligned concordantly exactly 1 time
    111340275 (69.95%) aligned concordantly >1 times
    ----
    25554370 pairs aligned concordantly 0 times; of these:
      1167404 (4.57%) aligned discordantly 1 time
    ----
    24386966 pairs aligned 0 times concordantly or discordantly; of these:
      48773932 mates make up the pairs; of these:
        24491684 (50.21%) aligned 0 times
        4741471 (9.72%) aligned exactly 1 time
        19540777 (40.06%) aligned >1 times
92.31% overall alignment rate



#########################  Counting Full Length Trinity Transcripts #########################


#hit_pct_cov_bin    count_in_bin    >bin_below
100    7644    7644
90    2304    9948
80    1841    11789
70    1691    13480
60    1825    15305
50    1939    17244
40    2169    19413
30    2260    21673
20    2103    23776
10    793    24569



#########################  Transcriptome Contig Nx Statistic #########################


################################
## Counts of transcripts, etc.
################################
Total trinity 'genes':    327313
Total trinity transcripts:    511116
Percent GC: 44.35

########################################
Stats based on ALL transcript contigs:
########################################
```

Contig N10: 4707
Contig N20: 3358
Contig N30: 2545
Contig N40: 1932
Contig N50: 1408

Median contig length: 368
Average contig: 752.13
Total assembled bases: 384426633

###
Stats based on ONLY LONGEST ISOFORM per 'GENE':
###

Contig N10: 3722
Contig N20: 2418
Contig N30: 1536
Contig N40: 944
Contig N50: 629

Median contig length: 310
Average contig: 525.20
Total assembled bases: 171905036

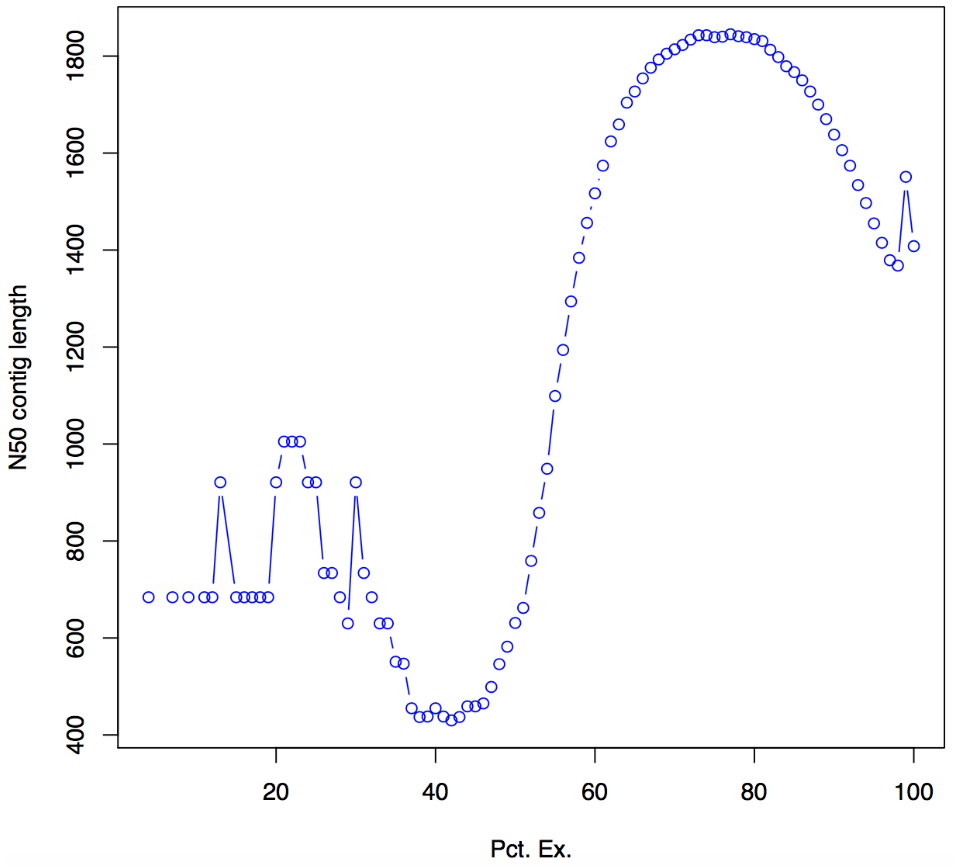

Figure 3. Transcriptome statistics. We followed the transcriptome assembly quality assessment of Trinity (https://github.com/trinityrnaseq/trinityrnaseq/wiki/Transcriptome-Assembly-Quality-Assessment).

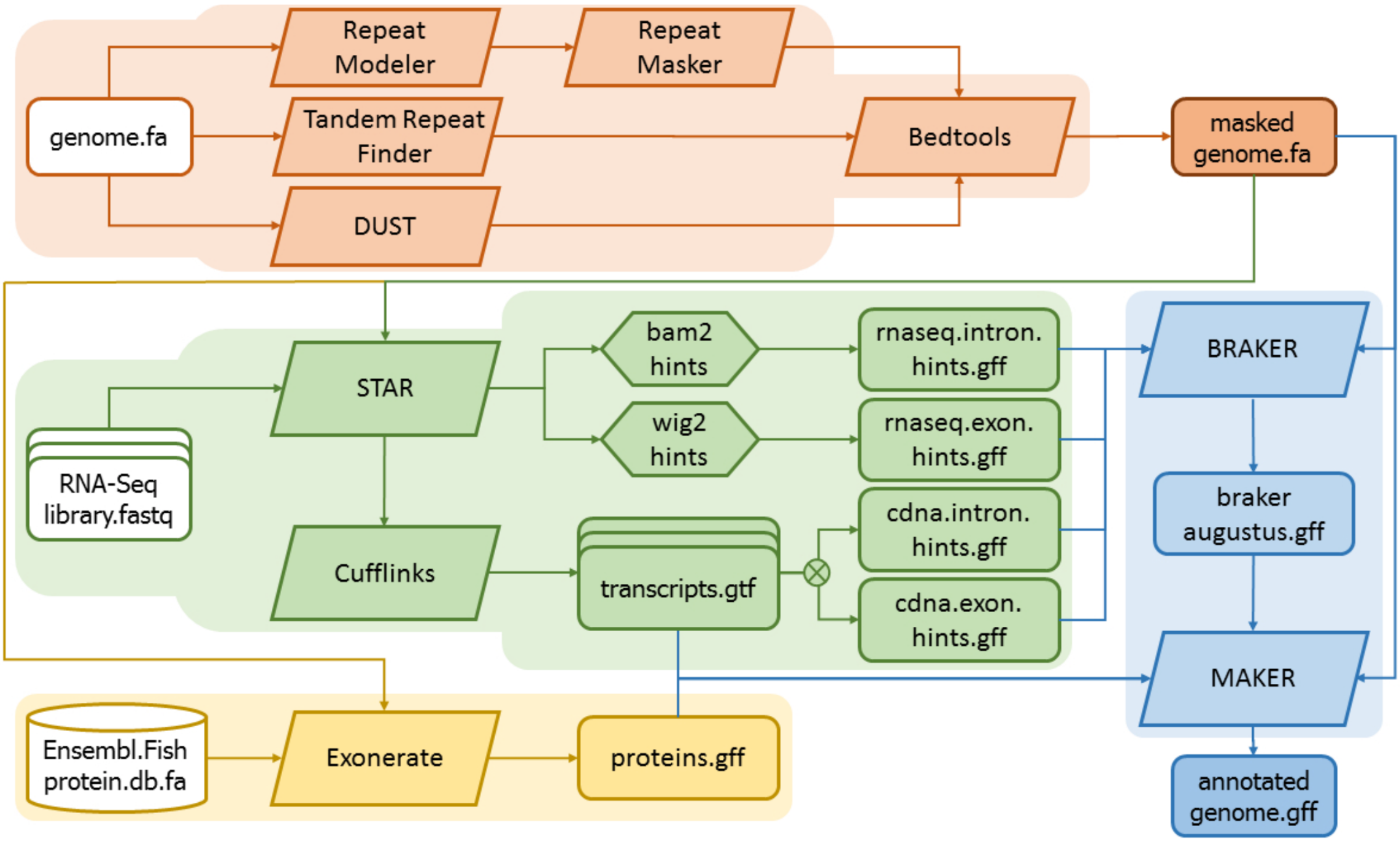

Figure 4. Genome annotation pipeline used on the *Lucifuga dentata* draft genome.

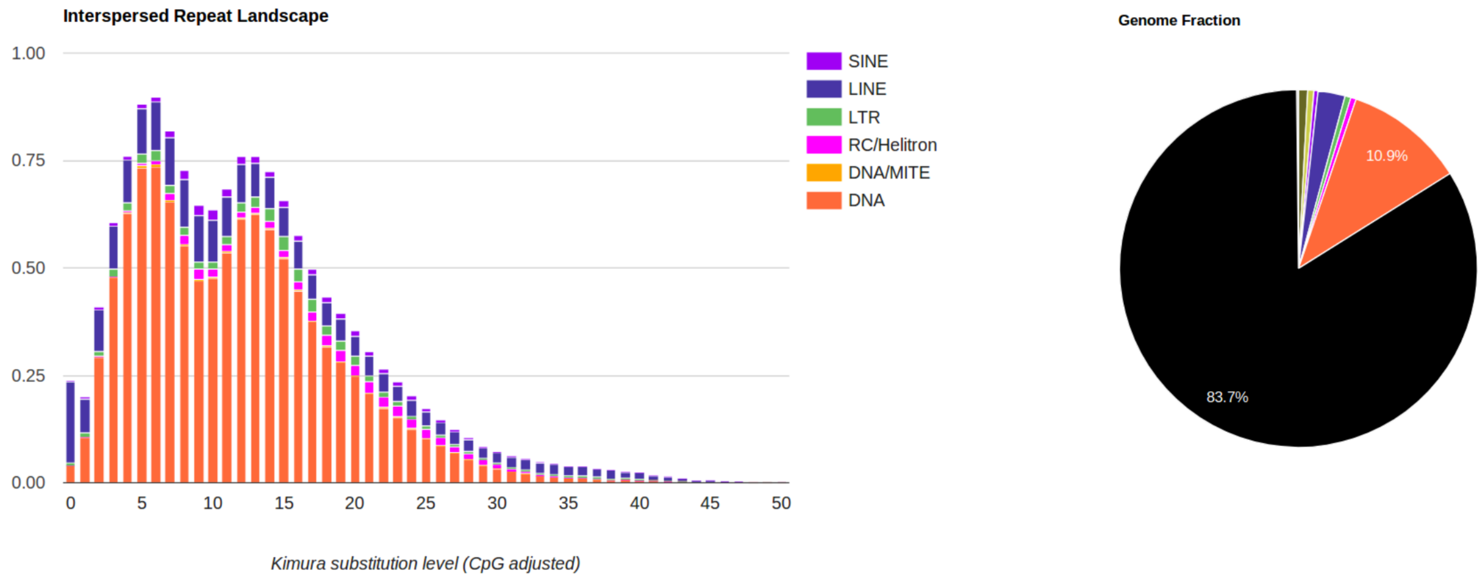

Figure 5. Interspersed repeat landscape of *Lucifuga dentata*.
