# Supplementary material for: Contrasting Gene Decay in Subterranean Vertebrates: Insights from Cavefishes and Fossorial Mammals
Source: Mol Biol Evol. 2020 Sep 28;38(2):589–605. doi: 10.1093/molbev/msaa249 (PMC7826195; doi:10.1093/molbev/msaa249)
Supplement: msaa249_Supplementary_Data [file msaa249_supplementary_data.zip › msaa249-suppl_data/Data S2 Putative impact of some LoF mutations.pdf]

Putative impact of some LoF mutations

Vision genes: in *L. dentata*, a frameshift was found in the alpha-crystallin, *cryaa*, whose downregulation in *A. mexicanus* cavefish plays a key role in triggering lens apoptosis (Ma, et al. 2014; Hinaux, et al. 2015). Another crystallin, *crybb1*, is pseudogenized in *L. dentata*. Mutations in this gene cause lens opacity in humans (Mackay, et al. 2002). We also found LoF mutations in two opsin receptor kinases, *grk7a* and *grk1b*. Mutations in these proteins can lead to overactive opsin and photoreceptor degeneration (Feng, et al. 2017). These two genes and *grk7b* have similar functions and are all expressed in cones. As these three kinases may have additive effects (Osawa and Weiss 2012), we can hypothesize that the absence or malfunction of one of them could be compensated by the others. Such compensation could explain why we found that both *grk7b* ohnologs carry LoF mutations in *S. grahami,* despite the fact that this fish has large eyes showing no evidence of degeneration. Another interesting gene is *gnb3b* which is pseudogenized in both *L. dentata* and *L. gibarensis* and which is linked to night-blindness in humans (Vincent, et al. 2016), yet *gnb3*$^{-/-}$ mice seem to have functional photoreceptors (Nikonov, et al. 2013). Finally, we found LoF mutations in *gcap2*, a guanylate cyclase activator, in both *Lucifuga* species. This gene is associated with retinitis pigmentosa in humans (Sato, et al. 2005) but it could be compensated by overexpression of *gcap1* in rods (Makino, et al. 2012). In *Astyanax mexicanus*, a deletion of 11 bp in the phosphodiesterase *pde6b,* a rod-expressed gene, leads to several STOP codons in the catalytic domain (Lagman, et al. 2016). Mutations in this gene were associated with night-blindness and retinitis pigmentosa in humans (McLaughlin, et al. 1993; Gal, et al. 1994). Moreover, in mice affected by mutations in the ortholog of *pde6b*, rod photoreceptors degenerate during development resulting in a total absence of photoreceptors in the adult (Farber and Lolley 1974; Chang, et al. 2002).
Most LoF mutations were found in the subset of non-visual opsins, which makes their functional impact difficult to evaluate as the functions of these genes are still poorly understood. Two notable exceptions are *opn4m2* and *tmt3a,* pseudogenized in *S. anshuiensis* and *L. gibarensis* respectively, and known to be non-functional and as such involved in the deregulation of the circadian clock in *P. andruzzii* (Cavallari, et al. 2011).

Circadian clock genes: in *S. rhinocerous,* both ohnologs of four circadian clock genes, *cry1b*, *cry2a*, p*er2* and *cry-dash,* carried LoF mutations. In *S. anshuiensis*, both ohnologs of *cry-dash* carried also LoF mutations which are independent of those found in *S. rhinocerous*. The gene *cry-dash,* involved in photoreactivation DNA repair, is also pseudogenized in *Phreatichthys andruzzii* (Zhao, et al. 2018) as well as *per2* that could be involved in the disruption of the circadian rhythm in this species (Ceinos, et al. 2018).

Pigmentation genes: both *L. dentata* (depigmented skin) and *L. gibarensis* (pigmented skin) carried independent LoF mutations in *myo7ab*. While no *myo7ab*-/- mutant has been analyzed, the paralog *myo7aa*-/- mutant in zebrafish showed an elevated photoreceptor death but the pigmentation was not affected (Wasfy, et al. 2014). Both *Lucifuga* species had independently fixed LoF mutations in *smtla* which is known to increase the number of leucophores at the expense of a reduced number of xanthophores in medaka (Fukamachi, et al. 2009). In *L. dentata, slc2a11b* is pseudogenized and this gene codes for a protein that promotes yellow pigmentation (Kimura, et al. 2014; Parichy and Spiewak 2015). Two other genes, *trpm1a* and *trpm1b* are also pseudogenized in *L. dentata*. During zebrafish development, *trpm1a* is expressed in the retina and melanophores whereas *trpm1b* expression is restricted to the retina (Kastenhuber, et al. 2013). In humans, mutations in their ortholog TRPM1 lead to complete congenital stationary night blindness (Audo, et al. 2009). In *L. gibarensis*, *pax7* which promotes

xanthophore differentiation (Nord, et al. 2016) carried a LoF mutation as well as *edn3b* that is known to lead to a reduction in iridophore numbers when mutated in zebrafish (Krauss, Frohnhöfer, et al. 2014). In *Astyanax mexicanus*, two pigmentation genes were found with LoF mutations: *mc1r* which carried a 2 bp deletion that could be involved in pigmentation reduction in two cave populations belonging to this species (Gross, et al. 2009) and *tyrp1a* which carried a 1 bp deletion. In zebrafish, morpholino-induced knock-down of *tyrp1a* had no phenotypic effect (Krauss, Geiger-Rudolph, et al. 2014). In *S. rhinocerous* (pigmented skin) and *S. anshuiensis* (depigmented skin), both ohnologs of *gch2* and *pmelb* carried independent LoF mutations. It has been shown that *gch2* mutant lacked proper xanthophore pigmentation at larval stages in zebrafish but no effect were reported in the adult (Parichy, et al. 2000; Pelletier, et al. 2001; Lister 2019). In the same way, injection of *pmelb* morpholinos in the zebrafish had no significant effect on the number of melanosomes but led to a significant loss of their cylindrical shape (Burgoyne, et al. 2015).

Many pigmentation pseudogenes seem to be compensated by their teleost-specific duplicates when lost in zebrafish, such as *tyrp1a* (Krauss, Geiger-Rudolph, et al. 2014), *pmelb* (Burgoyne, et al. 2015) and *pax7b* (Nord, et al. 2016).

References

Audo I, Kohl S, Leroy BP, Munier FL, Guillonneau X, Mohand-Saïd S, Bujakowska K, Nandrot EF, Lorenz B, Preising M, et al. 2009. TRPM1 is mutated in patients with autosomal-recessive complete congenital stationary night blindness. American Journal of Human Genetics 85:720-729.

Burgoyne T, Connor MN, Seabra MC, Cutler DF, Futter CE. 2015. Regulation of melanosome number, shape and movement in the zebrafish retinal pigment epithelium by OA1 and PMEL. Journal of Cell Science 128:1400-1407.

Cavallari N, Frigato E, Vallone D, Froehlich N, Fernando Lopez-Olmeda J, Foa A, Berti R, Javier Sanchez-Vazquez F, Bertolucci C, Foulkes NS. 2011. A Blind Circadian Clock in Cavefish Reveals that Opsins Mediate Peripheral Clock Photoreception. Plos Biology 9:e1001142.

Ceinos RM, Frigato E, Pagano C, Fröhlich N, Negrini P, Cavallari N, Vallone D, Fuselli S, Bertolucci C, Foulkes NS. 2018. Mutations in blind cavefish target the light-regulated circadian clock gene, period 2. Scientific Reports 8:8754.

Chang B, Hawes NL, Hurd RE, Davisson MT, Nusinowitz S, Heckenlively JR. 2002. Retinal degeneration mutants in the mouse. Vision Res 42:517-525.

Farber DB, Lolley RN. 1974. Cyclic Guanosine Monophosphate: Elevation in Degenerating Photoreceptor Cells of the C3H Mouse Retina. Science 186:449-451.

Feng D, Chen Z, Yang K, Miao S, Xu B, Kang Y, Xie H, Zhao C. 2017. The cytoplasmic tail of rhodopsin triggers rapid rod degeneration in kinesin-2 mutants. Journal of Biological Chemistry 292:17375-17386.

Fukamachi S, Yada T, Meyer A, Kinoshita M. 2009. Effects of constitutive expression of somatolactin alpha on skin pigmentation in medaka. Gene 442:81-87.

Gal A, Orth U, Baehr W, Schwinger E, Rosenberg T. 1994. Heterozygous missense mutation in the rod cGMP phosphodiesterase β–subunit gene in autosomal dominant stationary night blindness. Nature Genetics 7:64-68.

Gross JB, Borowsky R, Tabin CJ. 2009. A novel role for Mc1r in the parallel evolution of depigmentation in independent populations of the cavefish *Astyanax mexicanus*. PLoS Genet 5:e1000326.

Hinaux H, Blin M, Fumey J, Legendre L, Heuze A, Casane D, Retaux S. 2015. Lens Defects in *Astyanax mexicanus* Cavefish: Evolution of Crystallins and a Role for alphaA-Crystallin. Developmental Neurobiology 75:505-521.
Kastenhuber E, Gesemann M, Mickoleit M, Neuhauss SCF. 2013. Phylogenetic analysis and expression of zebrafish transient receptor potential melastatin family genes. Developmental Dynamics 242:1236-1249.
Kimura T, Nagao Y, Hashimoto H, Yamamoto-Shiraishi Y-i, Yamamoto S, Yabe T, Takada S, Kinoshita M, Kuroiwa A, Naruse K. 2014. Leucophores are similar to xanthophores in their specification and differentiation processes in medaka. Proceedings of the National Academy of Sciences of the United States of America 111:7343-7348.
Krauss J, Frohnhöfer HG, Walderich B, Maischein H-M, Weiler C, Irion U, Nüsslein-Volhard C. 2014. Endothelin signalling in iridophore development and stripe pattern formation of zebrafish. Biology Open 3:503-509.
Krauss J, Geiger-Rudolph S, Koch I, Nüsslein-Volhard C, Irion U. 2014. A dominant mutation in tyrp1A leads to melanophore death in zebrafish. Pigment Cell & Melanoma Research 27:827-830.
Lagman D, Franzén IE, Eggert J, Larhammar D, Abalo XM. 2016. Evolution and expression of the phosphodiesterase 6 genes unveils vertebrate novelty to control photosensitivity. Bmc Evolutionary Biology 16:124.
Lister JA. 2019. Larval but not adult xanthophore pigmentation in zebrafish requires GTP cyclohydrolase 2 (gch2) function. Pigment Cell & Melanoma Research 0.
Ma L, Parkhurst A, Jeffery W. 2014. The role of a lens survival pathway including sox2 and alphaA-crystallin in the evolution of cavefish eye degeneration. Evodevo 5:28.
Mackay DS, Boskovska OB, Knopf HLS, Lampi KJ, Shiels A. 2002. A Nonsense Mutation in CRYBB1 Associated with Autosomal Dominant Cataract Linked to Human Chromosome 22q. American Journal of Human Genetics 71:1216-1221.
Makino CL, Wen X-H, Olshevskaya EV, Peshenko IV, Savchenko AB, Dizhoor AM. 2012. Enzymatic Relay Mechanism Stimulates Cyclic GMP Synthesis in Rod Photoresponse: Biochemical and Physiological Study in Guanylyl Cyclase Activating Protein 1 Knockout Mice. PLoS ONE 7:e47637.
McLaughlin ME, Sandberg MA, Berson EL, Dryja TP. 1993. Recessive mutations in the gene encoding the β–subunit of rod phosphodiesterase in patients with retinitis pigmentosa. Nature Genetics 4:130-134.
Nikonov SS, Lyubarsky A, Fina ME, Nikonova ES, Sengupta A, Chinniah C, Ding X-Q, Smith RG, Pugh EN, Jr., Vardi N, et al. 2013. Cones respond to light in the absence of transducin β subunit. The Journal of neuroscience : the official journal of the Society for Neuroscience 33:5182-5194.
Nord H, Dennhag N, Muck J, von Hofsten J. 2016. Pax7 is required for establishment of the xanthophore lineage in zebrafish embryos. Molecular biology of the cell 27:1853-1862.
Osawa S, Weiss ER editors. Retinal Degenerative Diseases. 2012 Boston, MA.
Parichy DM, Ransom DG, Paw B, Zon LI, Johnson SL. 2000. An orthologue of the kit-related gene fms is required for development of neural crest-derived xanthophores and a subpopulation of adult melanocytes in the zebrafish, Danio rerio. Development 127:3031.
Parichy DM, Spiewak JE. 2015. Origins of adult pigmentation: diversity in pigment stem cell lineages and implications for pattern evolution. Pigment Cell & Melanoma Research 28:31-50.
Pelletier I, Bally-Cuif L, Ziegler I. 2001. Cloning and developmental expression of zebrafish GTP cyclohydrolase I. Mechanisms of Development 109:99-103.
Sato M, Nakazawa M, Usui T, Tanimoto N, Abe H, Ohguro H. 2005. Mutations in the gene coding for guanylate cyclase-activating protein 2 (GUCA1B gene) in patients with autosomal

dominant retinal dystrophies. Graefe's Archive for Clinical and Experimental Ophthalmology 243:235-242.
Vincent A, Audo I, Tavares E, Maynes Jason T, Tumber A, Wright T, Li S, Michiels C, Banin E, Bocquet B, et al. 2016. Biallelic Mutations in *GNB3* Cause a Unique Form of Autosomal-Recessive Congenital Stationary Night Blindness. The American Journal of Human Genetics 98:1011-1019.
Wasfy MM, Matsui JI, Miller J, Dowling JE, Perkins BD. 2014. myosin 7aa−/− mutant zebrafish show mild photoreceptor degeneration and reduced electroretinographic responses. Experimental Eye Research 122:65-76.
Zhao H, Di Mauro G, Lungu-Mitea S, Negrini P, Guarino AM, Frigato E, Braunbeck T, Ma H, Lamparter T, Vallone D, et al. 2018. Modulation of DNA Repair Systems in Blind Cavefish during Evolution in Constant Darkness. Current Biology 28:3229-3243.
