# Supplementary material for: Contrasting Gene Decay in Subterranean Vertebrates: Insights from Cavefishes and Fossorial Mammals
Source: Mol Biol Evol. 2020 Sep 28;38(2):589–605. doi: 10.1093/molbev/msaa249 (PMC7826195; doi:10.1093/molbev/msaa249)
Supplement: msaa249_Supplementary_Data [file msaa249_supplementary_data.zip › msaa249-suppl_data/Data S3 LoF mutations types rates distributions.pdf]

Types and numbers of LOF mutations found in cavefishes

A total of 118 mutations to a STOP codon, 148 frameshifts (84 deletions and 64 insertions), 5 STOP codon losses, 13 START codon losses and 40 intron splice site losses were identified in our dataset (**fig. 1A**). Most frameshifts were the results of very small deletions or insertions (1 or 2 bp) while a few of them were larger indels (**fig. 1B**). The largest deletion found was 83 bp long in *zic2b* (pigmentation gene) of *S. rhinocerous* and the largest insertion found was 20 bp long in *opn7b* (vision gene) of *S. anshuiensis*.

Estimation of LoF mutation rates per gene set per generation in cavefishes

As very few LoF mutations were found in *A. mexicanus* cavefish, estimations are based on the data from *Lucifuga* spp. and *Sinocyclocheilus* spp. only.

i) Rate of nucleotide mutations leading to a new STOP codon. The genetic code implies that among 549 (61 codons x 9 possible changes) mutations in sense codons, 23 lead to a STOP codon, that is ~ 4% if the frequency of each codon is 1/61 and transitions are as frequent as transversions. As among those 23 mutations, 5 are transitions and 18 are transversions, the transition/transversion ratio (*r*) can be taken into account to estimate more accurately the fraction of mutation leading to a STOP codon $f = (5r + 18)/(183r + 366)$. Using a R script, the estimation of *f* was further refined by taking into account codon frequencies (frequency_new_stop.py).
For vision genes, taking into account estimates of r in *Lucifuga* spp. and *Sinocyclocheilus* spp. (4.57 and 1.95 respectively) and the codon frequencies of their vision gene sequences, *f* was estimated equal to 0.031 and 0.037 respectively in these species. Moreover, taking into account that 13 internal STOP codons were found in *Lucifuga* spp. and 47 in *Sinocyclocheilus* spp., we estimated a weighted mean *f* = 0.036 for the whole vision gene dataset (as only one LoF mutation that is not a STOP codon was found in *A. mexicanus*, this species was excluded from this analysis).
Applying the same approach, we found *f* = 0.038 for the circadian clock genes and *f* = 0.036 for pigmentation genes.
For the three gene sets taken together, weighting by the length of the concatenated genes in each dataset, we estimated a global mean *f* = 0.036. Overall, these data showed that the impact of r and codon frequencies on the estimation of the frequency of mutations leading to a new STOP codon was limited.
For a set of coding sequences of length *l* (sum of the CDS lengths), the rate of mutation to a STOP codon $\mu_{STOP} = f\mu l$, where μ is the nucleotide mutation rate per site.

ii) Rate of frameshifts (*i.e.* rate of indels whose length modulo 3 ≠ 0). There is no theoretical expectation of this rate which is related to the nucleotide mutation rate per site. However, we linked the frameshift rate and the nucleotide mutation rate by taking into account the ratio of the number of frameshifts (n_f) and the number of new STOP codons (n_s).
For a set of coding sequences of length *l*, the rate of mutation to a frameshift $\mu_{frameshift} = \frac{n_f}{n_S}\mu_{STOP} = \frac{n_f}{n_S} f\mu l$

iii) Rate of mutations in splice sites.
This rate is independent of *l* but depends on the number of introns (n_i).
The rate of mutation at a splice site $= 4n_i\mu$ (where 4 is the number of splice sites per intron).

iv) Rate of START codon loss.
This rate depends only on the number of genes
The rate of START codon loss $= 3n_g\mu$ (where n_g is the number of genes).

v) Rate of STOP codon loss
This rate depends only on the number of genes.
The rate of STOP codon loss is $\frac{23}{27}3n_g\mu$ (where 27 is the number of different mutations in a codon and $\frac{4}{27}$ is the proportion of mutations in a STOP codon which leads to another STOP codon).

Globally, for a set of genes for which the sum of the CDS lengths is *l* and the number of intron is n_i, the LoF mutation rate is

$$\mu_G = \left[\left(1 + \frac{n_f}{n_S}\right) fl + 4n_i + \left(1 + \frac{23}{27}\right) 3n_g\right] \mu$$

For analytical models based on the assumption that all genes have the same LoF mutation rate (μ_{LoF}), we assume that $\mu_{LoF} = \frac{\mu_G}{n_g}$

In order to assess the effect of the variability of gene length and intron number in vision genes, a program was written to simulate gene decay through accumulation of LoF mutations, taking into account the length and the number of introns in each gene to compute a μ_{LoF} for each gene. At each generation and for each gene, the probability that a new LoF appears in one ancestral and functional allele at frequency q in a population of size N_e is: $2N_e q\mu_{LoF}$. When a new LoF mutation appears its frequency is $\frac{1}{2N_e}$ and the total frequency of LoF mutations is $p + 1/2N_e$, where $p = 1 - q$. We assumed random mating, no selection and no migration and a constant population size. Genetic drift between two generations was simulated taking into account the new allele frequencies if a mutation occurred, and $2N_e$ (the number of alleles sampled to generate the next generation).
The simulation program was written in Python (https://github.com/MaximePolicarpo/Molecular-decay-of-light-processing-genes-in-cavefishes/blob/master/SimulationScript.py).

Both approaches gave very similar results (see **fig. 6** in the article).

Observed versus expected frequencies of LoF mutations

We tested if the relative frequencies of the different types of LoF mutations (*i.e.* STOP codon gains, losses of START and STOP codons, losses of intron splice sites and small indels leading to frameshifts) were those expected under a neutral model, that is if their relative frequencies were proportional to their probabilities of occurrence. Taking into account a nucleotide mutation rate (μ), observed transition/transversion ratio and codon frequencies, the rate of mutation to a new STOP codon was $\mu_{stop} = 0.036\mu$ (see above for details on this estimation). Based on the ratio of frameshift/STOP mutations, we estimated the rate of indels leading to frameshifts ($\mu_{frameshift}$) as 148/118 x $\mu_{stop} = 0.05\mu$. The rate of mutations in splice acceptor or donor sites was estimated as: 4 x (number of introns) / Σ (CDS length) x μ, *i.e.* 4 x 34175 / 6154965 x μ = 0.022μ (where 34175 is the number of introns and 6154965 is the sum of the lengths of 3625 genes retrieved from the genomes of two *Lucifuga* species, three

Sinocyclocheilus species and two genomes of *A. mexicanus*). The rate of START codon loss was estimated as: 3 x (number of genes) / Σ (CDS length) x μ, *i.e.* 3 x 3625 / 6154965 x μ = 0.0018μ. The rate of STOP codon loss was estimated as: 3 x (number of genes) / Σ (CDS length) x 0.85 x μ, *i.e.* 3 x 3625 / 6154965 x 0.85 x μ = 0.0015μ (where 0.85 is the probability that a mutation in a STOP codon leads to a sense codon). The observed distribution of LoF mutations fit well with the expected distribution (**fig. 1A**). These results suggested that the number of LoF mutations of each type is proportional to its probability of occurrence.

Randomness of LoF mutation positions within the genes

In order to test if LoF mutations were distributed randomly within the genes, that is if they were not clustered at the 3' end of the genes where their deleterious effect could be small, we computed the effective number of gene segments generated by LoF mutations and compared this value with simulations of random distributions of mutations within genes. This method was initially designed for estimating the randomness of intron insertions (Lynch and Kewalramani 2003). The effective number of gene segments is: $n_s = 1/\sum_{i=1}^{n} s_i^2$, with n being the number of segments of genes separated by n-1 LoF mutations and s_i being the length of the ith segment. As LoF mutations are found in genes with different lengths, the position of each LoF mutation was normalized by dividing by the length of the coding sequence, the sum of s_i was thus equal to 1 for each gene. The most extreme case of LoF dispersion is the one in which all segments are of the same length ($1/n$), *i.e.* the LoF mutations are regularly spaced out, yielding $n_s = n$. On the other hand, if all LoF are clustered at one end of the genes, one segment approaches length 1.0, while all others approach 0.0, yielding $n_s = 1$. In order to obtain the distribution of the values of n_s under the null model that LoF mutations are distributed randomly within the genes, 100,000 simulations of random distribution of the observed number of LoF mutations within a gene of length 1.0 were performed.

We found that premature STOP codons and frameshifts are distributed randomly within coding sequences in all sets of genes (**fig. 2A and fig. 2B** respectively).

References

Lynch M, Kewalramani A. 2003. Messenger RNA Surveillance and the Evolutionary Proliferation of Introns. Molecular Biology and Evolution 20:563-571.

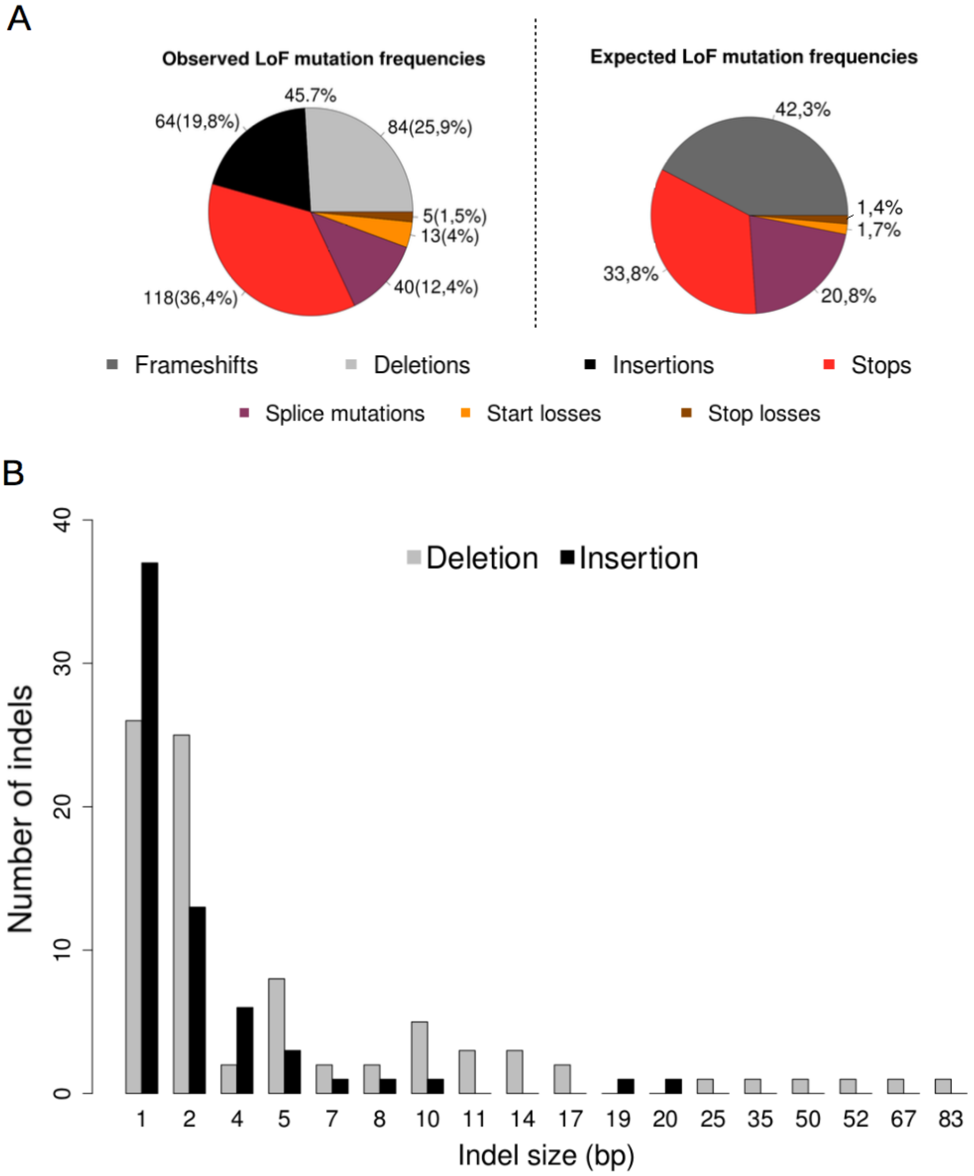

Figure 1. Distribution of different categories of LoF mutations. (A) Observed and expected frequencies. (B) Distribution of indel size.

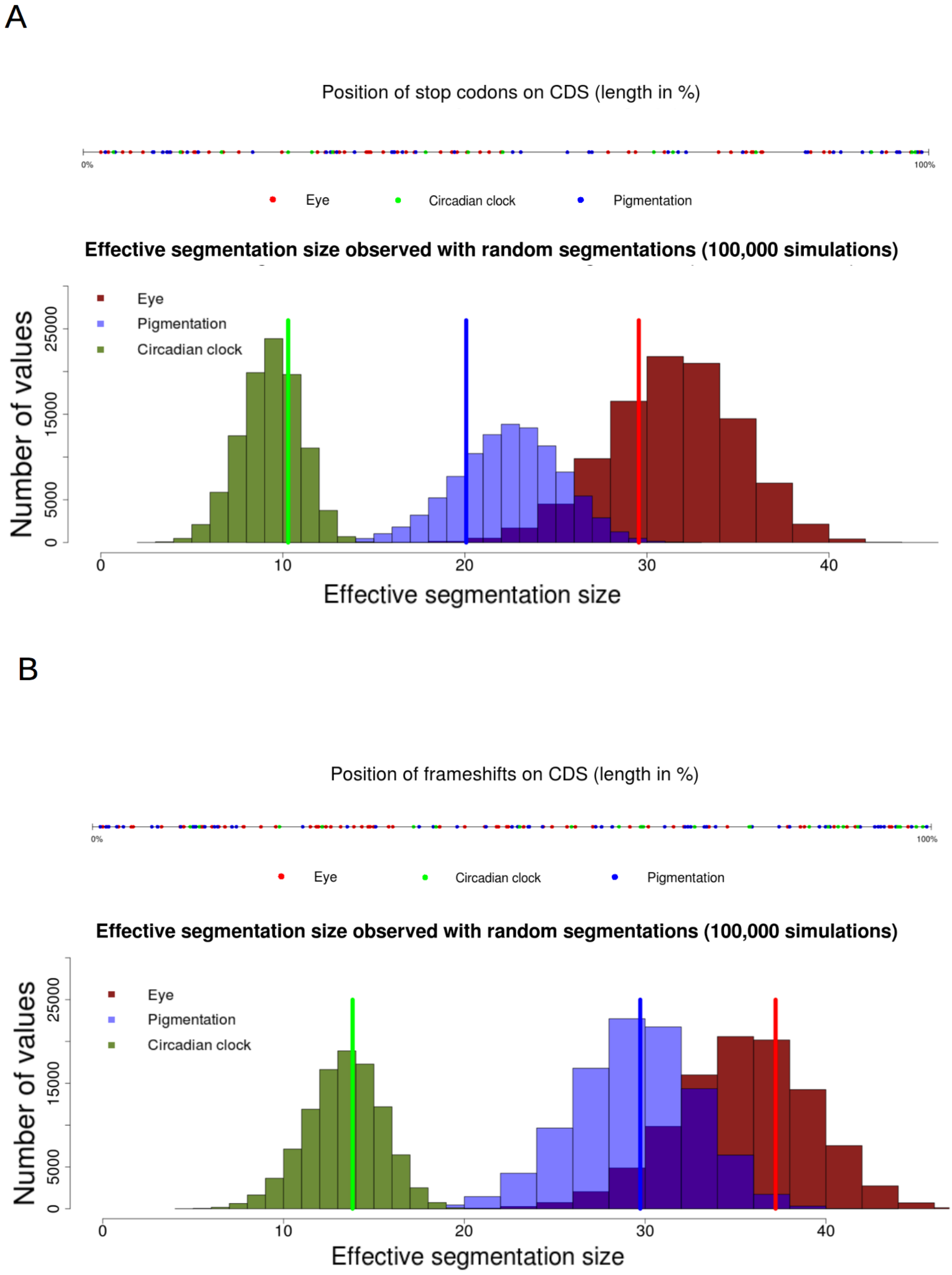

Figure 2. Distribution of the effective segment size generated by random insertion of STOP codons (A) and frameshifts (B) (100,000 simulations).
