# Supplementary material for: Contrasting Gene Decay in Subterranean Vertebrates: Insights from Cavefishes and Fossorial Mammals
Source: Mol Biol Evol. 2020 Sep 28;38(2):589–605. doi: 10.1093/molbev/msaa249 (PMC7826195; doi:10.1093/molbev/msaa249)

Supplementary Material - Tables and Figures

Table S1. List of LoF mutations found in *Lucifuga dentata* and *Lucifuga gibarensis* genomes, and their coverage. LoF mutations in red were also found in the transcriptome of *L. dentata*.

Table S2. Log-likelihood values (lnL), parameter estimates (ω) and likelihood ratio tests (LRT) for three nested branch models testing for relaxed purifying selection on vision, circadian clock and pigmentation genes in diploid and tetraploid blind cavefishes. 2ΔlnL: twice the difference of log-likelihood between the two models compared. d.f.: number of degrees of freedom.

Fig. S1. List of vision genes retrieved from cavefishes and related species. Colors represent the type of LoF mutation. When higher than one, the number of LoF mutations is also reported.

Fig. S2. Photos of the specimens used for genome sequencing. (A) *Lucifuga dentata* from Emilio cave, Las Cañas, Artemisa Province. (B) *Lucifuga gibarensis* from Aguada de Macigo del Jobal, Gibara.

Fig. S3. (A) Number of differences per gene between *Astyanax mexicanus* morphs and between *Lucifuga dentata* and *Lucifuga gibarensis*. (B) Estimation of ω for each vision gene. Grey domains contain values of dn and/or ds < 0.01, giving unreliable estimations of ω when the number of mutations is very low.

Fig. S4. Estimations of ω with concatenated sequences. Branch colors are scaled depending on ω values. Trees were visualized using ggtree (Yu, G., Smith, D.K., Zhu, H., Guan, Y. and Lam, T.T.-Y. (2017), ggtree: an R package for visualization and annotation of phylogenetic trees with their covariates and other associated data. Methods Ecol Evol, 8: 28-36.

Fig. S5. RELAX results with cavefish species assigned as test branch for vision genes. The k parameter and p-value are displayed along with ω plots.

Fig. S6. RELAX results with cavefish species assigned as test branch for circadian clock genes. The k parameter and p-value are displayed along with ω plots.

Fig. S7. RELAX results with cavefish species assigned as test branch for pigmentation genes. The k parameter and p-value are displayed along with ω plots.

Fig. S8. Effect of the Transition/Transversion ratio on the cumulative distribution of MutPred2 scores obtained after simulations of nonsynonymous mutations.

Fig. S9. Empirical cumulative distributions of MutPred2 scores. The number of scores is indicated between parenthesis. One hundred simulations were performed for each dataset, each simulation generating 54 random nonsynonymous mutations in vision genes, 36 in circadian clock genes and 232 in pigmentation genes, which are the number of nonsynonymous mutations found in *Astyanax mexicanus* cavefish. The statistical significance of the difference between each pair of distributions was assessed using the Kolmogorov-Smirnov test (significant differences are shown on a red background whereas non-significant differences are shown on a green background).

Fig. S10. Empirical cumulative distributions of Grantham's distances. The distance between two amino acids increases with their chemical dissimilarity which is computed using their composition, polarity and molecular volume. The number of distances is indicated between parenthesis. One hundred simulations were performed for each dataset, each simulation generating 54 random nonsynonymous mutations in vision genes, 36 in circadian clock genes and 232 in pigmentation genes which are the number of nonsynonymous mutations found in *Astyanax mexicanus* cavefish. The statistical significance of the difference between each pair of distributions was assessed using the Kolmogorov-Smirnov test (significant differences are shown on a red background whereas non-significant differences are shown on a green background).

Fig. S11. Fit of mixture distributions of MutPred2 scores with the distributions found in two *Lucifuga* spp. and two *Astyanax mexicanus* morphs. The p-value of the Kolomogorv-Smirnov test between an observed distribution and a mixture distribution was plotted according to the proportion of mutations under completely relaxed selection in the mixture distribution.

Fig. S12. Distribution of MutPred2 scores in three *Sinocyclocheilus* species, *Danio rerio* and in simulations of mutations. The number of mutations in each lineage is given between parenthesis. One hundred simulations were performed with each gene set. In each simulation 54 nonsynonymous mutations were generated in vision genes, 36 in circadian clock genes and 232 in pigmentation genes, those numbers corresponding to the numbers of nonsynonymous mutations found in *Astyanax mexicanus* cavefish.

Fig. S13. Fit of mixture distributions of MutPred2 scores with the distributions found in three *Sinocyclocheilus* species. The p-value of the Kolomogorv-Smirnov test between an observed distribution and a mixture distribution was plotted according to the proportion of mutations under completely relaxed selection in the mixture distribution.

Table S1. LoF mutations coverage for *L.dentata* and *L.gibarensis*

Species	Gene	LoF mutation	Coverage
Lucifuga dentata	opn4m3	Stop codon (CGA->TGA)	87
Lucifuga dentata	opn4m3	Frameshift (2bp deletion)	111
Lucifuga dentata	opn7a	Stop codon (CGA->TGA)	104
Lucifuga dentata	opn7b	Stop codon (TCA->TAA)	95
Lucifuga dentata	parapinopsin-1	Stop codon (CAG->TAG)	121
Lucifuga dentata	parietopsin	Frameshift (2bp insertion)	115
Lucifuga dentata	rgr1	Stop codon (CAA->TAA)	101
Lucifuga gibarensis	rgr1	Splice site mutation (Intron2 : GT-AC)	18
Lucifuga dentata<br>*Lucifuga gibarensis*	rgr1	Splice site mutation (Intron4 : GT-TG)	49<br>33
Lucifuga dentata	tmt1a	Splice site mutation (Intron3 : GC-AC)	95
Lucifuga gibarensis	tmt1a	Splice site mutation (Intron3 : GT-AC)	9
Lucifuga gibarensis	tmt3a	Stop codon (CGA->TGA)	86
Lucifuga dentata	tmt3b	Stop codon (CGA->TGA)	107
Lucifuga dentata	cryaa	Frameshift (4bp deletion)	93
Lucifuga dentata	crybb1	Splice site mutation (Intron2 : TT-AG)	121
Lucifuga dentata	crybgx	Stop codon (CAG->TAG)	92
Lucifuga dentata	crybgx	Splice site mutation (Intron2 : GT-AA)	108
Lucifuga dentata	crygn2	Splice site mutation (Intron3 : GT-GG)	103
Lucifuga dentata	gcap2	Splice site mutation (Intron1 : AT-AG)	116
Lucifuga gibarensis	gcap2	Stop codon (CAG->TAG)	C:45 | T:56
Lucifuga gibarensis	gcap2	Splice site mutation (Intron3 : TG-AG)	TG:65 | GT:58
Lucifuga dentata	grk1b	Frameshift (2bp deletion)	115
Lucifuga dentata	grk7a	Stop codon (CGA->TGA)	117
Lucifuga dentata	pde6ga	Stop codon (AGG->TGA)	92
Lucifuga gibarensis	pde6hb	Frameshift (1bp insertion)	19
Lucifuga dentata	gc2	Frameshift (1bp deletion)	110
Lucifuga dentata	gc3	Stop codon (TGG->TGA)	103
Lucifuga dentata	gnb3b	Stop codon (CGA->TGA)	85

LoF mutations highlighted in red were retrieved independently in the RNA assembly with the following transcript id ; opn4m3 : TRINITY_DN73481_c0_g1_i1 ; rgr1 : TRINITY_DN84788_c2_g1_i3 ; tmt3b : TRINITY_DN63991_c0_g1_i1 ; cryaa : TRINITY_DN176044_c0_g1_i1 ;
crybb1 : TRINITY_DN79623_c2_g4_i1 ; crygn2 : TRINITY_DN68647_c0_g1_i1 ;
pde6ga : TRINITY_DN116881_c0_g1_i1 ; gc3 : TRINITY_DN172788_c0_g1_i1 ;
gnb3b : TRINITY_DN59054_c0_g1_i1

Table S2. codeml analyses of vision, circadian clock and pigmentation genes

	Species	Model	lnL	ω	Models tested	2ΔlnL	d.f.	LRT p-value
Vision	Diploid species	One-ratio	-525339	0.09				
		Two-ratio	-525207	ω_{SF} = 0.09<br>ω_{CF} = 0.45	Two-ratio vs one-ratio model	264	1	<0.001
		**Free-ratio**	**-524846**	**(See Fig. S4)**	**Free-ratio vs two-ratio model**	**722**	29	**<0.001**
	Tetraploid species	One-ratio	-163295	0.15				
		Two-ratio	-163206	ω_{SF} = 0.14<br>ω_{CF} = 0.38	Two-ratio vs one-ratio model	176	1	<0.001
		**Free-ratio**	**-163027**	**(See Fig. S4)**	**Free-ratio vs two-ratio model**	**359**	10	**<0.001**
Circadian clock	Diploid species	One-ratio	-344394	0.07				
		Two-ratio	-344335	ω_{SF} = 0.07<br>ω_{CF} = 0.34	Two-ratio vs one-ratio model	119	1	<0.001
		**Free-ratio**	**-344110**	**(See Fig. S4)**	**Free-ratio vs two-ratio model**	**451**	29	**<0.001**
	Tetraploid species	One-ratio	-129110	0.14				
		Two-ratio	-128995	ω_{SF} = 0.12<br>ω_{CF} = 0.39	Two-ratio vs one-ratio model	230	1	<0.001
		**Free-ratio**	**-128689**	**(See Fig. S4)**	**Free-ratio vs two-ratio model**	**612**	10	**<0.001**
Pigmentation	Diploid species	One-ratio	-2736779	0.08				
		Two-ratio	-2736632	ω_{SF} = 0.08<br>ω_{CF} = 0.19	Two-ratio vs one-ratio model	294	1	<0.001
		**Free-ratio**	**-2735261**	**(See Fig. S4)**	**Free-ratio vs two-ratio model**	**2742**	29	**<0.001**
	Tetraploid species	One-ratio	-974774	0.15				
		Two-ratio	-974480	ω_{SF} = 0.14<br>ω_{CF} = 0.30	Two-ratio vs one-ratio model	589	1	<0.001
		**Free-ratio**	**-973379**	**(See Fig. S4)**	**Free-ratio vs two-ratio model**	**2202**	10	**<0.001**

Fig. S1

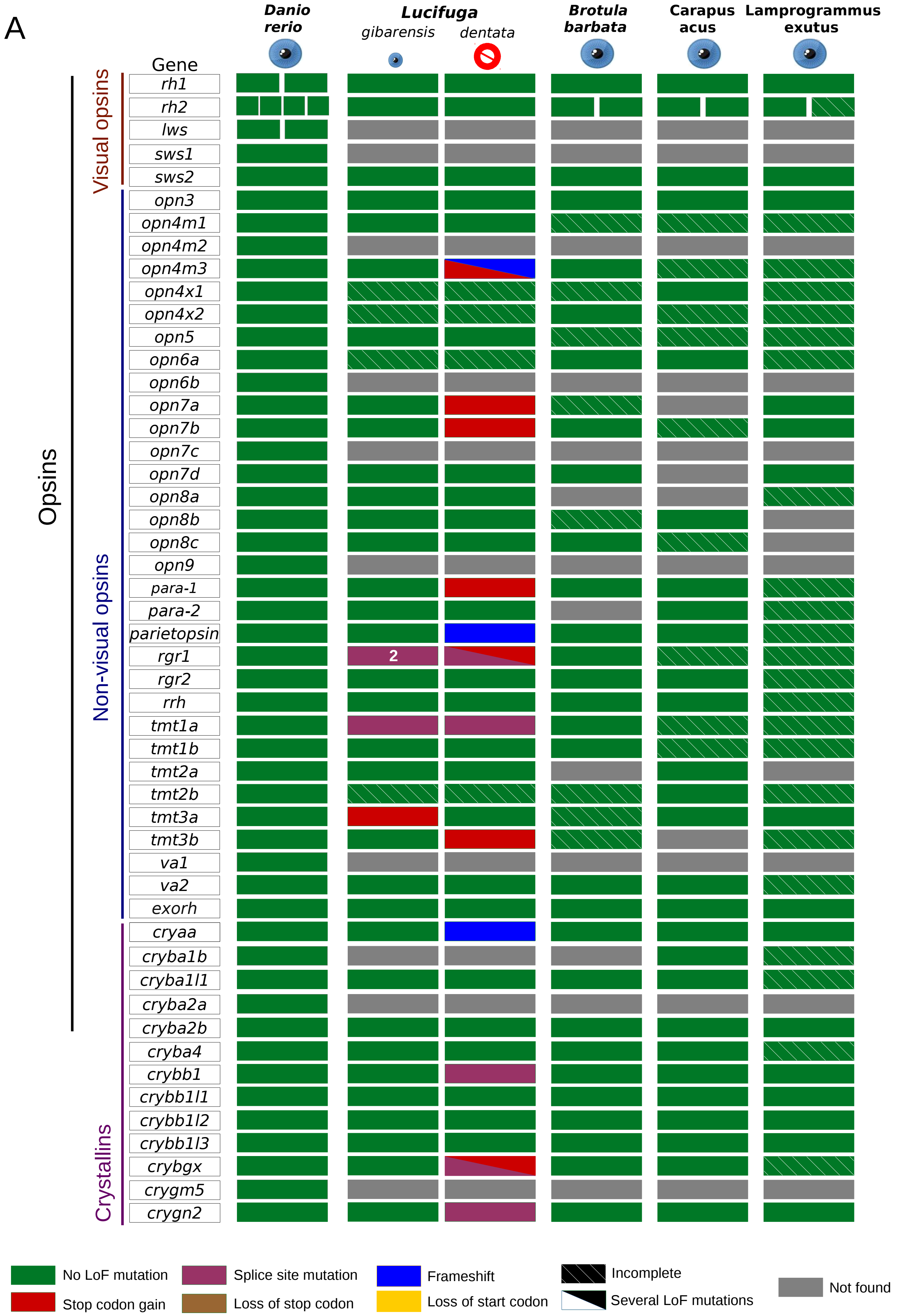
A
Danio rerio
Lucifuga
gibarensis
dentata
Brotula barbata
Carapus acus
Lamprogrammus exutus
Gene
Opsins
Visual opsins
Non-visual opsins
Crystallins
rh1
rh2
lws
sws1
sws2
opn3
opn4m1
opn4m2
opn4m3
opn4x1
opn4x2
opn5
opn6a
opn6b
opn7a
opn7b
opn7c
opn7d
opn8a
opn8b
opn8c
opn9
para-1
para-2
parietopsin
rgr1
rgr2
rrh
tmt1a
tmt1b
tmt2a
tmt2b
tmt3a
tmt3b
va1
va2
exorh
cryaa
cryba1b
cryba1l1
cryba2a
cryba2b
cryba4
crybb1
crybb1l1
crybb1l2
crybb1l3
crybgx
crygm5
crygn2
2
No LoF mutation
Stop codon gain
Splice site mutation
Loss of stop codon
Frameshift
Loss of start codon
Incomplete
Several LoF mutations
Not found

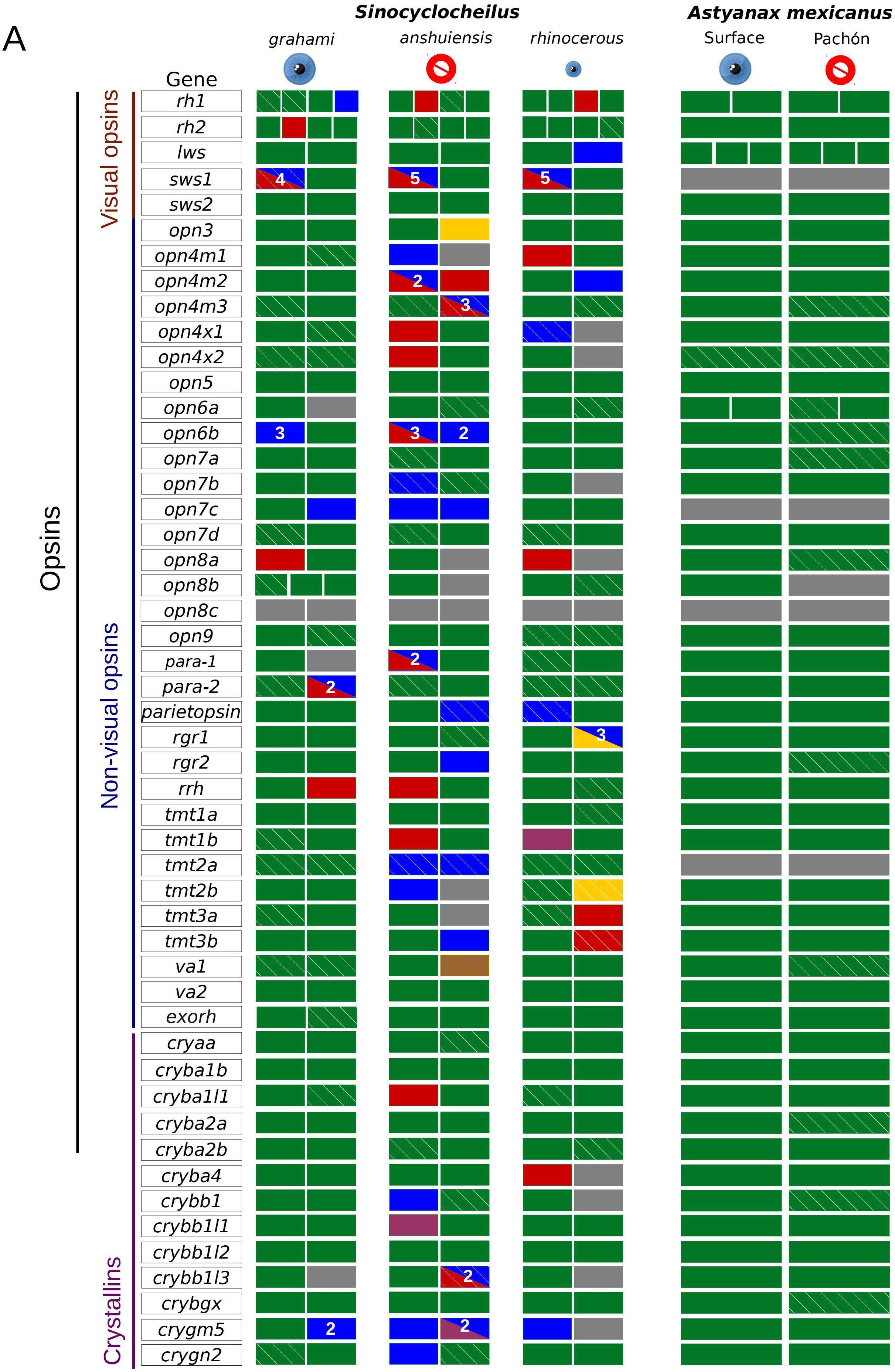
A
Sinocyclocheilus
grahami
anshuiensis
rhinocerous
Astyanax mexicanus
Surface
Pachón
Gene
Opsins
Visual opsins
Non-visual opsins
Crystallins
rh1
rh2
lws
sws1
sws2
opn3
opn4m1
opn4m2
opn4m3
opn4x1
opn4x2
opn5
opn6a
opn6b
opn7a
opn7b
opn7c
opn7d
opn8a
opn8b
opn8c
opn9
para-1
para-2
parietopsin
rgr1
rgr2
rrh
tmt1a
tmt1b
tmt2a
tmt2b
tmt3a
tmt3b
va1
va2
exorh
cryaa
cryba1b
cryba1l1
cryba2a
cryba2b
cryba4
crybb1
crybb1l1
crybb1l2
crybb1l3
crybgx
crygm5
crygn2

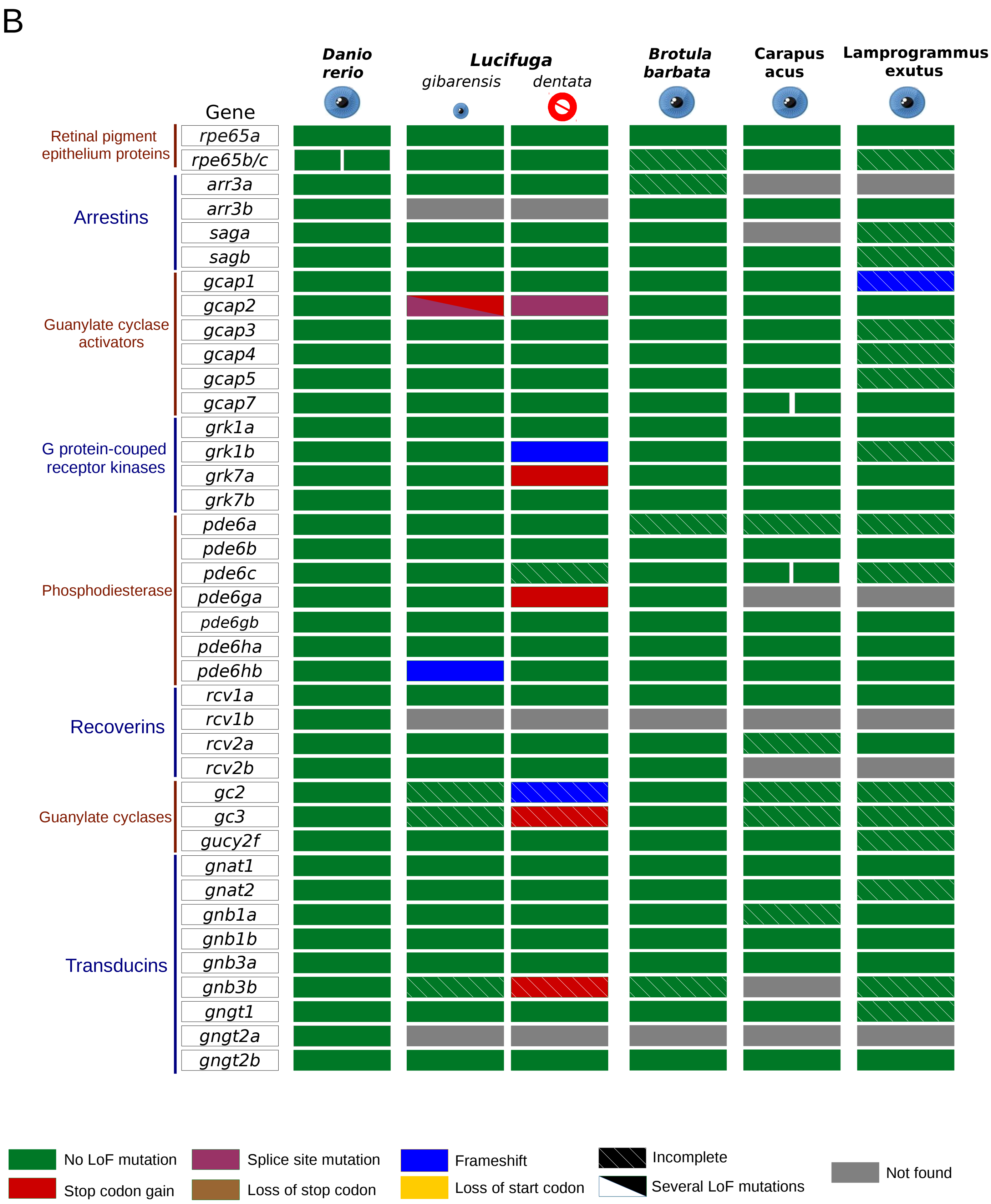
B
Danio rerio
Lucifuga
gibarensis
dentata
Brotula barbata
Carapus acus
Lamprogrammus exutus
Gene
Retinal pigment epithelium proteins
rpe65a
rpe65b/c
Arrestins
arr3a
arr3b
saga
sagb
Guanylate cyclase activators
gcap1
gcap2
gcap3
gcap4
gcap5
gcap7
G protein-couped receptor kinases
grk1a
grk1b
grk7a
grk7b
Phosphodiesterase
pde6a
pde6b
pde6c
pde6ga
pde6gb
pde6ha
pde6hb
Recoverins
rcv1a
rcv1b
rcv2a
rcv2b
Guanylate cyclases
gc2
gc3
gucy2f
Transducins
gnat1
gnat2
gnb1a
gnb1b
gnb3a
gnb3b
gngt1
gngt2a
gngt2b
No LoF mutation
Stop codon gain
Splice site mutation
Loss of stop codon
Frameshift
Loss of start codon
Incomplete
Several LoF mutations
Not found

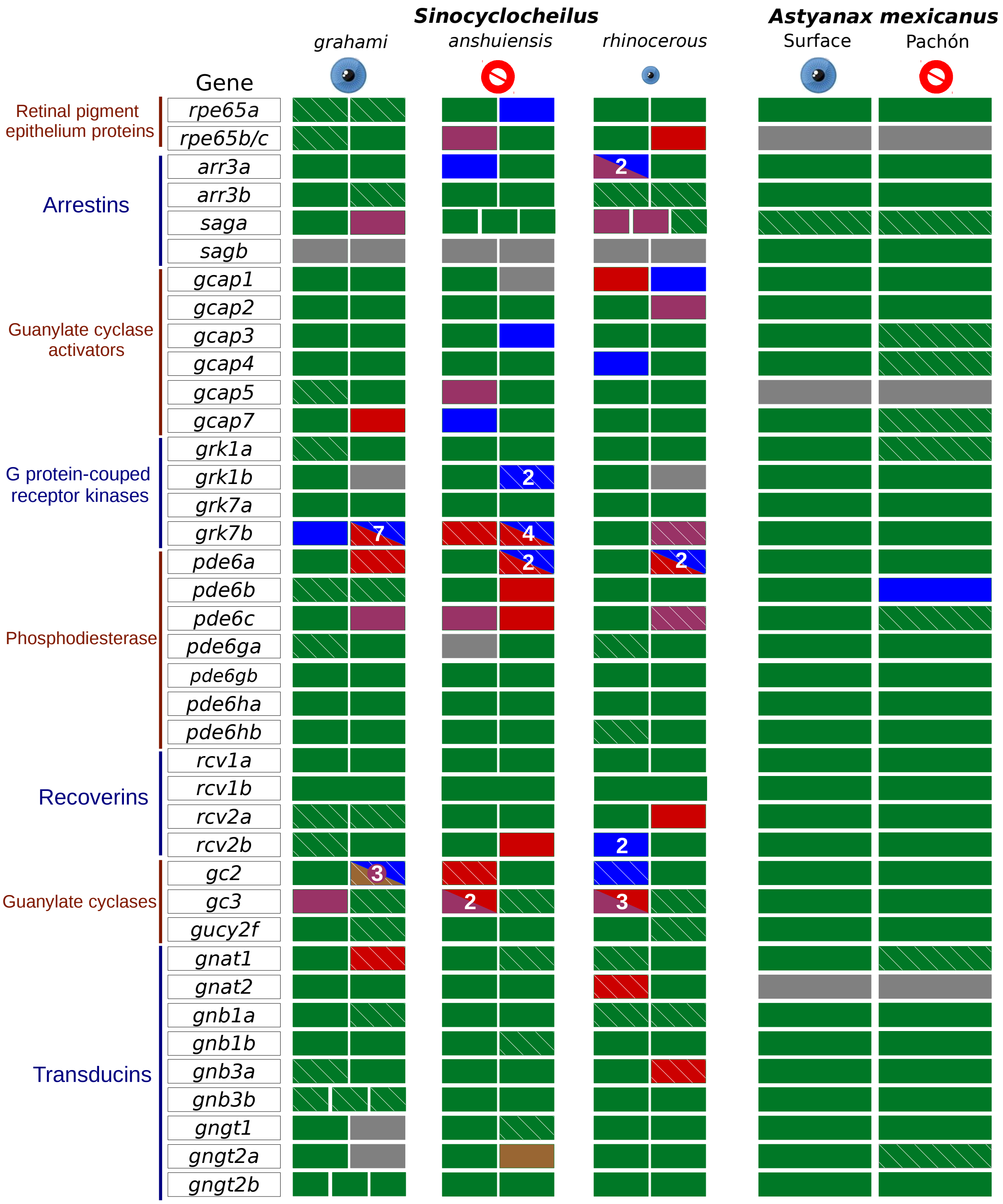

Sinocyclocheilus
grahami
anshuiensis
rhinocerous
Astyanax mexicanus
Surface
Pachón
Gene
Retinal pigment epithelium proteins
rpe65a
rpe65b/c
Arrestins
arr3a
arr3b
saga
sagb
Guanylate cyclase activators
gcap1
gcap2
gcap3
gcap4
gcap5
gcap7
G protein-couped receptor kinases
grk1a
grk1b
grk7a
grk7b
Phosphodiesterase
pde6a
pde6b
pde6c
pde6ga
pde6gb
pde6ha
pde6hb
Recoverins
rcv1a
rcv1b
rcv2a
rcv2b
Guanylate cyclases
gc2
gc3
gucy2f
Transducins
gnat1
gnat2
gnb1a
gnb1b
gnb3a
gnb3b
gngt1
gngt2a
gngt2b

Fig. S2

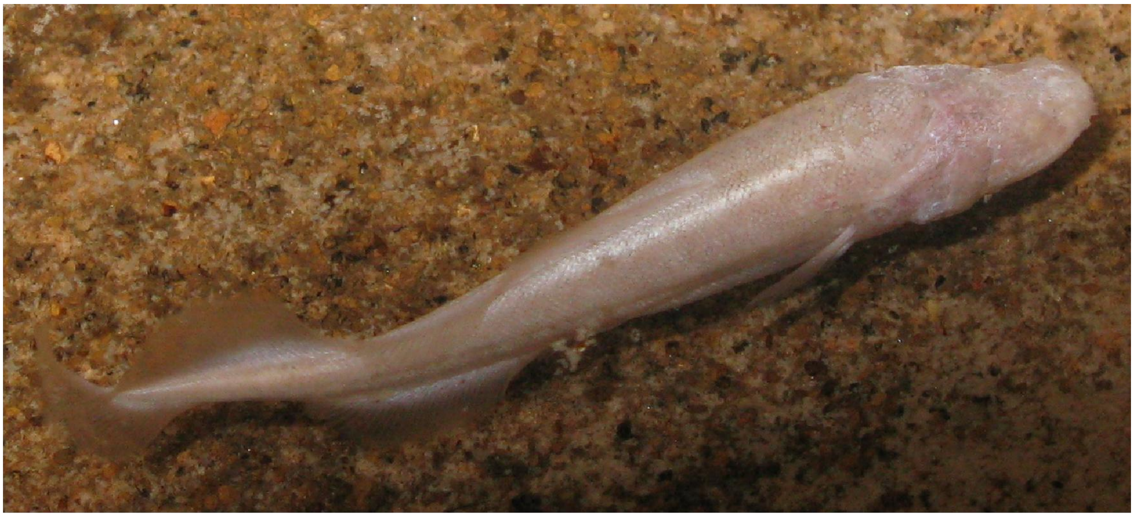

Lucifuga dentata

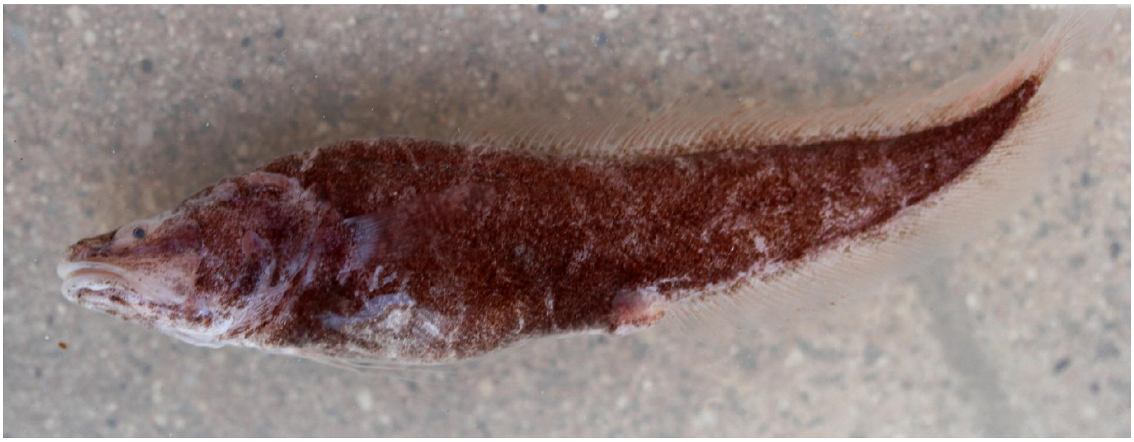

Lucifuga gibarensis

Fig. S3

A

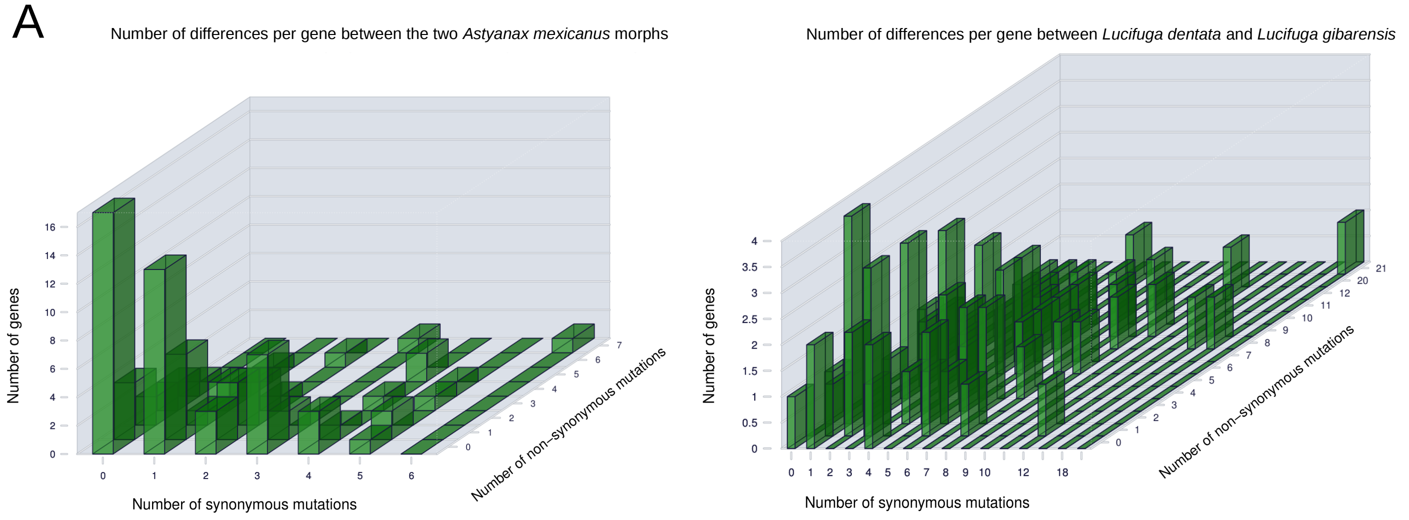

Number of differences per gene between the two *Astyanax mexicanus* morphs
Number of genes
Number of synonymous mutations
Number of non−synonymous mutations
Number of differences per gene between *Lucifuga dentata* and *Lucifuga gibarensis*
Number of genes
Number of synonymous mutations
Number of non−synonymous mutations

B

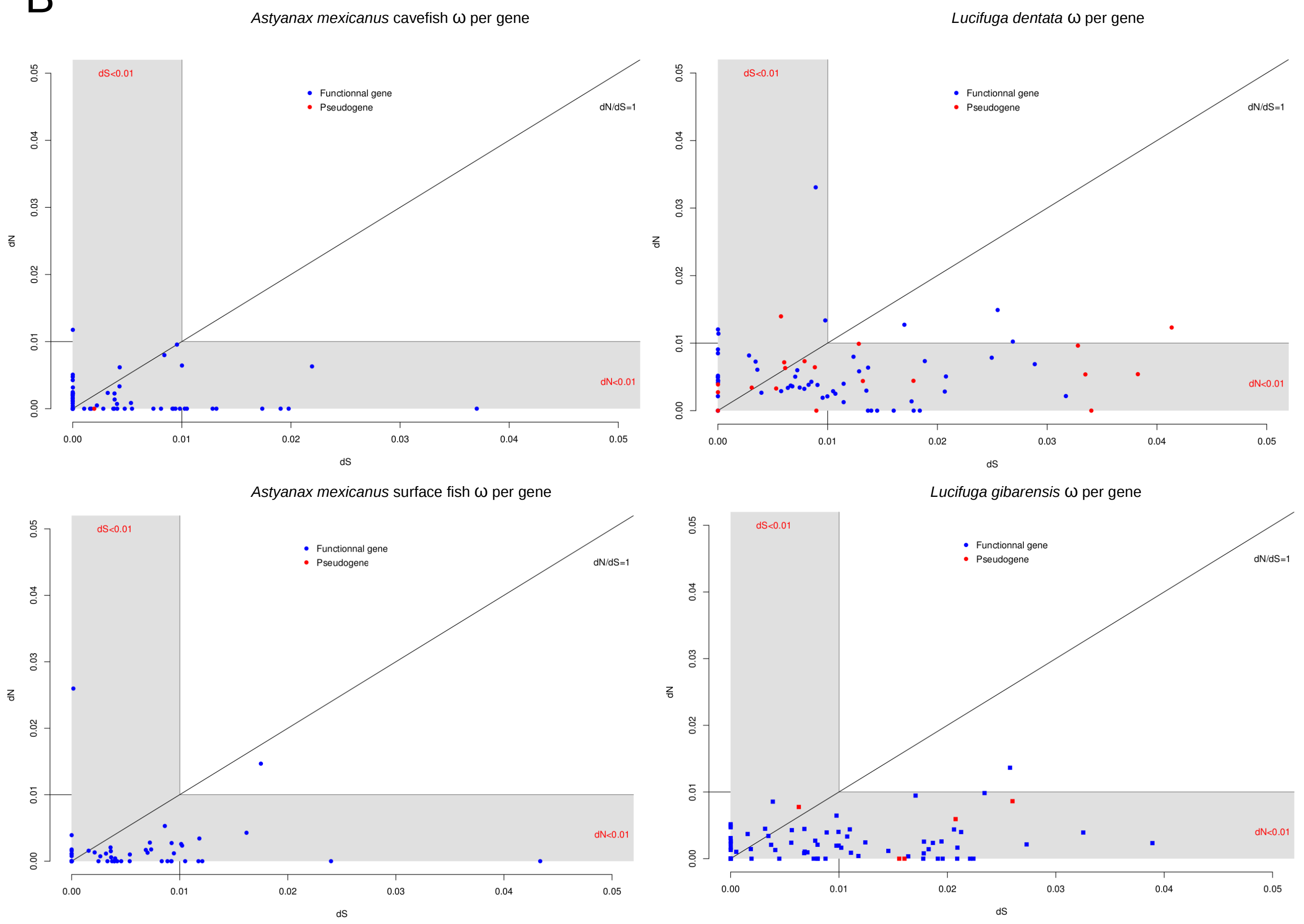

Astyanax mexicanus cavefish ω per gene
dS<0.01
Functionnal gene
Pseudogene
dN/dS=1
dN<0.01
dN
dS
Lucifuga dentata ω per gene
dS<0.01
Functionnal gene
Pseudogene
dN/dS=1
dN<0.01
dN
dS
Astyanax mexicanus surface fish ω per gene
dS<0.01
Functionnal gene
Pseudogene
dN/dS=1
dN<0.01
dN
dS
Lucifuga gibarensis ω per gene
dS<0.01
Functionnal gene
Pseudogene
dN/dS=1
dN<0.01
dN
dS

Fig. S4

A

Concatenated vision genes

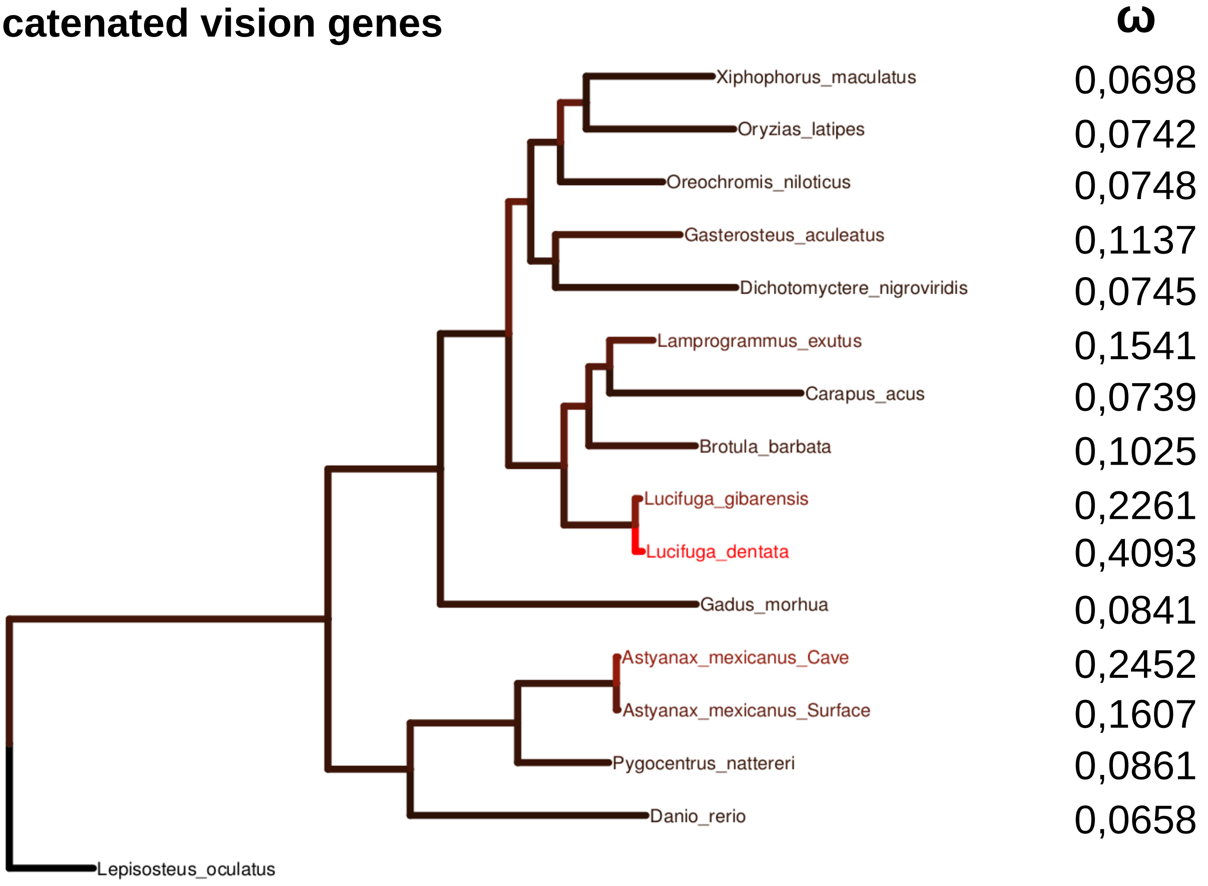

B

Concatenated circadian clock genes

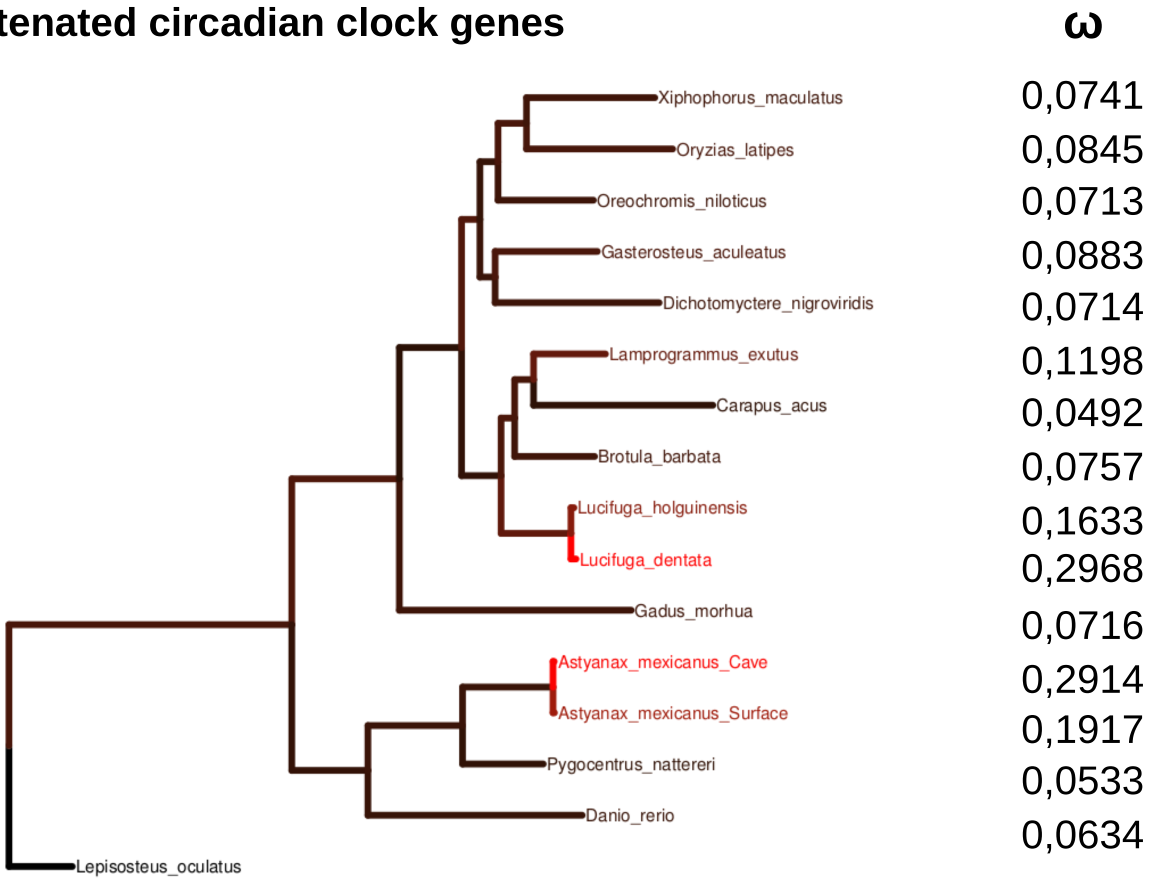

C

Concatenated pigmentation genes ω

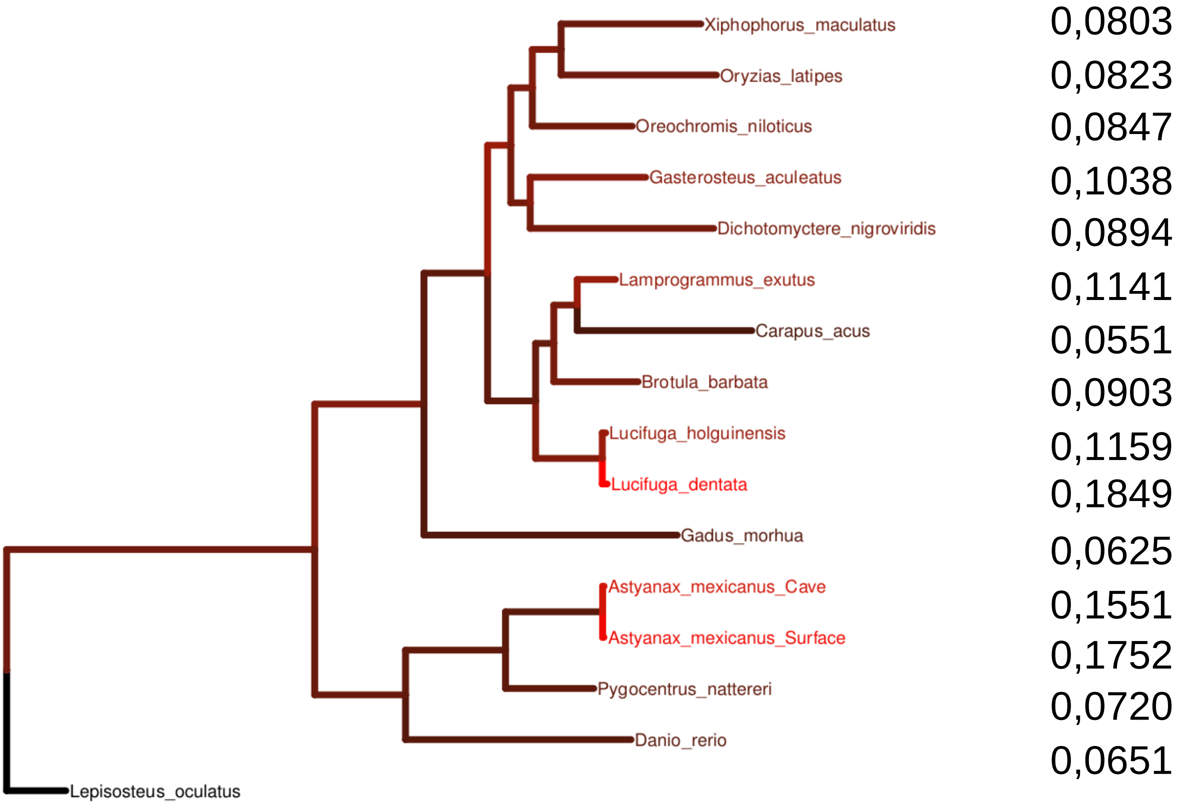

D

Concatenated vision genes - Sinocyclocheilus ω

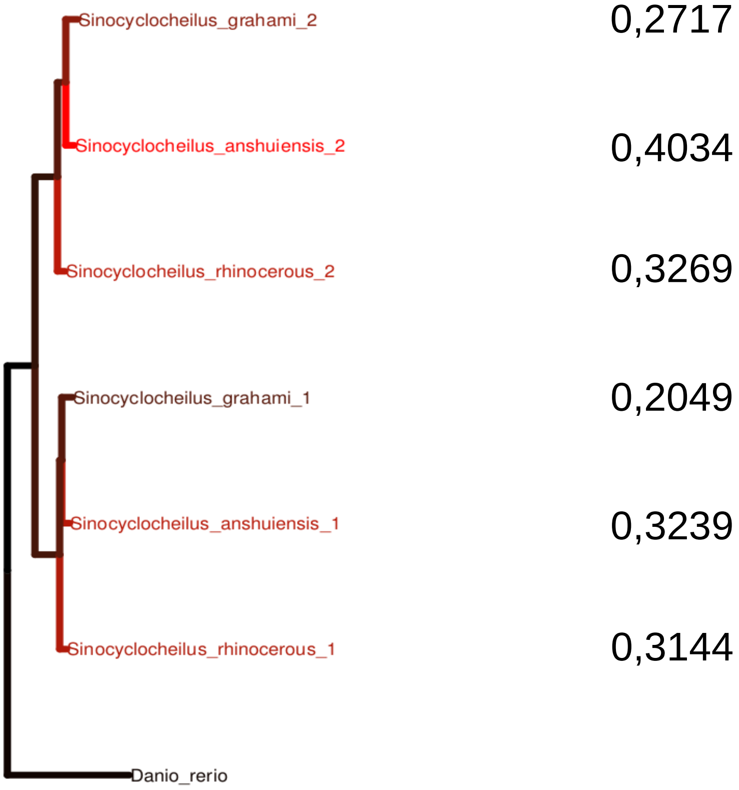

E

Concatenated circadian clock genes - Sinocyclocheilus

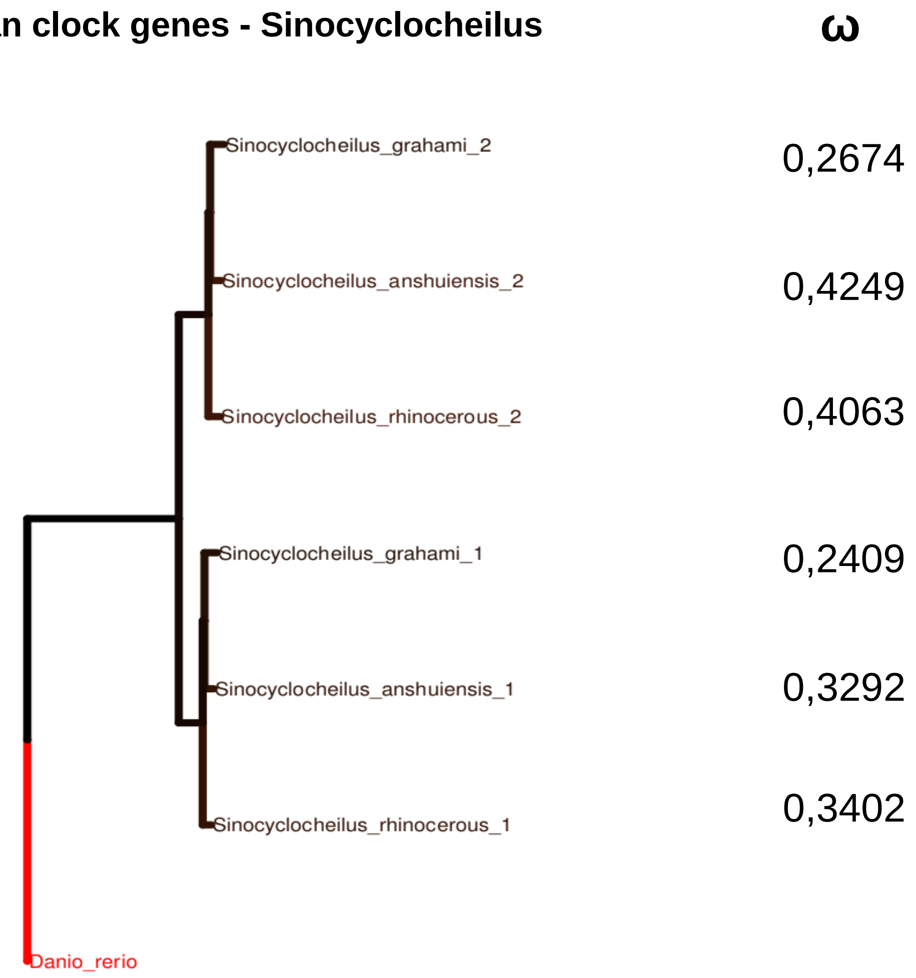

F

Concatenated pigmentation genes - Sinocyclocheilus

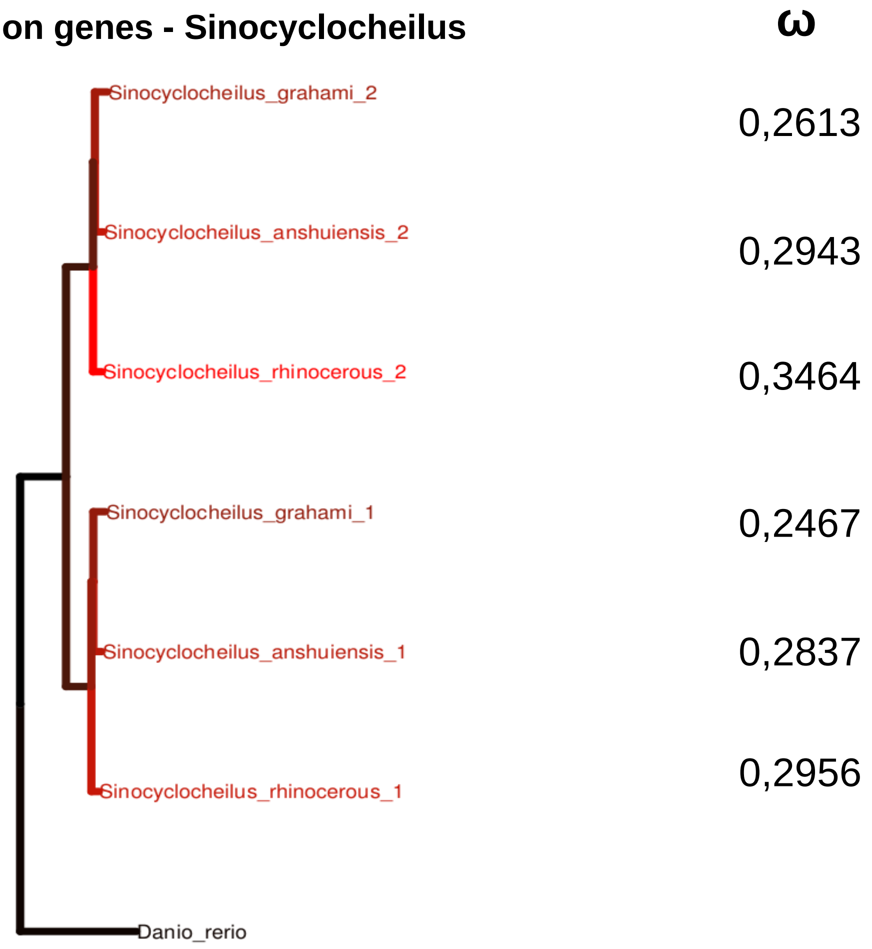

Fig. S5

Astyanax mexicanus CF

Test for selection **relaxation** (K = **0.50**) was **significant** (p = **0.021**, LR = **5.37**).

See here for more information about this method.
Please cite PMID 123456789 if you use this result in a publication, presentation, or other scientific work.

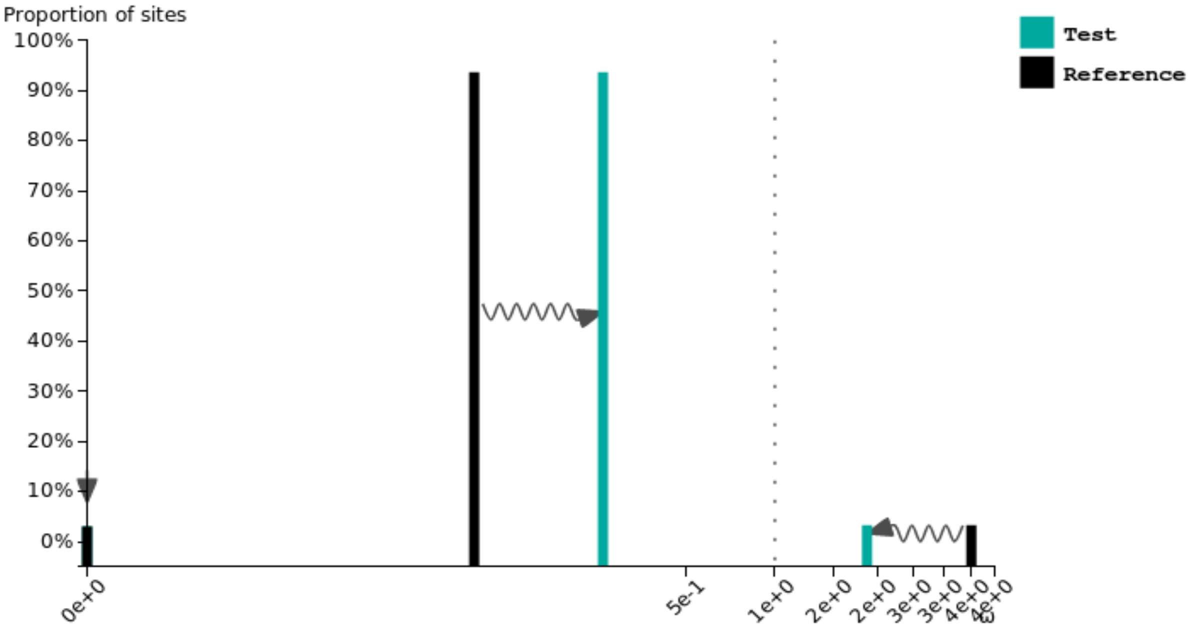

Lucifuga gibarensis

Test for selection **intensification** (K = **1.63**) was **significant** (p = **0.004**, LR = **8.34**).

See here for more information about this method.
Please cite PMID 123456789 if you use this result in a publication, presentation, or other scientific work.

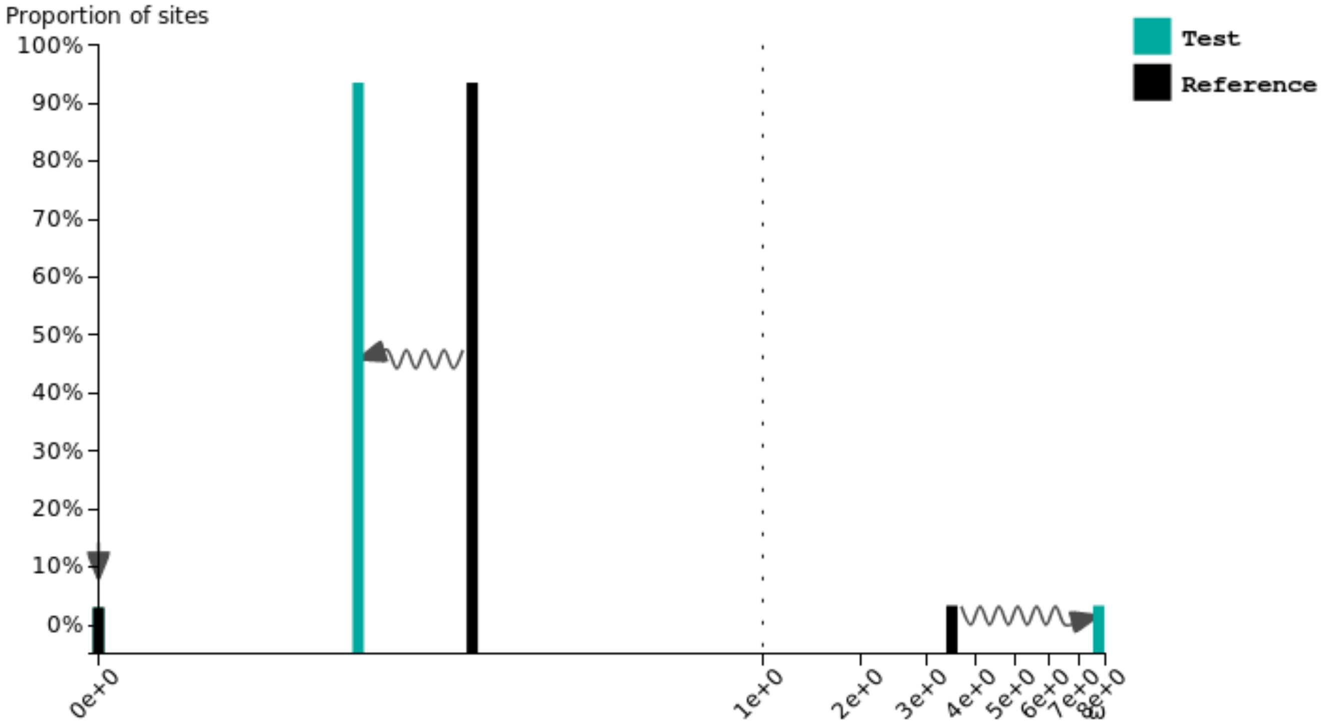

Lucifuga dentata

Test for selection **relaxation** (K = **0.20**) was **significant** (p = **0.000**, LR = **82.56**).

See here for more information about this method.

Please cite PMID 123456789 if you use this result in a publication, presentation, or other scientific work.

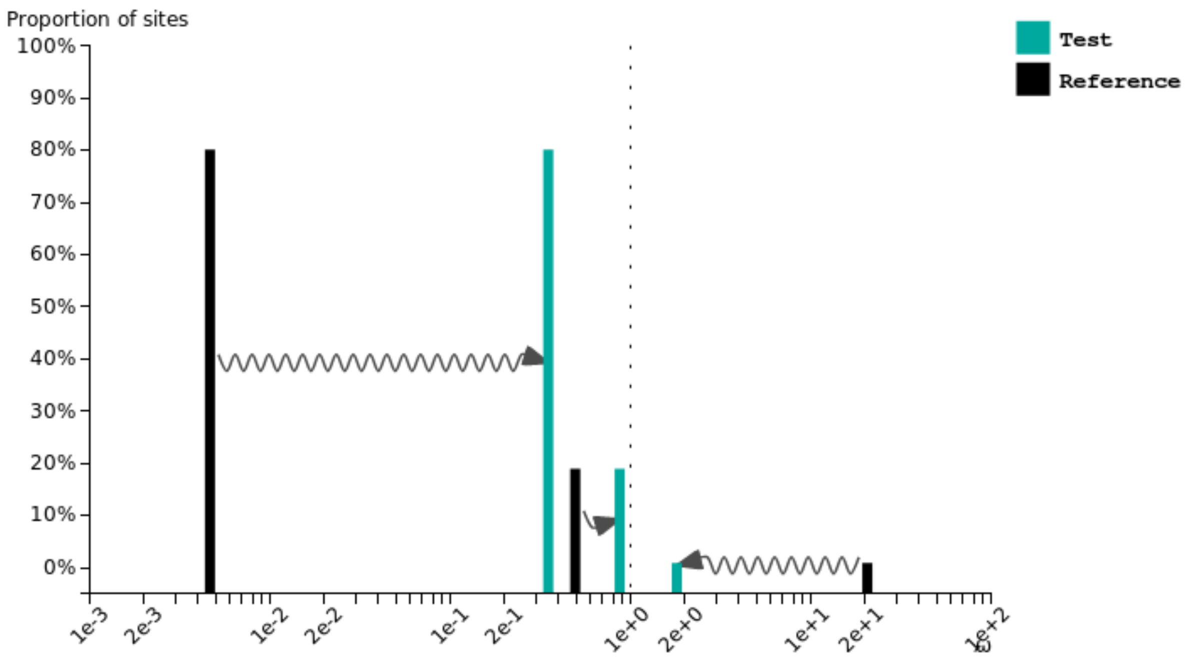

Fig. S6

Astyanax mexicanus CF

Test for selection **intensification** (K = **1.22**) was **not significant** (p = **0.195**, LR = **1.68**).

See here for more information about this method.

Please cite PMID 123456789 if you use this result in a publication, presentation, or other scientific work.

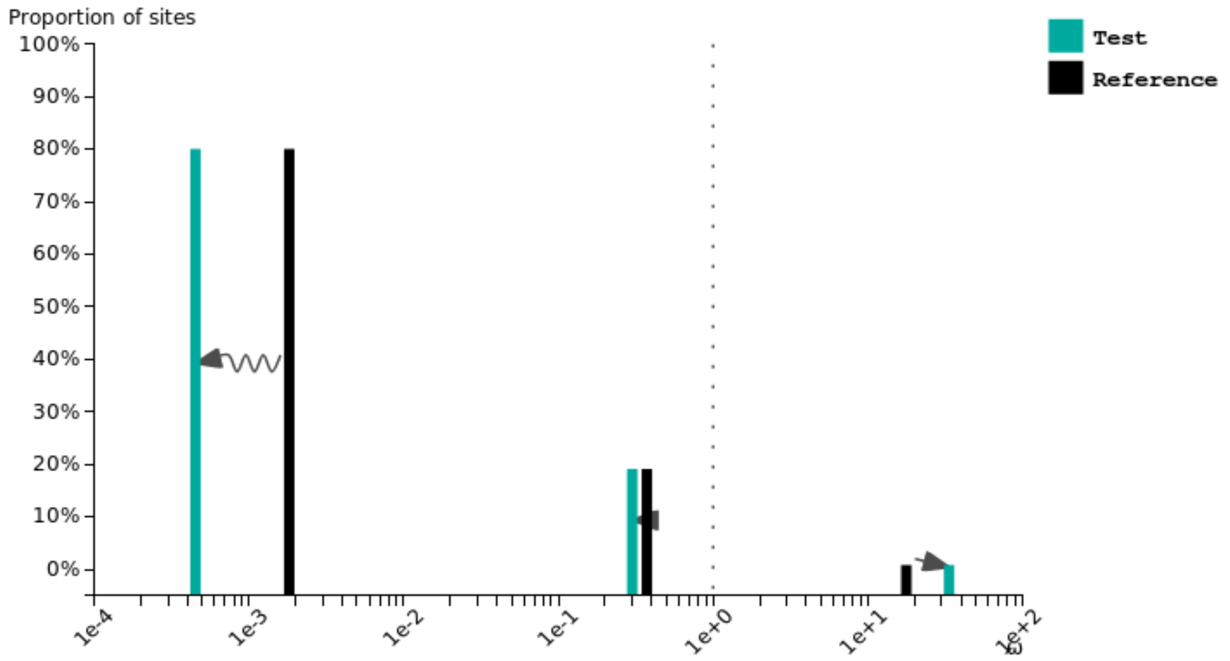

Lucifuga gibarensis

Test for selection **intensification** (K = **1.00**) was **significant** (p = **0.001**, LR = **10.96**).

See here for more information about this method.

Please cite PMID 123456789 if you use this result in a publication, presentation, or other scientific work.

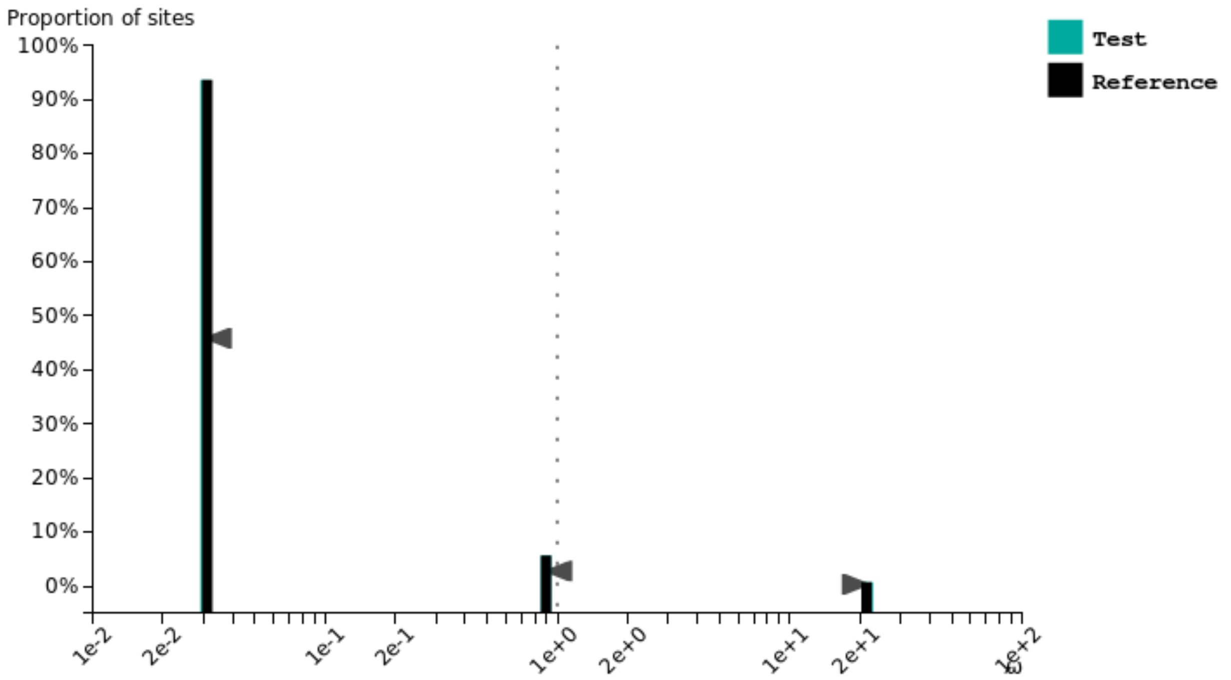

Lucifuga dentata

Test for selection **intensification** (K = **1.07**) was **not significant** (p = **0.487**, LR = **0.48**).

See here for more information about this method.

Please cite PMID 123456789 if you use this result in a publication, presentation, or other scientific work.

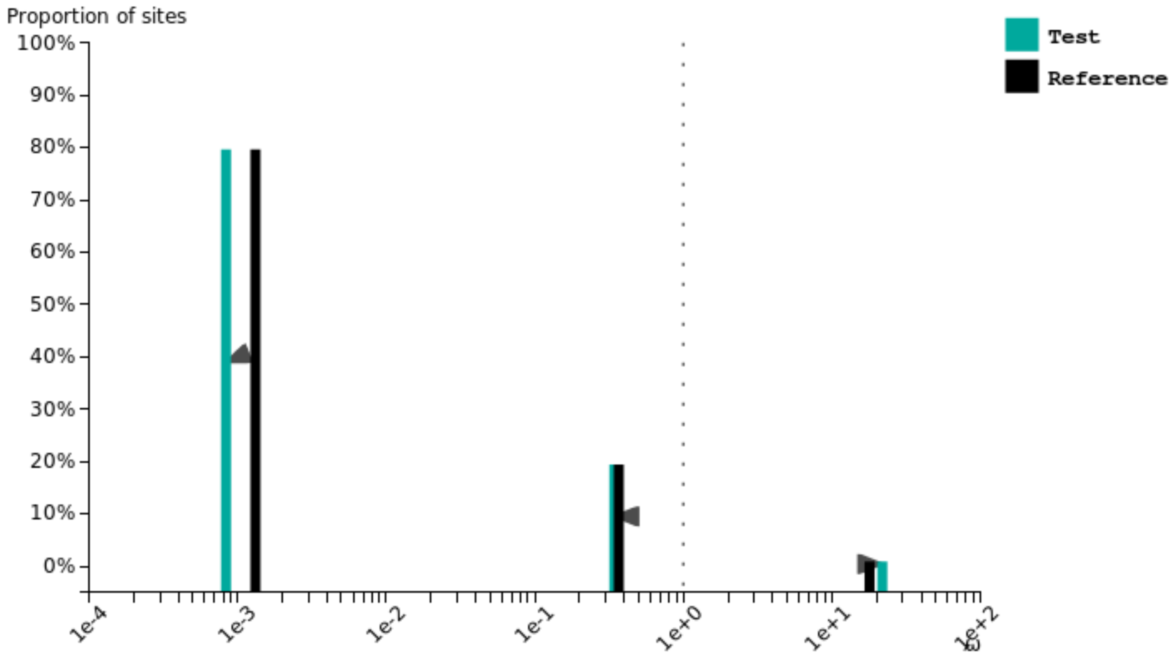

Fig. S7

Astyanax mexicanus CF

Test for selection **relaxation** (K = **1.00**) was **not significant** (p = **1.000**, LR = **0.00**).

See here for more information about this method.

Please cite PMID 123456789 if you use this result in a publication, presentation, or other scientific work.

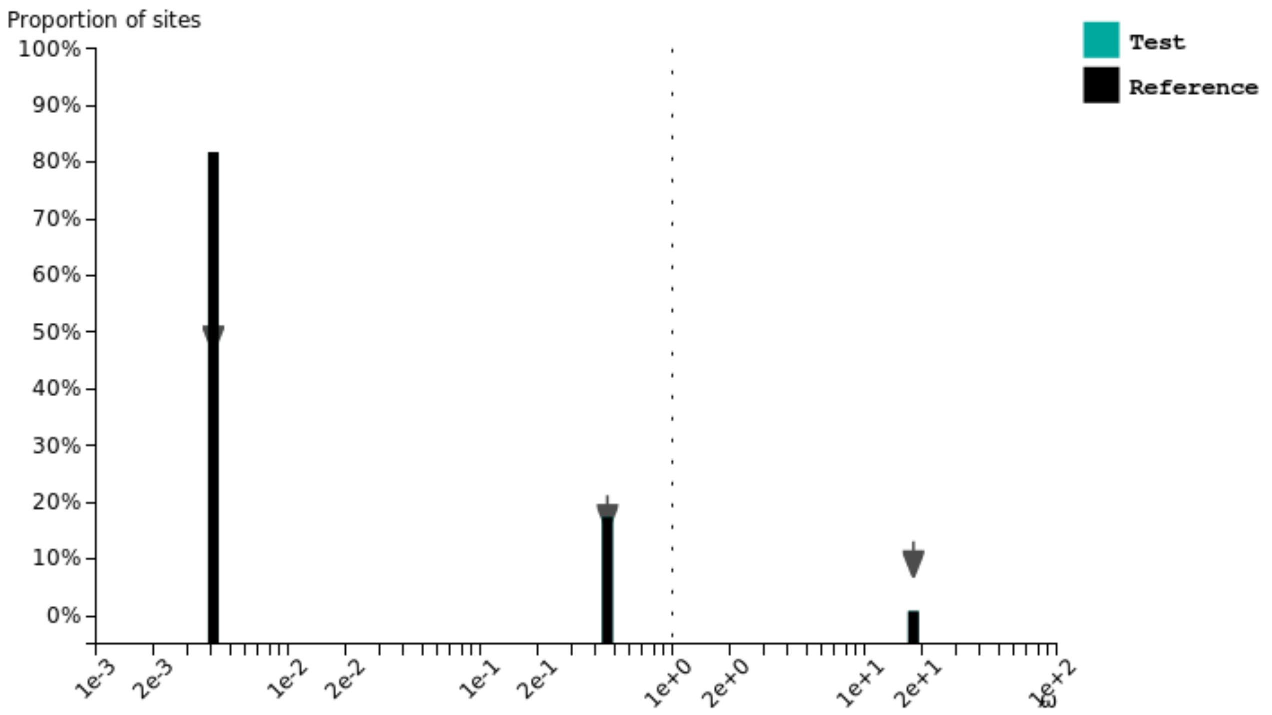

Lucifuga gibarensis

Test for selection **relaxation** (K = **0.75**) was **significant** (p = **0.000**, LR = **33.52**).

See here for more information about this method.

Please cite PMID 123456789 if you use this result in a publication, presentation, or other scientific work.

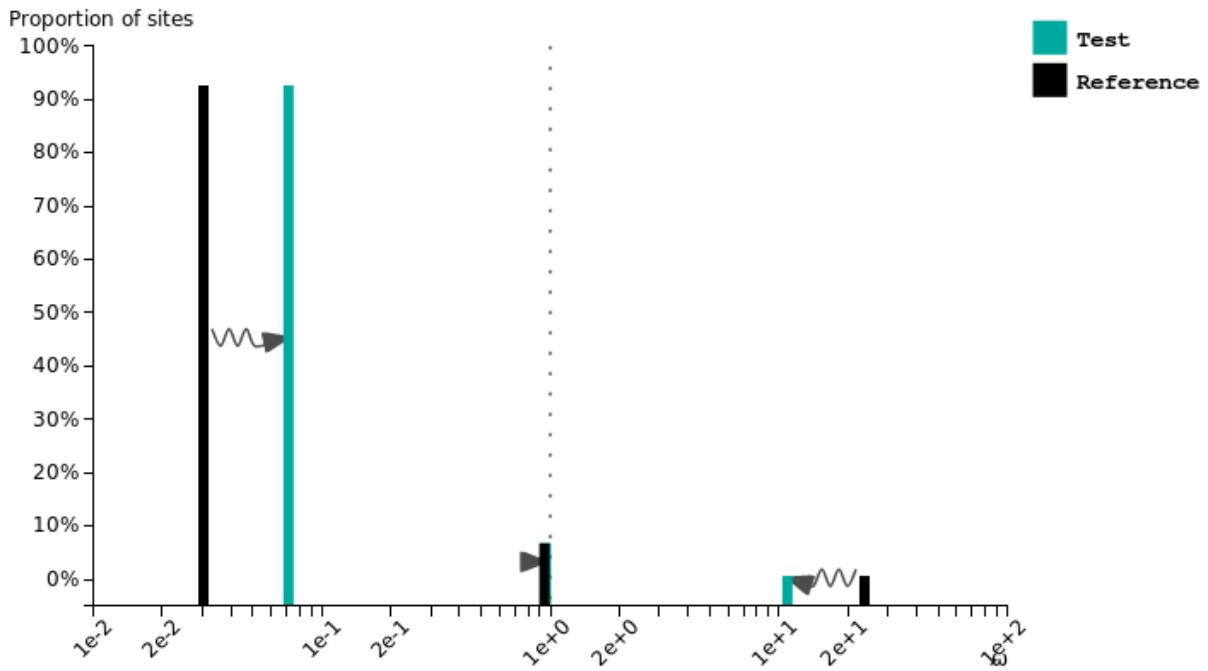

Lucifuga dentata

Test for selection **relaxation** (K = **0.48**) was **significant** (p = **0.000**, LR = **42.53**).

See here for more information about this method.

Please cite PMID 123456789 if you use this result in a publication, presentation, or other scientific work.

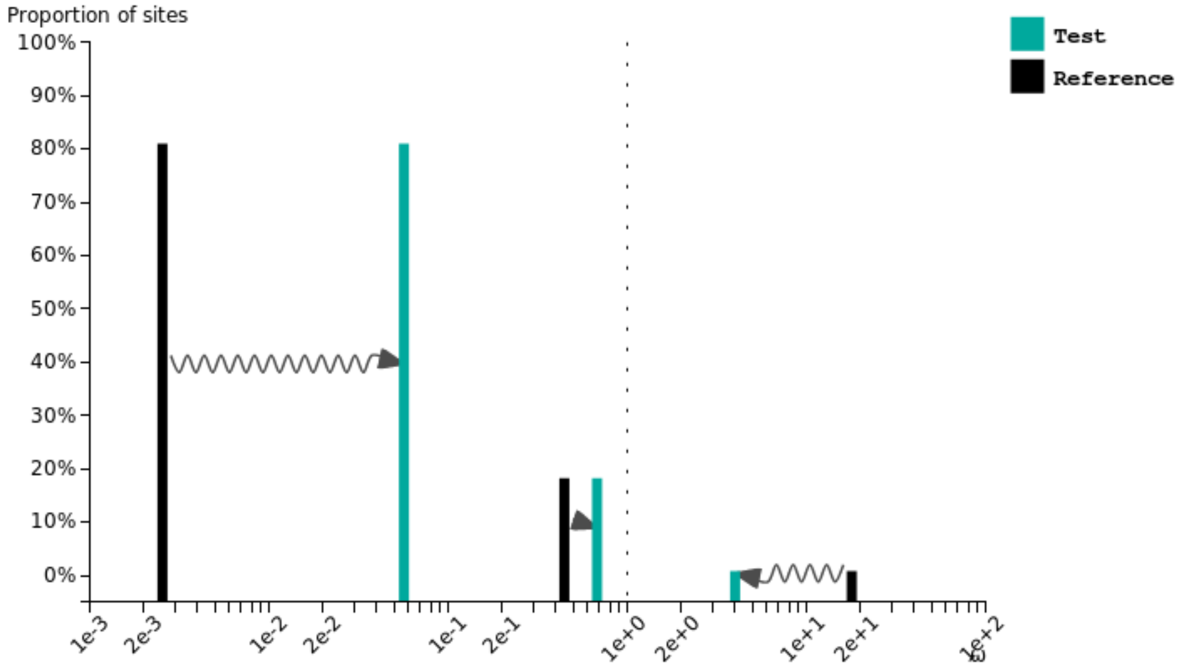

Fig. S8

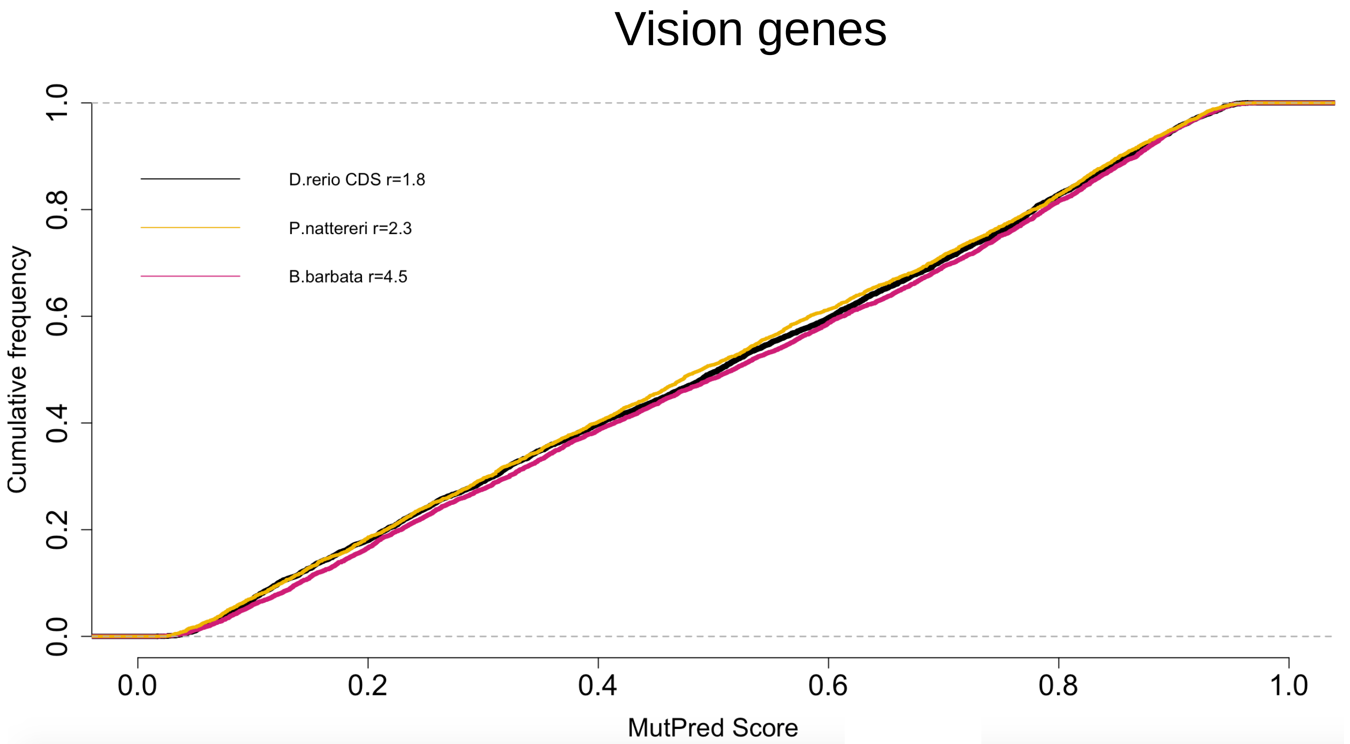
Vision genes
Cumulative frequency
0.0
0.2
0.4
0.6
0.8
1.0
D.rerio CDS r=1.8
P.nattereri r=2.3
B.barbata r=4.5
MutPred Score

Fig. S9

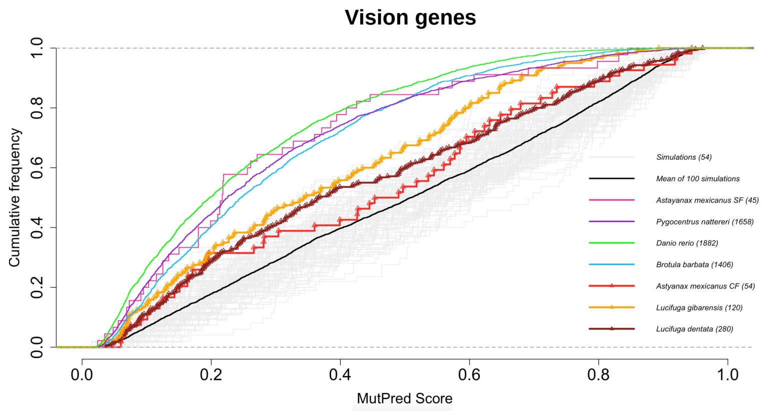

Kolmogorov-Smirnov Tests p-values

	Simu	A.m SF	P.n	D.r	B.b	A.m CF	L.g	L.d
Simu	1.0							
A.m SF	$1e^{-6}$	1.0						
P.n	$3e^{-16}$	0.9	1.0					
D.r	$3e^{-16}$	0.4	$4e^{-3}$	1.0				
B.b	$3e^{-16}$	0.4	$2e^{-2}$	$2e^{-9}$	1.0			
A.m CF	0.2	$9e^{-4}$	$9e^{-6}$	$3e^{-7}$	$3e^{-5}$	1.0		
L.g	$5e^{-6}$	$5e^{-2}$	$5e^{-4}$	$3e^{-6}$	$1e^{-3}$	0.3	1.0	
L.d	$2e^{-5}$	$3e^{-3}$	$9e^{-13}$	$3e^{-16}$	$2e^{-12}$	0.5	0.1	1.0

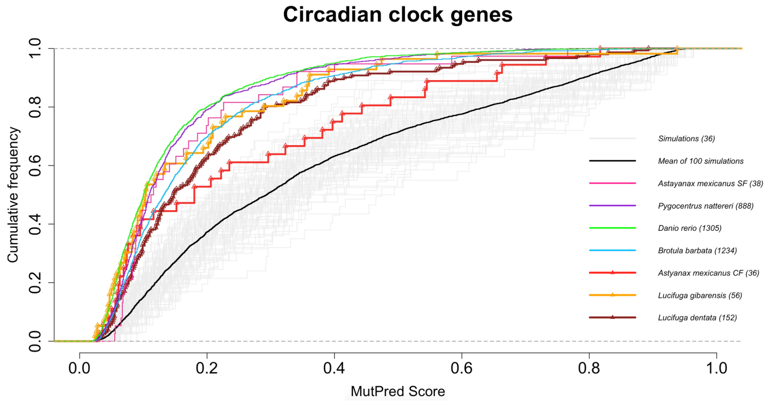

Kolmogorov-Smirnov Tests p-values

	Simu	A.m SF	P.n	D.r	B.b	A.m CF	L.g	L.d
Simu	1.0							
A.m SF	$9e^{-5}$	1.0						
P.n	$3e^{-16}$	0.6	1.0					
D.r	$3e^{-16}$	0.1	$2e^{-4}$	1.0				
B.b	$3e^{-16}$	0.7	$4e^{-7}$	$5e^{-12}$	1.0			
A.m CF	$2e^{-2}$	0.2	$2e^{-2}$	$5e^{-3}$	0.1	1.0		
L.g	$2e^{-6}$	0.3	0.3	0.2	0.2	0.3	1.0	
L.d	$2e^{-9}$	0.4	$1e^{-4}$	$2e^{-5}$	0.3	0.2	0.1	1.0

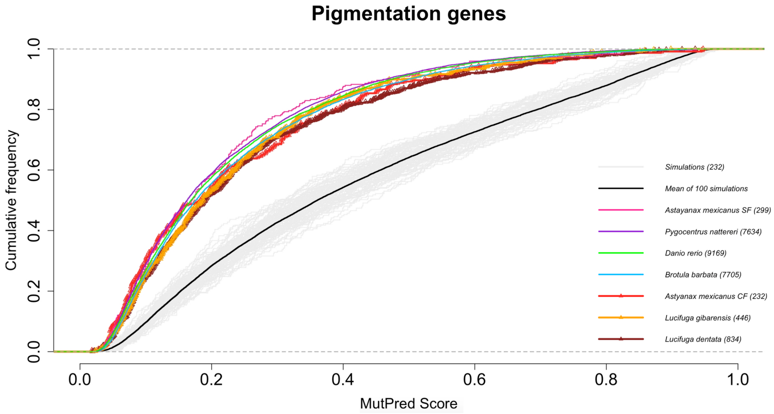

Kolmogorov-Smirnov Tests p-values

	Simu	A.m SF	P.n	D.r	B.b	A.m CF	L.g	L.d
Simu	1.0							
A.m SF	$3e^{-16}$	1.0						
P.n	$3e^{-16}$	0.8	1.0					
D.r	$3e^{-16}$	0.6	$3e^{-3}$	1.0				
B.b	$3e^{-16}$	0.2	$7e^{-6}$	$3e^{-2}$	1.0			
A.m CF	$3e^{-16}$	0.1	0.2	0.2	0.6	1.0		
L.g	$3e^{-16}$	0.2	$3e^{-2}$	0.1	0.5	0.4	1.0	
L.d	$3e^{-16}$	0.1	$2e^{-4}$	$6e^{-3}$	0.1	0.3	0.5	1.0

Fig. S10

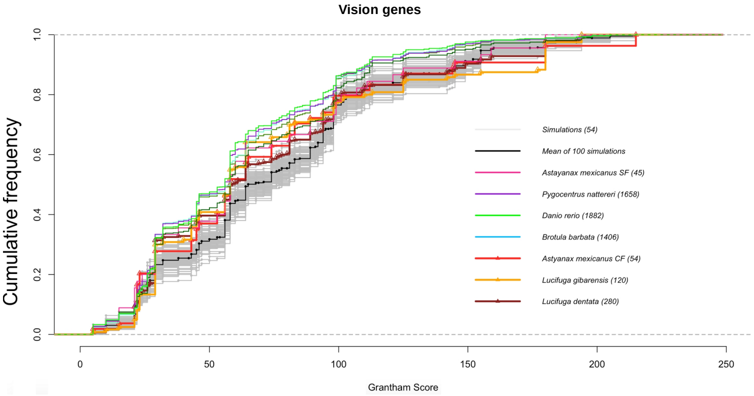

Kolmogorov-Smirnov Tests p-values

	Simu	A.m SF	P.n	D.r	B.b	A.m CF	L.g	L.d
Simu	1.0							
A.m SF	0.4	1.0						
P.n	$3e^{-16}$	0.9	1.0					
D.r	$3e^{-16}$	0.7	0.7	1.0				
B.b	$3e^{-16}$	0.9	0.4	$5e^{-2}$	1.0			
A.m CF	0.4	1.0	0.6	0.4	0.8	1.0		
L.g	$2e^{-2}$	0.9	0.1	$5e^{-2}$	0.1	1.0	1.0	
L.d	$2e^{-2}$	1.0	$3e^{-3}$	$4e^{-4}$	$2e^{-2}$	1.0	0.6	1.0

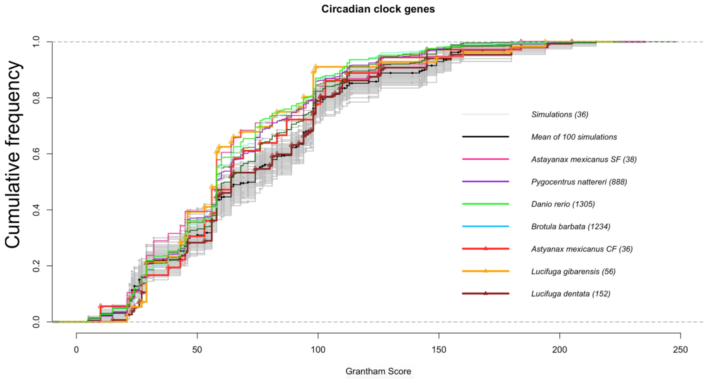

Kolmogorov-Smirnov Tests p-values

	Simu	A.m SF	P.n	D.r	B.b	A.m CF	L.g	L.d
Simu	1.0							
A.m SF	0.1	1.0						
P.n	$2e^{-14}$	0.9	1.0					
D.r	$3e^{-16}$	1.0	0.1	1.0				
B.b	$3e^{-16}$	0.9	1.0	0.2	1.0			
A.m CF	0.6	0.8	1.0	0.7	1.0	1.0		
L.g	$4e^{-2}$	1.0	0.5	0.7	0.7	0.7	1.0	
L.d	0.3	0.4	$1e^{-2}$	$3e^{-4}$	$6e^{-3}$	0.8	0.2	1.0

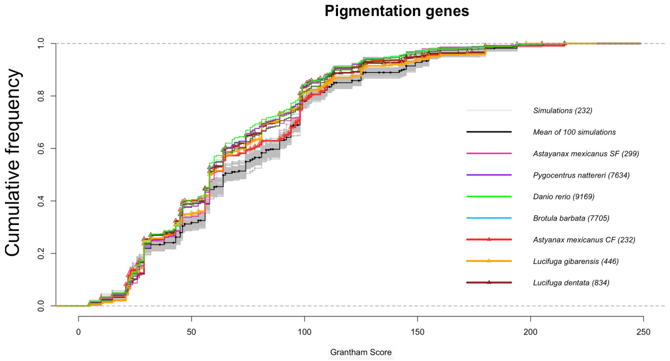

Kolmogorov-Smirnov Tests p-values

	Simu	A.m SF	P.n	D.r	B.b	A.m CF	L.g	L.d
Simu	1.0							
A.m SF	$1e^{-2}$	1.0						
P.n	$3e^{-16}$	0.4	1.0					
D.r	$3e^{-16}$	0.1	$7e^{-3}$	1.0				
B.b	$3e^{-16}$	0.5	0.1	$3e^{-2}$	1.0			
A.m CF	$4e^{-2}$	0.7	0.1	$2e^{-2}$	0.1	1.0		
L.g	$5e^{-5}$	0.8	0.3	0.1	0.3	0.1	1.0	
L.d	$2e^{-8}$	0.5	0.2	$4e^{-2}$	0.1	0.1	0.7	1.0

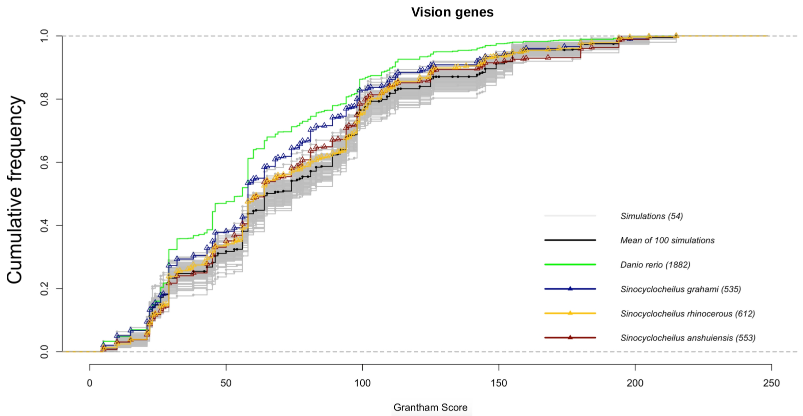

Kolmogorov-Smirnov Tests p-values

	Simu	D.r	S.g	S.r	S.a
Simu	1.0				
D.r	$3e^{-16}$	1.0			
S.g	$5e^{-7}$	$5e^{-4}$	1.0		
S.r	0.1	$2e^{-9}$	$2e^{-3}$	1.0	
S.a	0.09	$4e^{-9}$	0.1	0.7	1.0

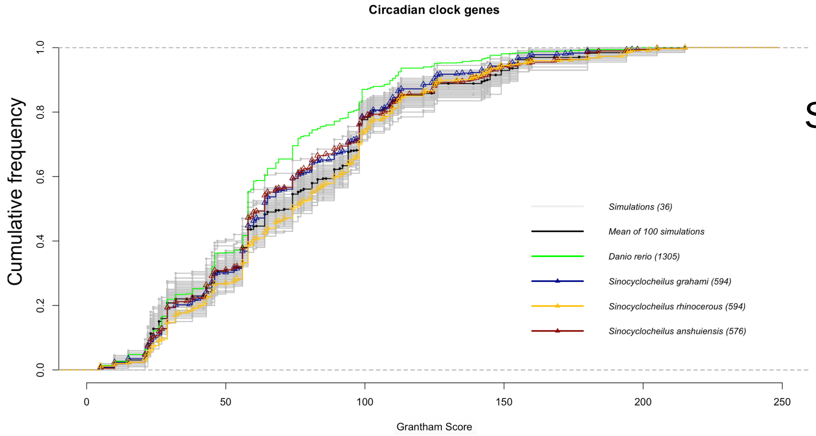

Kolmogorov-Smirnov Tests p-values

	Simu	D.r	S.g	S.r	S.a
Simu	1.0				
D.r	$3e^{-16}$	1.0			
S.g	$2e^{-2}$	$9e^{-6}$	1.0		
S.r	$2e^{-2}$	$3e^{-15}$	$7e^{-3}$	1.0	
S.a	$3e^{-3}$	$2e^{-4}$	1.0	$1e^{-3}$	1.0

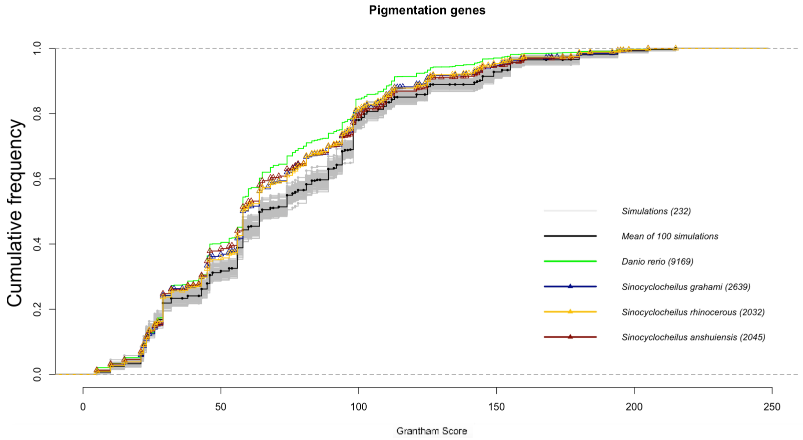

Kolmogorov-Smirnov Tests p-values

	Simu	D.r	S.g	S.r	S.a
Simu	1.0				
D.r	$3e^{-16}$	1.0			
S.g	$4e^{-16}$	$4e^{-6}$	1.0		
S.r	$2e^{-13}$	$2e^{-5}$	1.0	1.0	
S.a	$7e^{-16}$	$2e^{-4}$	0.6	0.4	1.0

Fig. S11

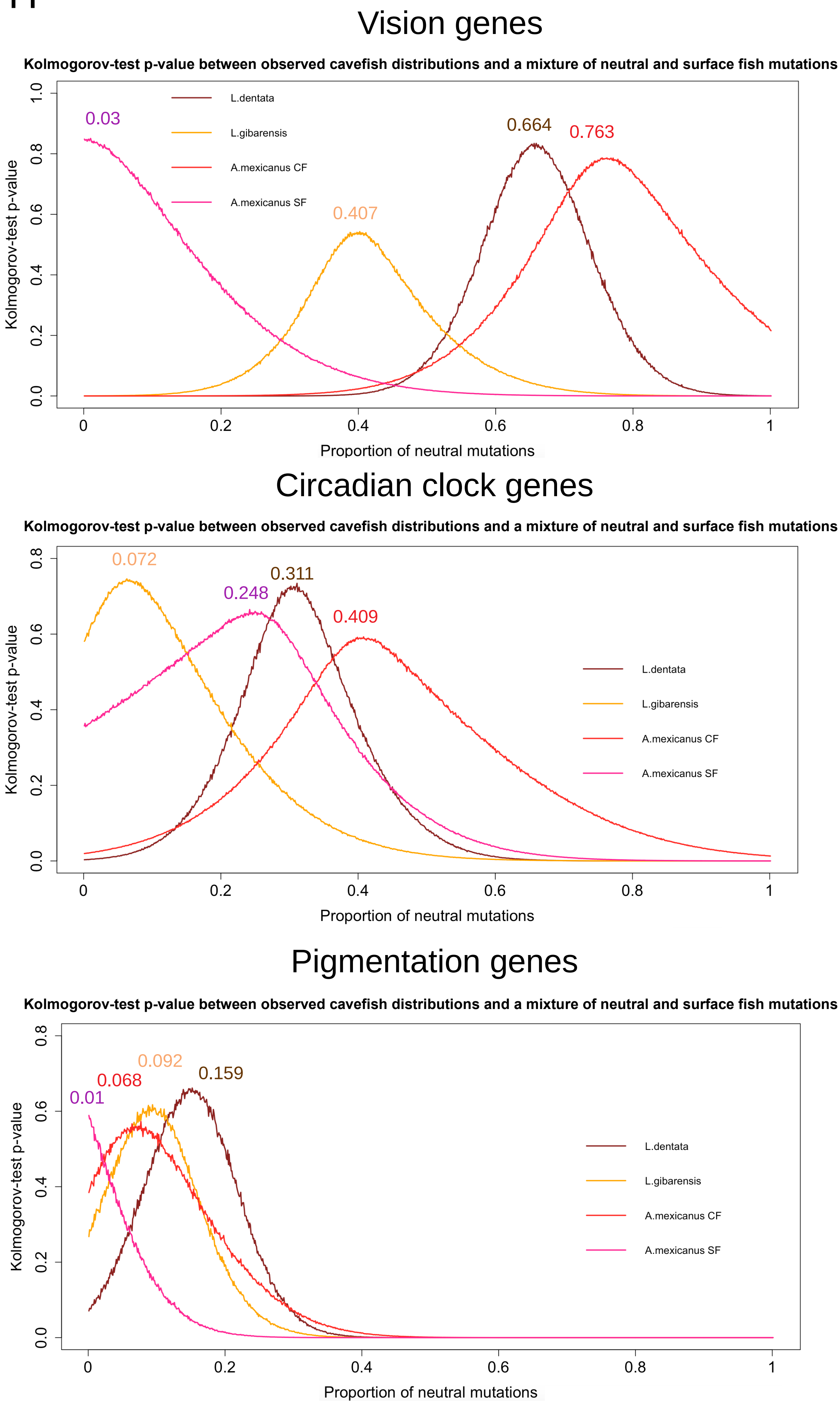
Vision genes
Kolmogorov-test p-value between observed cavefish distributions and a mixture of neutral and surface fish mutations
Kolmogorov-test p-value
Proportion of neutral mutations
L.dentata
L.gibarensis
A.mexicanus CF
A.mexicanus SF
0.03
0.407
0.664
0.763
Circadian clock genes
Kolmogorov-test p-value between observed cavefish distributions and a mixture of neutral and surface fish mutations
0.072
0.311
0.248
0.409
Pigmentation genes
Kolmogorov-test p-value between observed cavefish distributions and a mixture of neutral and surface fish mutations
0.092
0.159
0.068
0.01

Fig. S12

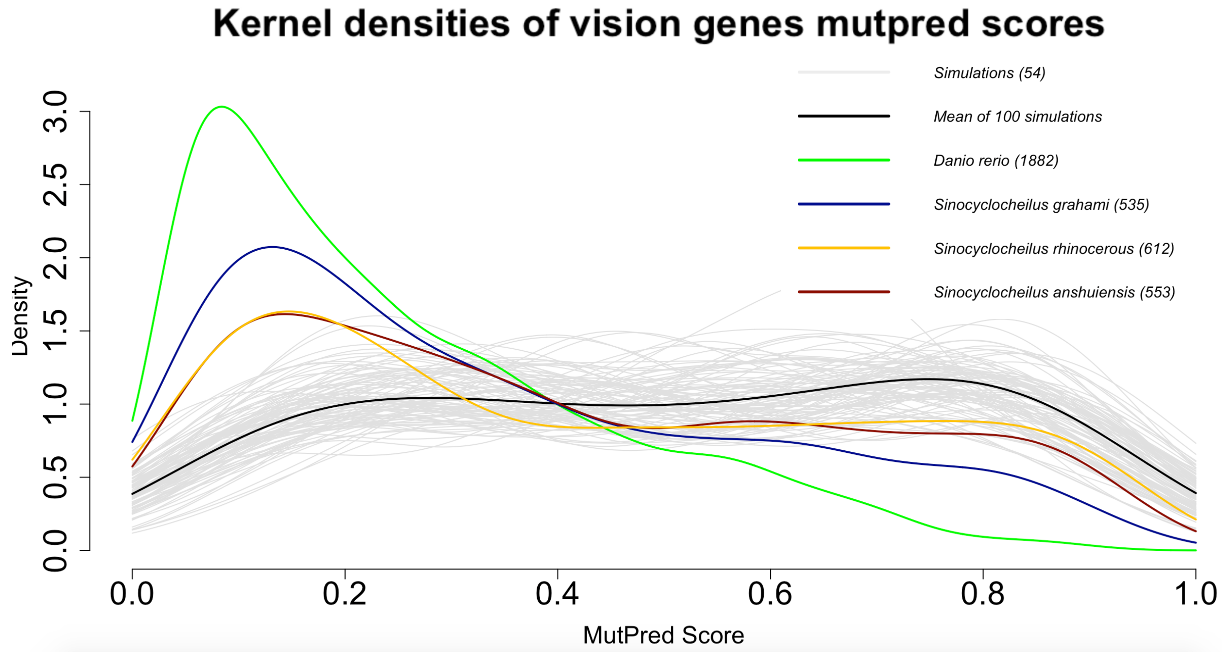
Kernel densities of vision genes mutpred scores
Simulations (54)
Mean of 100 simulations
Danio rerio (1882)
Sinocyclocheilus grahami (535)
Sinocyclocheilus rhinocerous (612)
Sinocyclocheilus anshuiensis (553)
Density
0.0
0.5
1.0
1.5
2.0
2.5
3.0
0.0
0.2
0.4
0.6
0.8
1.0
MutPred Score

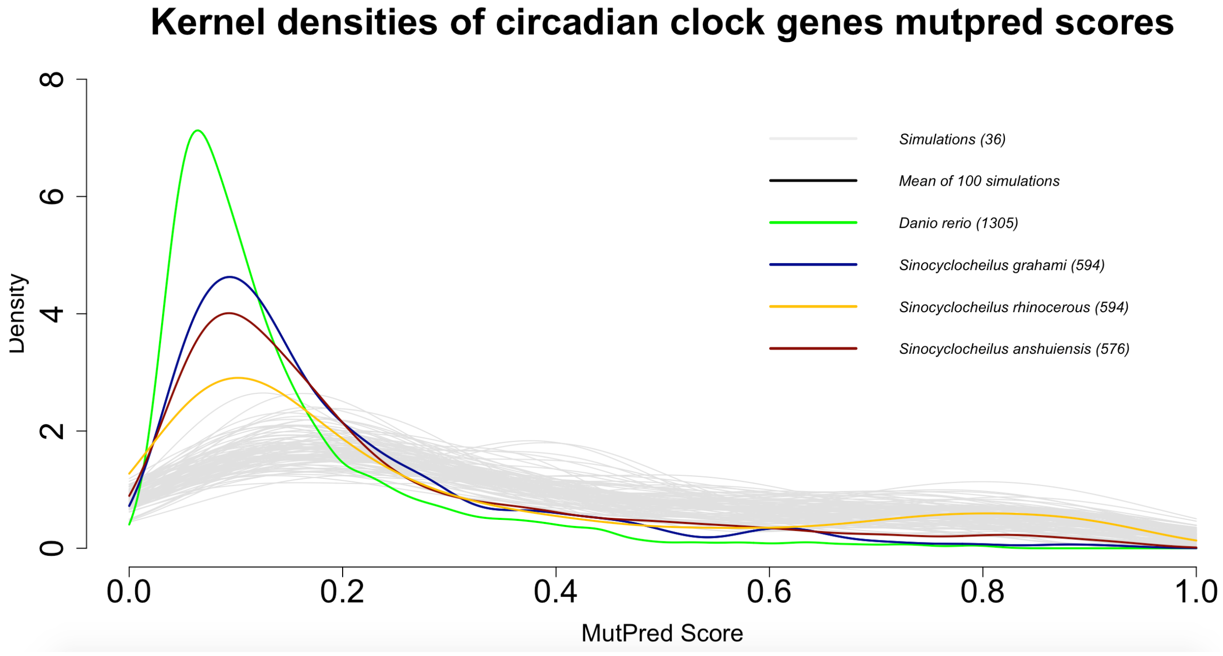
Kernel densities of circadian clock genes mutpred scores
Simulations (36)
Mean of 100 simulations
Danio rerio (1305)
Sinocyclocheilus grahami (594)
Sinocyclocheilus rhinocerous (594)
Sinocyclocheilus anshuiensis (576)
Density
0
2
4
6
8
0.0
0.2
0.4
0.6
0.8
1.0
MutPred Score

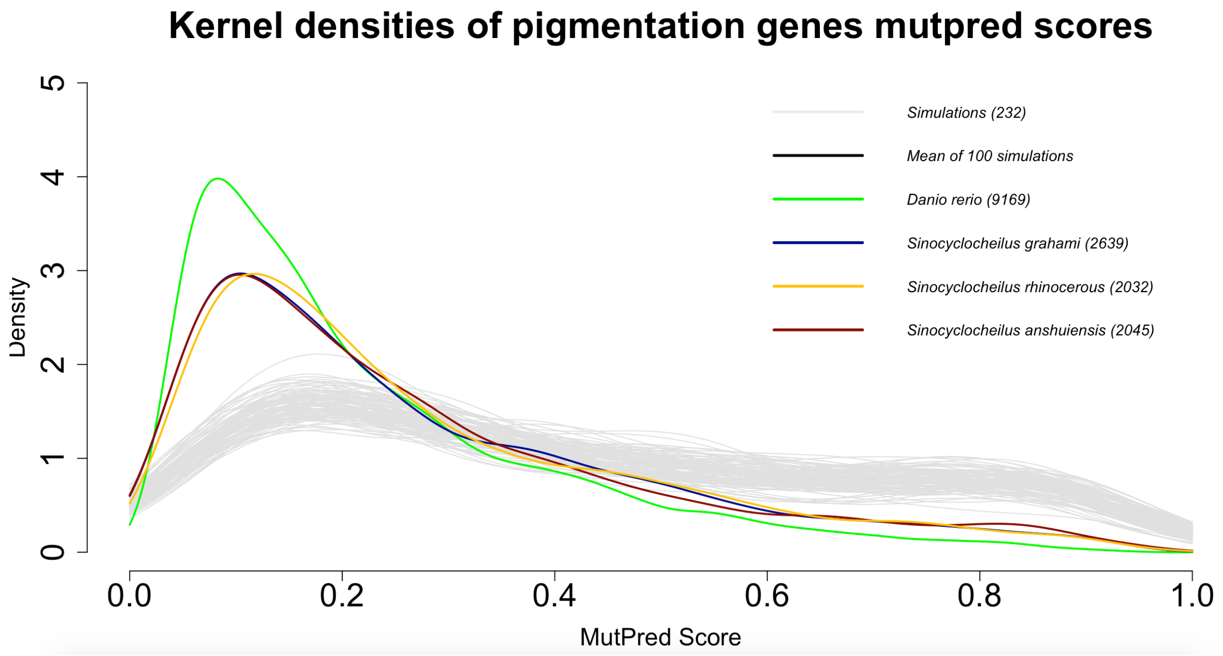
Kernel densities of pigmentation genes mutpred scores
Simulations (232)
Mean of 100 simulations
Danio rerio (9169)
Sinocyclocheilus grahami (2639)
Sinocyclocheilus rhinocerous (2032)
Sinocyclocheilus anshuiensis (2045)
Density
0
1
2
3
4
5
0.0
0.2
0.4
0.6
0.8
1.0
MutPred Score

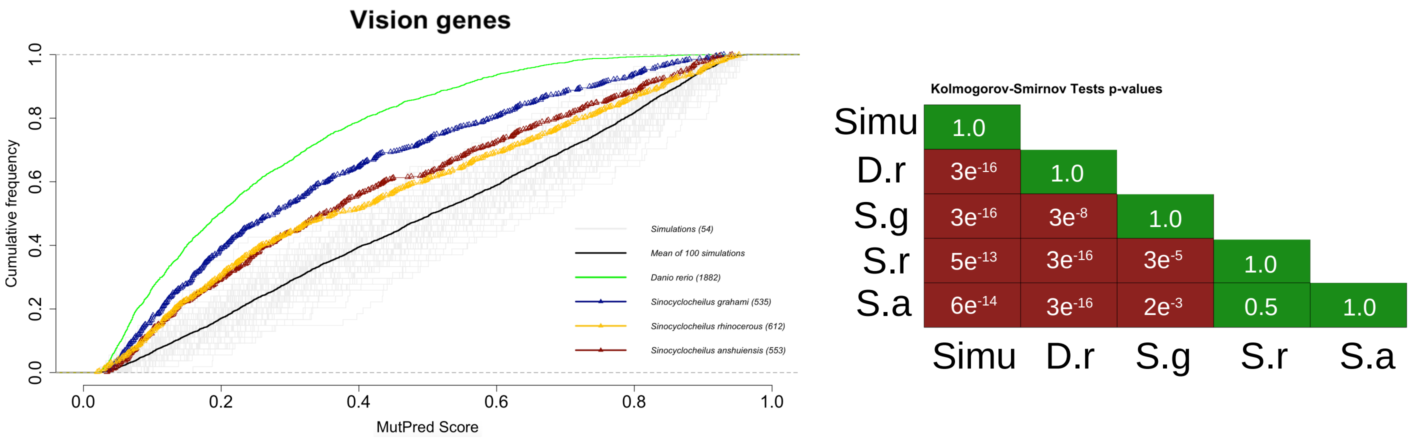
Vision genes
Cumulative frequency
MutPred Score
Simulations (54)
Mean of 100 simulations
Danio rerio (1882)
Sinocyclocheilus grahami (535)
Sinocyclocheilus rhinocerous (612)
Sinocyclocheilus anshuiensis (553)
Kolmogorov-Smirnov Tests p-values
Simu 1.0
D.r 3e-16 1.0
S.g 3e-16 3e-8 1.0
S.r 5e-13 3e-16 3e-5 1.0
S.a 6e-14 3e-16 2e-3 0.5 1.0
Simu D.r S.g S.r S.a

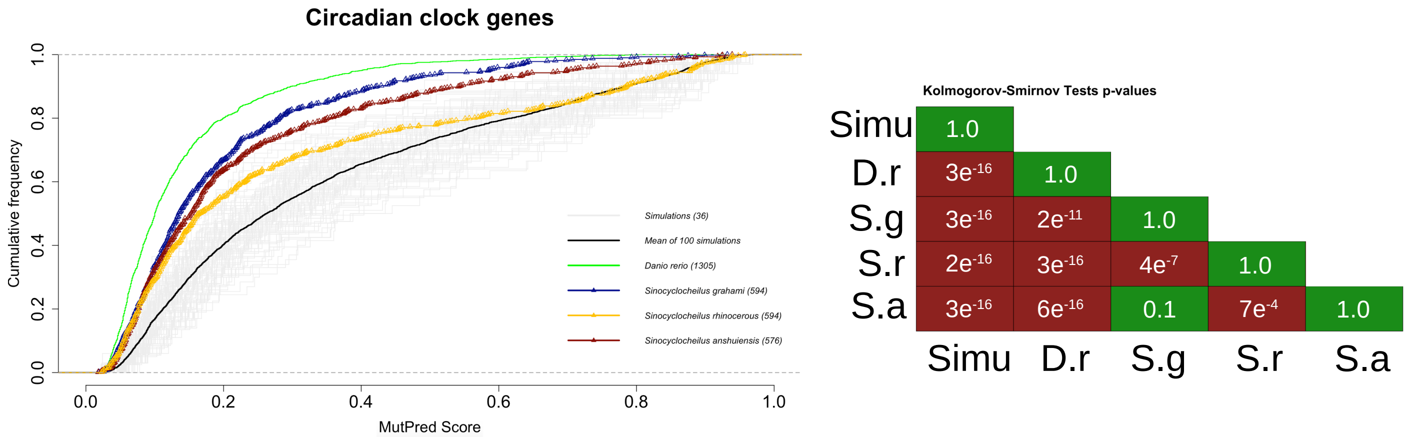
Circadian clock genes
Cumulative frequency
MutPred Score
Simulations (36)
Mean of 100 simulations
Danio rerio (1305)
Sinocyclocheilus grahami (594)
Sinocyclocheilus rhinocerous (594)
Sinocyclocheilus anshuiensis (576)
Kolmogorov-Smirnov Tests p-values
Simu 1.0
D.r 3e-16 1.0
S.g 3e-16 2e-11 1.0
S.r 2e-16 3e-16 4e-7 1.0
S.a 3e-16 6e-16 0.1 7e-4 1.0
Simu D.r S.g S.r S.a

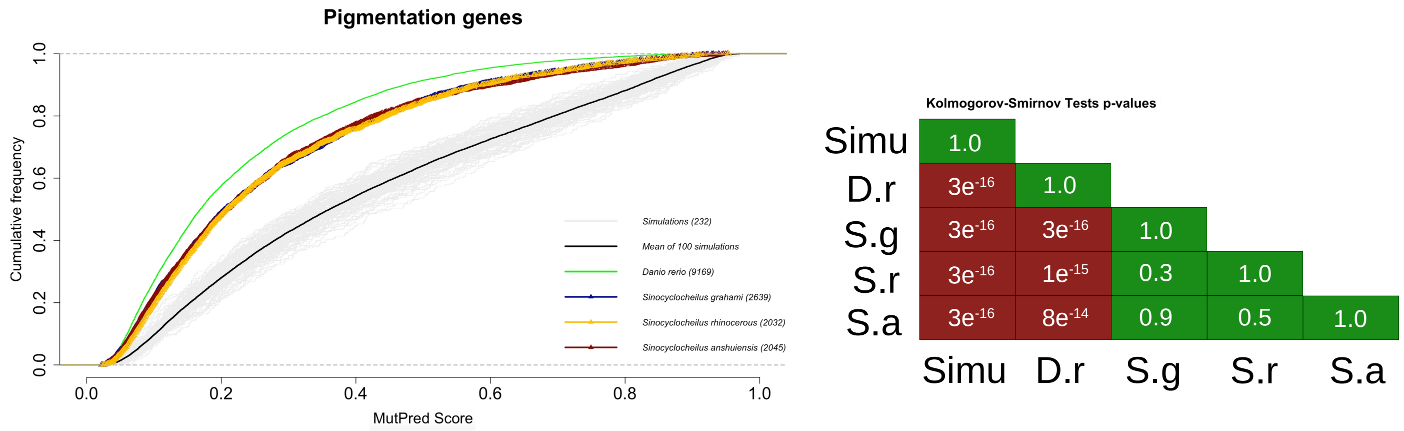
Pigmentation genes
Cumulative frequency
MutPred Score
Simulations (232)
Mean of 100 simulations
Danio rerio (9169)
Sinocyclocheilus grahami (2639)
Sinocyclocheilus rhinocerous (2032)
Sinocyclocheilus anshuiensis (2045)
Kolmogorov-Smirnov Tests p-values
Simu 1.0
D.r 3e-16 1.0
S.g 3e-16 3e-16 1.0
S.r 3e-16 1e-15 0.3 1.0
S.a 3e-16 8e-14 0.9 0.5 1.0
Simu D.r S.g S.r S.a

Fig. S13

Vision genes

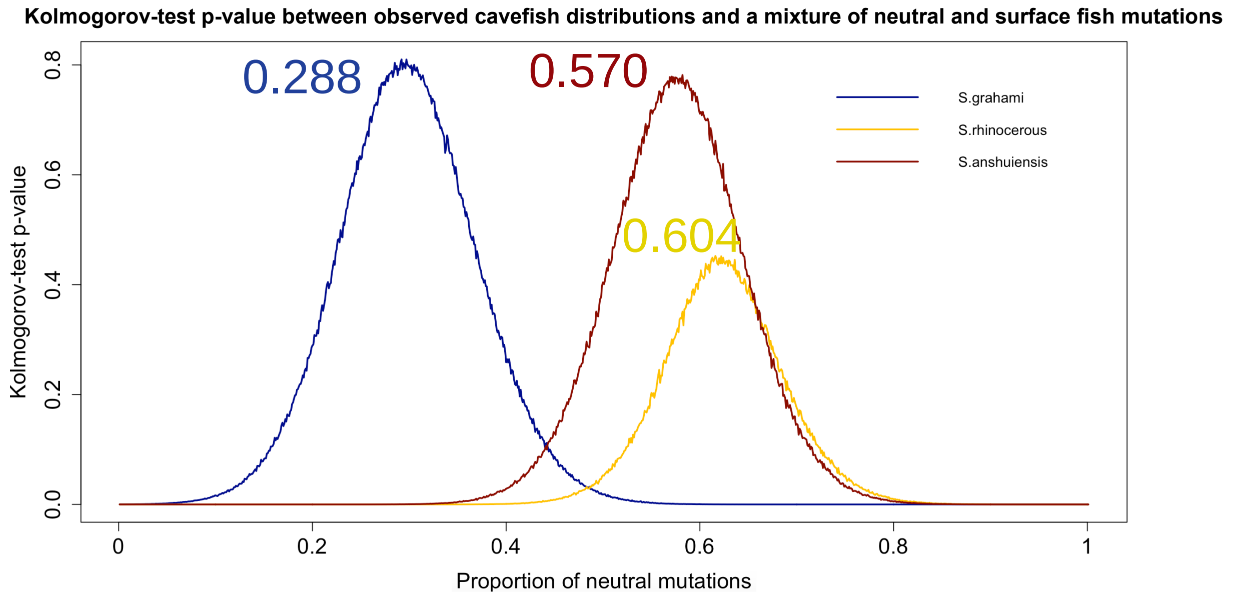

Circadian clock genes

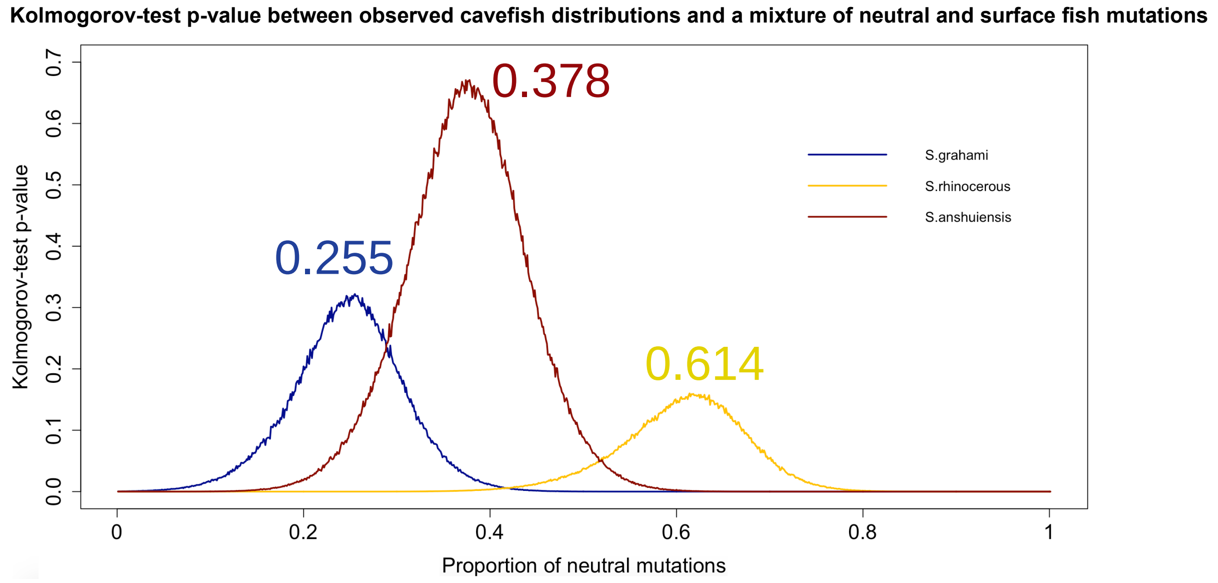

Pigmentation genes

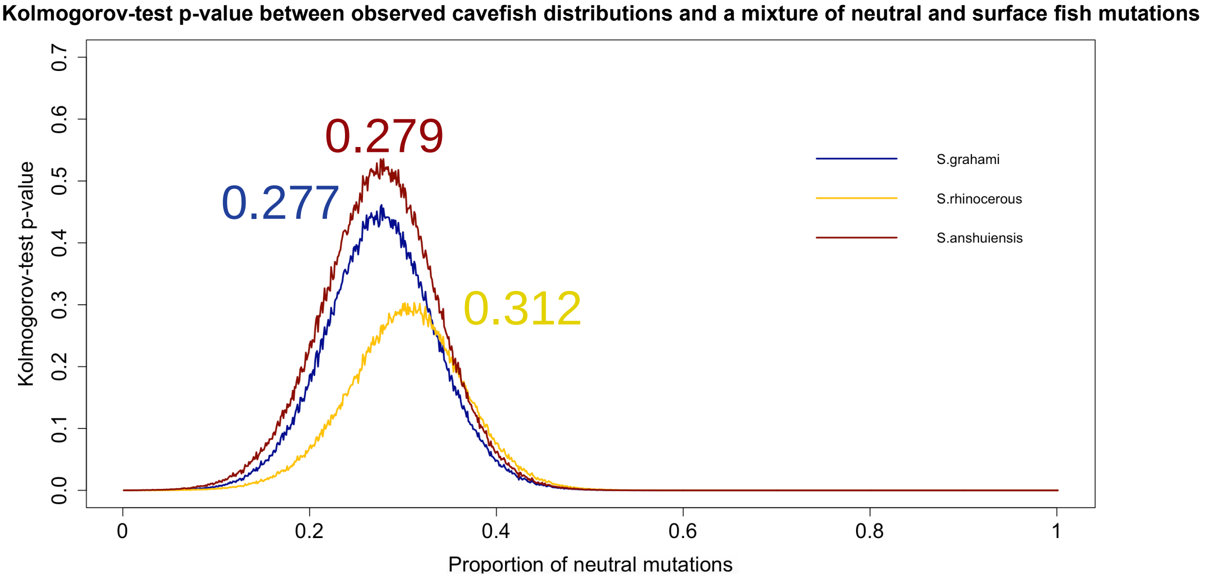

Supplement: msaa249_Supplementary_Data [file msaa249_supplementary_data.zip › msaa249-suppl_data/Supp_mat_tables_figures.pdf]
